# Physical activity among children and young people with chronic kidney diseases: a scoping review

**DOI:** 10.1007/s00467-025-07142-0

**Published:** 2026-04-06

**Authors:** Rui Zhao, Minghui Yu, Richard J. M. Coward, Nick Townsend, Lucy Plumb

**Affiliations:** 1 Bristol Medical School, University of Bristol, Bristol, BS8 1UD UK; 2 Nephrology Department, Children’s Hospital of Fudan University, Shanghai, 201102 China; 3 School for Policy Studies, University of Bristol, Bristol, BS8 1TZ UK

**Keywords:** Physical activity, Children, Adolescent, Chronic kidney disease, Scoping review

## Abstract

Promoting physical activity is a global initiative. However, published systematic syntheses regarding physical activity among children and young people with chronic kidney disease (CKD) are limited. The aim of this review was to determine the evidence relating to physical activity among children and young people with CKD and identify research gaps. A comprehensive search across seven databases was conducted, and two reviewers screened and synthesised the data. Fifty-seven primary studies were included. Most studies were observational, conducted in Europe and North America, and focused on kidney transplant recipients. Physical activity levels measured by various parameters, tools, and reporting methods showed children with CKD had a lower or similar percentage of physical activity participation, intensity, frequency, duration, and overall activeness compared with healthy controls. Associations between physical activity and sex, age, CKD stages, and outcomes varied in size, direction, and strength among studies. No prospective longitudinal studies have examined the associations between physical activity and CKD progression. Nor did any explicitly describe children’s/carer’s experience of engaging in physical activity, although five qualitative studies reported impacts and adaptations in children with different CKD stages. Most interventions described were short-term (<6 months) home-based exercise training programmes incorporating endurance, resistance, or flexibility elements. In conclusion, evidence on physical activity in children with CKD is predominantly quantitative, cross-sectional, relying on self-reported data, and focusing primarily on adolescents and kidney transplant recipients. Further research should establish more precise physical activity estimates using validated, standardised measures across CKD stages and diverse populations.

**Graphical abstract:**

A higher resolution version of the Graphical abstract is available as Supplementary information

**Figure:**
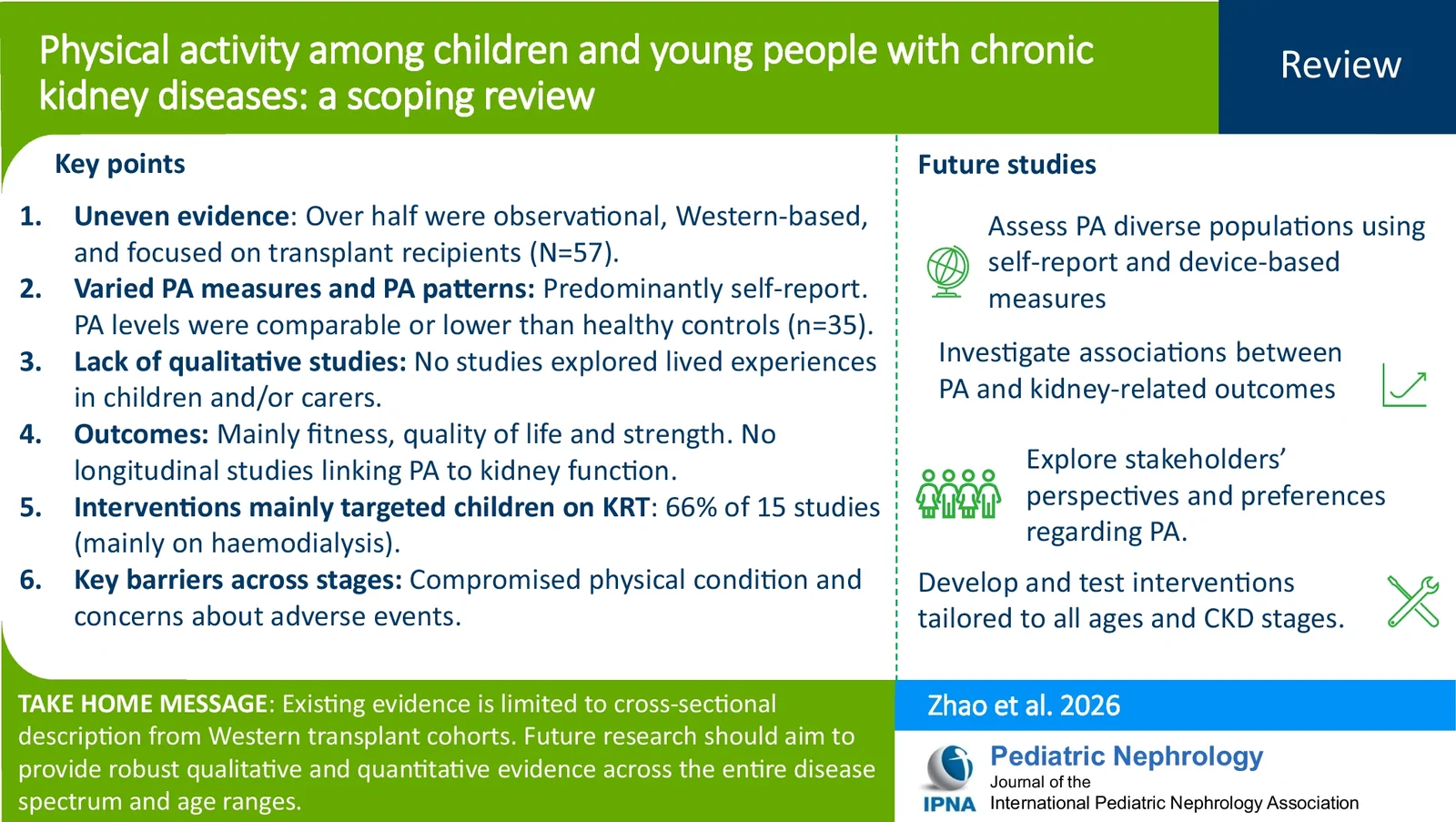

**Supplementary Information:**

The online version contains supplementary material available at https://doi.org/10.1007/s00467-025-07142-0.

## Background

Physical activity (PA) refers to any form of bodily movement performed by skeletal muscles that results in increased energy expenditure [1]. Promoting PA has been a global initiative for people of all ages and abilities with established evidence that regular PA lowers the risk of cardiovascular diseases, metabolic disorders, and cancer whilst improving mental health and quality of life [1, 2]. In adults with certain chronic diseases, engaging in PA has been associated with reduced mortality as well as slow cardiovascular disease (CVD) progression, without accelerating the progression of primary disease [1, 3]. Among children, a higher PA level is additionally related to improved academic performance, school attendance, cognitive function, and classroom behaviour [4].

Childhood chronic kidney disease (CKD) is relatively uncommon. The reported paediatric CKD incidence varies greatly across the globe from 5 to 18 per million of the age-related population (pmarp) [5] due to varying inclusion criteria of CKD stages and age ranges. It is characterised by impaired kidney function, in some cases leading to kidney replacement therapy (KRT). CKD in children is associated with significant comorbidities, including growth and cognitive impairment, and substantially elevates cardiovascular risk and mortality, particularly in children with kidney failure [6].

PA is a key modifiable factor for cardiovascular health and living well with CKD [2]. The clinical practice guideline of exercise and lifestyle in CKD from the UK Kidney Association is the first of its kind to offer practical recommendations to people with non-dialysis chronic kidney disease, on dialysis, and post-transplantation [2]. However, much of the supporting evidence is derived from adult studies. These findings cannot be directly applied to children, since the disease processes underlying CKD development in adults and children are very different in terms of aetiology, epidemiology, treatment, and progression [7]. In the context of promoting PA, it is important to provide all children and adolescents with safe and equitable opportunities to participate in physical activities that are appropriate for their age and ability, that are enjoyable, and that offer variety [1]. It is even more essential that children with CKD are not left behind and are supported to experience the benefits of PA.

The aim of this scoping review was to identify, collate, and summarise the evidence regarding PA among children and young people with CKD and to identify research gaps to inform future research. Key topics included PA levels and patterns, PA participation experience, barriers and facilitators to PA participation, PA intervention, and PA-related health outcomes.

## Methods

The Joanna Briggs Institute (JBI) approach and Preferred Reporting Items of Systematic Reviews and Meta-Analyses (PRISMA) scoping review extension (RISMA-ScR) [8] were used to conduct this scoping review [9]. The review protocol was published on Open Science Framework https://osf.io/3sgkw/. Table 1 highlights the inclusion criteria used for study selection. For pragmatic reasons, we chose to include all stages of CKD in the review.

**Table 1:** PCC (Population, Concept, and Context) inclusion and exclusion criteria

	Inclusion criteria	Exclusion criteria
Population	Children and young people with CKD by any definition aged below 25 years	CKD is not the main diagnosis of participants in studies
Concept	Levels, patterns, related factors, outcomes of PA; feasibility or effectiveness of intervention of physical activities; experience, barriers and facilitators of being active	Studies only focusing on quality of life, physical functioning or exercise capacities Studies testing bundle interventions
Context	All settings including but not limited to communities, schools, and hospitals	
Study design	All study designs including primary and secondary studies, qualitative and quantitative studies	
Others	No language limitation	

An initial search was conducted in MEDLINE and the Physiotherapy Evidence Database (PEDro) with search strategies developed by a research librarian using NICE guidelines and the Cochrane Systematic Reviews search filters. A comprehensive search was then conducted in the following databases: the Cochrane Central Register of Controlled Trials (CENTRAL in Cochrane Library), MEDLINE Ovid, Embase Ovid, CINAHL/EBSCO, PEDro, PsycINFO Ovid, and Web of Science. Studies published in English or non-English during database inception to February 2024 (Supplementary Material 1) were included. Reference lists of secondary studies were examined to identify additional original studies. Grey literature was searched using ISRCTN Registry (www.isrctn.com), Clinicaltrials.gov (https://clinicaltrials.gov), International Society for Peritoneal Dialysis (https://ispd.org/guidelines/), and the UK Kidney Association (https://ukkidney.org/health-professionals/guidelines/guidelines-commentaries) (Supplementary Material 1).

Retrieved citations were imported into Endnote 21™. After removing duplicates, two independent reviewers (RZ and MHY) screened titles and abstracts, followed by full texts, for eligibility. A second reviewer (MHY) independently screened 127 papers (20% of the original search), achieving 96.3% agreement. Disagreement on the eligibility of studies was solved by consultation with other authors (LP and NT). Data were extracted using a standard form with consensus among all reviewers (Supplementary Table 2). A second reviewer (MHY) performed independent data extraction for 20% of studies with 100% agreement between reviewers. Statements from guidelines, protocols, book chapters, or opinions that could not be traced with primary studies were excluded from data extraction. An updated search was conducted from the last search to 12 September 2025 using the same strategy and databases. Duplicate removals, screening, and data extraction were conducted using the same procedure as in the original search.

## Results

**Fig. 1:**
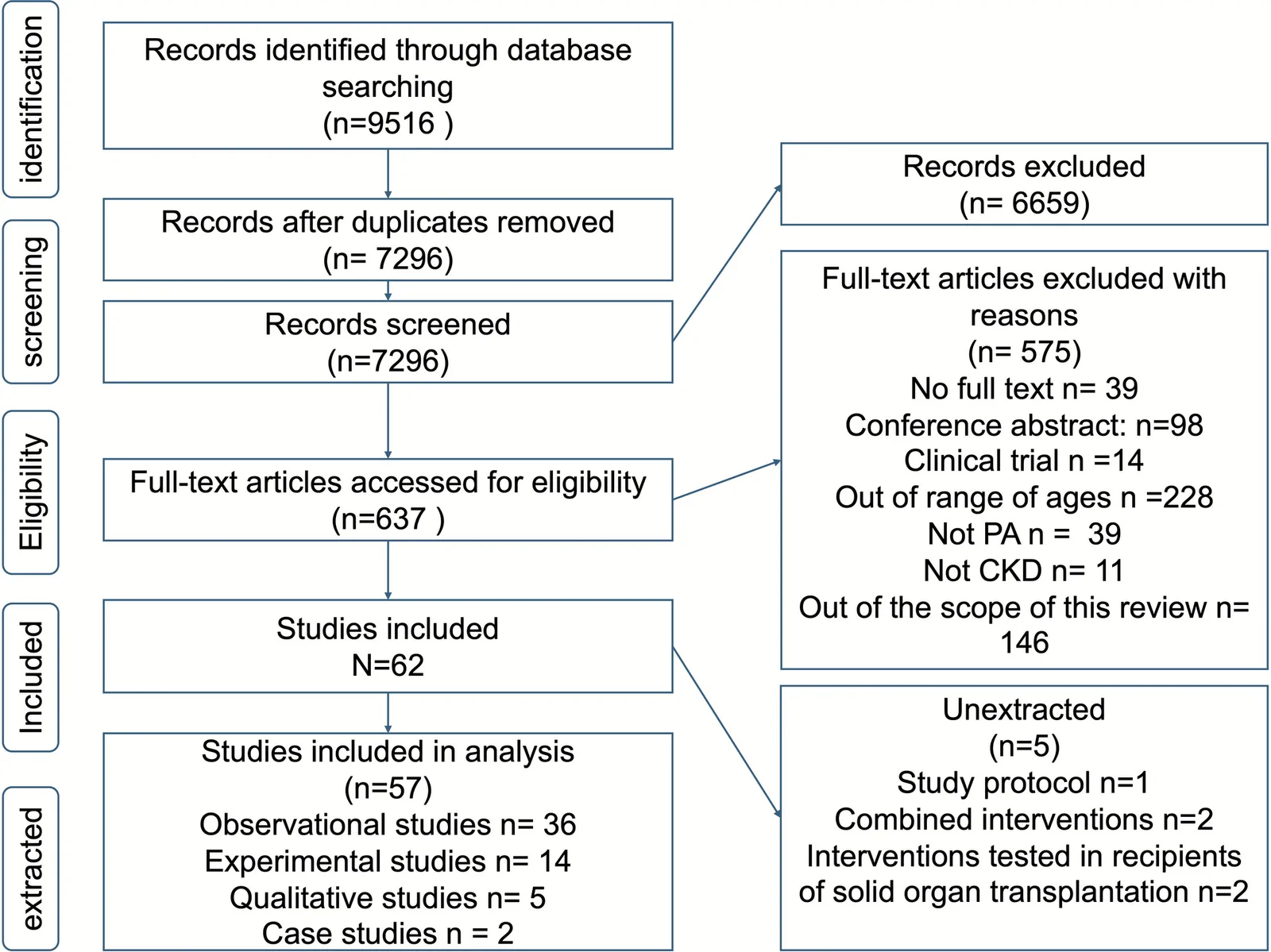
PRISMA-style flow diagram of study inclusion

Fifty-seven studies from 9516 papers identified through literature searches were included in this review (Table 2). A PRISMA-style flow diagram of study inclusion is presented in Fig. 1.

**Table 2:** Characteristics of the studies in the scoping review

Author and year	Design	CKD stages	Country	Aim	Participants Sample size (*N*), age (years) mean ± SD or median IQR or median range, sex = *n* (%)	Summary of findings Levels of physical activity Experience Intervention Barriers and facilitators
Gerson, 2005 [10]	Cross-sectional study	Across all stages of CKD, including dialysis and kidney transplantation	USA	To assess scores on the CHIP-AE in adolescents with CKD compared with two control groups of age-, socioeconomic-, and gender-matched peers To compare health of children of chronic renal insufficiency, on dialysis and transplant	*N* = 113, 14.2 ± 1.9, M = 72 (64), F = 41 (36)	Overall activeness (The Child Health and Illness Profile-Adolescent Edition (CHIP-AE)) • Mean scores of subdomain physical activity were 17.3 ± 4.6 in children with CKD, lower than 20.7 ± 4.7 in a national sample of 226 age-matched healthy adolescents (*P* < 0.001) • Mean score of subdomain physical activity was 19.3 ± 4.9 in 39 children with non-KRT CKD, higher than 14.2 ± 3.3 in 21 children on dialysis (*P* < 0.05) and higher than 17.0 ± 4.2 in 53 children with transplants (*P* < 0.05). Transplant group reported more physical activity than dialysis group (*P* < 0.05)
Teixeira, 2014 [11]	Cross-sectional study	Across all stages of CKD, including dialysis and kidney transplantation	Brazil	To evaluate the impact of CKD on QoL from the children’s and their parents’ perception, respiratory muscle strength, pulmonary function, and functional capacity	*N* = 40, 13 ± 2.6, M = 21 (52.5), F = 19 (47.5)	Levels of PA • 34 (85%) did not practise any physical activity and were sedentary. 6 (15%) reported to participate in regular activity (≤2 h/week) • Those who performed regular physical activity had a higher score of PedsQL™ (version 3) when compared to sedentary individuals (*P* = 0.002) from the children’s perception
Akber, 2012 [12]	Cross-sectional study	Across all stages of CKD, including dialysis and kidney transplantation	USA	To measure levels of physical activity, physical performance, and physical functioning and to examine potential correlates of physical activity and the association of physical activity with physical performance and self-reported physical functioning among children and young adults with CKD stages 1–4, undergoing haemodialysis or peritoneal dialysis or transplantation	*N* = 44, 15.1 ± 3.4, M = 22 (50), F = 22 (50)	Median steps per day • Median (IQR) steps per day over 7 days were 5976 (3309, 8595), 44% lower than healthy young people • Boys recorded more steps per day than girls, 9122 (6547, 11,951) VS 4027 (2682, 5667) (*P* < 0.001) • 18–20 years participants were less active than younger participants (*P* < 0.05). There was an age-related gradient in steps per day (*P* = 0.03) (no detailed number provided, figure only) • No significant difference was in steps per day among the three CKD categories, CKD stage 1–4, dialysis, and post-transplant • Higher steps per day was related to higher 6-min walking distance; steps increased per 100 and 6MWT increased 1.5 (1.1–2.0), *P* < 0.001 (adjusted for age, sex, and CKD categories) • Steps per day were negatively related to BMI per kg/m^2^. −182 (95% CI −356 to −8), *P* = 0.04 • The increase of steps per day increased physical functioning score on PedsQL 0.15 (95% CI 0.04–0.26), *P* = 0.009 • The steps per day were related to the increase of haemoglobin per dg/dl 1039 (95% CI 539–1539), *P* < 0.001 (multivariable regression) Percentage meeting the recommendation 90% of the males and 95% of the females were below the recommended levels of 15,000 steps per day for males and 12,000 steps per day for female
Hudson, 2024 [13]	Cross-sectional study (using a cohort study)	Across all stages of CKD, including dialysis and kidney transplantation	New Zealand and Australia	To quantify the number of missed school days and participation in sports in children with CKD and to determine the association between stage of CKD, sports participation, and school absences	*N* = 377, 12.2 ± 3.8, M = 233 (61.8), F = 144 (38.2)	The levels of PA (sports) • Overall, 62% out of 377 participants played sport. The sport participation was 21 (48.8%) out of 43 children on dialysis, 48 (36%) out of 135 with transplants, 23 (25%) out of 92 children with CKD 3–5, and 28 (27%) out of 107 children with CKD 1–2. 7% of children on dialysis played two or more types of sports • Compared with CKD 1–2, the incidence rate ratio (IRR) (95% CI) for sports participation among children with CKD 3–5, dialysis, and transplant were 0.84 (0.64–1.09), 0.59 (0.39–0.90), and 0.75 (0.58–0.96), respectively, adjusted for socioeconomic status and ethnicity (*P* = 0.04) • The most common sports: swimming (17%), soccer (17%), football/rugby (12%), dance (9%), and basketball (8%) among 377 participants • Participation in football/rugby was higher among children with CKD 1–2 (52%) and CKD 3–5 (28%) than children treated with dialysis (7%) and with kidney transplants (13%)
Clark, 2016 [14]	Cross-sectional study (using a cohort data)	CKD 1–4	North America	To examine self-reported physical activity and screen time in adolescents with CKD 1–3 in comparison with healthy peers as well as to characterise changes in physical activity and screen time with progression of CKD To characterise changes in physical activity and screen time with progression of CKD	*N* = 224, median (IQR) 15 (12, 16), M = 134 (59.8), F = 90 (40.2)	Levels of PA (general PA, exercise, sports) • 30 (13.4%) children met PA 2008 recommendation, lower than 24.8% of 510 healthy controls (*P* < 0.001). No difference was between females and males meeting PA recommendation. 13.3% VS 13.4%. In the subgroup of 136 children on CKD, 20 (14.7%) met PA recommendation, higher than a year later (7.5%, *P* = 0.03) • The median number of days with 60/20/30 min of exercise per week was 3 (0, 5), 3 (1, 5), and 2 (1, 5). In the subgroup of 136 children on CKD, the number of days engaged in various minutes of exercise per week remained at a similar level a year later (*P* = 0.1, *P* = 0.61, *P* = 0.92) • Increased height percentile was associated with the number of days in the past 7 engaged in 60 min of physical activity (*β* coefficient = 0.01, *P* = 0.03) • Obesity was associated with the number of days in the past 7 engaged in 60 min of PA (*β* coefficient = −0.95, *P* = 0.01) and the number of days in the past 7 engaged in 20 min of PA (*β* coefficient = −0.75, *P* = 0.04) • The median number of sports teams played on in the past 12 months was 1 (0, 2). Seventy-eight (57%) CKD reported sports team participation, higher than a year later, 65 (48%), *P* = 0.02 Settings of physical activity (physical education) • Median days of participating in PE per week was 2 (0, 5). Median (IQR) number of minutes exercising in the average physical education class was 4 (3, 5). In the subgroup of 136 children on CKD, the number of minutes exercising/playing in PE sports in gym class remained similar a year after (*P* = 0.51) Inactivity (screen time) • 4 (1.8%) children met screen time recommendations lower than the healthy controls 61 (27.0%) (*P* < 0.001) * limiting the total amount of entertainment screen time to ≤2 h per day for children and adolescents 2 years. Both female and male children were lower than those in healthy controls (*P* < 0.001) • Lower eGFR (*β* coefficient = −0.02, *P* = 0.03) and obesity (*β* coefficient = 1.04, *P* = 0.04) were independently associated with greater screen time • Median (IQR) number of hours spent watching TV on average school day, playing video games, and total screen time on an average school day were 4 (3, 5), 3 (2, 4), and 7 (5, 9), which were similar compared with 1 year later
Muhaisen, 2012 [15]	Cross-sectional study	CKD 2–5	Palestine	To investigate the association between various risk factors of CVD and different stages of CKD in children patients without kidney replacement therapy in Gaza strip	CKD 2~5 non-kidney replacement therapy *n* = 60, 12 (20) 1–4 y, 20 (33.3) 5–8 y, 28 (46.7) 9–12 y, M = 40 (66.7), F = 20 (33.3) Healthy controls n= 52, 11 (21.2) 1–4 y, 22 (43.3) 5–8 y, 19 (36.5) 9–12 y, M = 28 (53.8), F = 20 (46.2)	Intensity of PA (moderate to vigorous physical activity, inactivity) Moderate to vigorous physical activity • 30 (50%) were ‘inactive’, with no PA beyond baseline activities of daily living or less than 150 min/week of moderate-intensity PA. 30 (50%) were ‘active’, with ≥150 min/week of moderate to vigorous intensity activity Inactivity • Frequency of physical inactivity among patients was twofold higher than 52 healthy controls (50% VS 25%) • The prevalence of physical inactivity was also increased as CKD developed from 3 to 5
Foster, 2011 [16]	Cross-sectional study	CKD 2–4 and dialysis	USA	To characterise whole body and regional lean muscle and fat mass relative to body size and pubertal stage in children and adolescents with CKD and to identify correlates of lean mass deficit in CKD	*n* = 143, CKD 2~3, median (IQR) 13.2 (10.8, 17.3), CKD 4~5 14.9 (11.0, 16.6), dialysis 14.5 (12.2, 17.6) M = 87 (60.8), F = 56 (39.2) Healthy controls n = 401 11.6 (8.5, 14.9) M = 467 (48.7), F = 499 (52.1)^a^	The levels of PA (general PA) and settings of PA (leisure time after school) • Median (IQR) hours of leisure time after school were 1.91 (1.30, 2.87) in 64 children with CKD 2–3, 1.41 (0.53, 2.67) in 45 children with CKD 4–5, and 1.52 (0.78, 2.52) in 34 children on dialysis. Compared with median hours of PA of 1.81 (1.17, 2.94) in 401 healthy controls, the differences were not significant in CKD 2–3 (*P* = 0.3) and dialysis (*P* = 0.2), and higher than children with CKD 4–5 (*P* = 0.03) • Per 1 h/d increase in PA contributed to an increase in lean mass z scores; 0.05 [0.001, 0.10] increase in whole body (*P* = 0.06), 0.07 [0.02, 0.12] increase in trunk (*P* = 0.008), and 0.04 [0.004, 0.09] increase in leg (*P* = 0.07) • Per 1 h/d increase in PA contributed to a decrease in fat mass z scores, 0.05 [0.11, 0.008] decrease in whole body (*P* = 0.09), 0.05 [0.11, 0.01] decrease in trunk (*P* = 0.1), and 0.06 [0.13, 0.002] decrease in leg (*P* = 0.04)
Anderson, 2024 [17]	Cross-sectional study	CKD and dialysis	UK	To explore a practical approach to guide prescribed nutritional interventions, using measurements of total energy expenditure (TEE), physical activity energy expenditure (PAEE), and their relationship to kidney function	*n *= 18, 11.9 ± 3.4, M = 9 (50%) Healthy controls *n* = 20, 11.8 ± 3.3	PA levels (general PA) • PAEE with or without adjustments for age, gender, weight, and height did not differ between the groups and was not related to eGFR. TEE ranged from 1927 ± 91 to 2330 ± 73 kcal/d; 95 ± 5 to 109 ± 5% estimated average requirement (EAR), physical activity level (PAL) 1.52 ± 0.01 to 1.71 ± 0.17, and PAEE 24 to 34% EAR
Painter, 2007 [18]	Cross-sectional study (with longitudinal subgroup)	KRT	USA	To compare laboratory measures of fitness among children with dialysis and transplants To measure fitness using a standard field test	*N* = 40 (longitudinal sample = 9), 15.9 ± 2.45, M = 15 (37.5), F = 25 (62.5)	Level of PA(general PA) • Physical activity participation dropped following transplantation in the 9 pre-/post-transplant participants (3 months after transplantation) (no number/figure presented) Settings of PA(physical education, leisure time after school) • PE participation in 25 with kidney transplants was 48% and 20% in 15 children on dialysis • <10% non-school time were spent on participating in physical activity. This number was 9.6% in transplant recipients and 7% in dialysis participants Intensity of PA(average intensity, highest intensity) • In transplant recipients, average METs over 3 days (non-school time) was 1.8 ± 0.63; the highest level of activity was 3.5 ± 2.3 • In dialysis participants, average METs over 3 days (non-school time) was 1.9 ± 1.1. The highest level of activity was 3.9 ± 2.9 • There were positive correlations between resting diastolic blood pressure and the average activity METs and the maximal activity METs (*r* = 0.521; *r* = 0.507; *P* < 0.01) Barriers (physical education) • The reasons for not participating in PE was not required or home-schooled. One in dialysis and two in transplant cited health for the reason for non-participation
Pattaragarn, 2004 [19]	Cross-sectional study	Dialysis	USA	To evaluate the correlation between exercise capacity and haemoglobin in paediatric patients with kidney failure treated with automated peritoneal dialysis and haemodialysis	*n* = 14, 14.5 ± 2.5 M = 9 (64.3), F = 5 (35.7) Healthy Controls *n*= 8, 14.4 ± 1.5, M = 4 (50), F = 4 (50)	Overall activeness (PAQ) Mean scores of PAQ in children with dialysis and 8 age-matched healthy controls were 2.5 ± 0.7 and 2.8 ± 0.6. Both are moderate level of PA, with no significant difference in the groups’ activity score (*P* > 0.05)
Dandamudi, 2023 [20]	Cross-sectional study	HD	USA	To assess paediatric nephrologists’ philosophies and practices regarding physical activity restrictions placed on HD patients with different vascular access types to identify trends that require further study	Paediatric nephrologists *N* = 35,	Barriers and facilitators (sports) Physicians’ recommendation For children on HD, physicians did not allow participation in martial arts nor swimming. Physicians allowed racquet sports, soccer, basketball, baseball, skiing, and gymnastics Sports such as field hockey and ice hockey were mixed with responses. Over 69% of physicians did not allow weight training whilst 90% did not provide a response for the reason. The rationale for activity limitations was mainly personal experience, what has been taught, and the standard of care at centre
El-Gamasy, 2017 [21]	Cross-sectional study	HD	Saudi Arabia	To clarify biodemographic characteristics, common complaints, and physical and psychosocial status of children with kidney failure under regular maintenance haemodialysis	*N* = 30, 14.73 ± 4.56, M = 13 (43.3), F = 17 (56.7)	Level of PA (exercise) Seven (23.3%) haemodialysis participants practise exercise regularly Settings of PA Among 7 children who exercised regularly, 7 (23.3%) and 3 exercised at home and 4 exercised at street no one at school
Souza, 2019 [22]	Cross-sectional study	HD	Brazil	To identify the occupations impacted by chronic kidney disease in the child and adolescent population	*N* = 21, 13.4 ± 2.8 M = 16 (76.2), F = 5 (23.8)	Twelve (57.1%) out of 21 haemodialysis children identified playing soccer were impaired by the CKD and haemodialysis. And they rated the importance of playing soccer as 9 ± 2.4 out of 10. Three (14.3%) children reported swimming being affected with importance scores 8.7 ± 2.3 out of 10. One (4.8%) child identified jumping rope and training at the gym were affected with importance scores 6 and 3, respectively
Ladfors, 2011 [23]	Cross-sectional study (using a cohort data)	Transplant	Sweden	To examine the exercise capacity after kidney transplantation compared to matched controls To relate the results to physical activity and other parameters To follow exercise capacity over time	*n* = 38, median (range) 13.6 (7.7–18.0), M = 20 (52.6), F = 18 (47.4) Healthy controls *n* = 17, median (range) 11.7 (7.3–18.6), M = 10 (58.8), F = 7 (41.2)	Level of PA(general PA) 19 out 38 (50%) transplant recipients reported no regular physical activity compared to 0 out of 17 healthy controls reported no regular PA (*P* < 0.0001) Intensity of PA(moderate to vigorous physical activity) Moderate to vigorous physical activity 1–2 h/week: 12 out of 38 (31.6%) transplant recipients VS 8 out of 17 (47.1%) healthy controls moderate to vigorous physical activity ≥ 3 h/week: 7 out of 38 (18.4%) transplant recipients VS 9 out of 17 (52.9%) healthy controls *P* = 0.0003
Clark, 2012 [24]	Cross-sectional study	Transplant	Canada	To quantify PA levels with accelerometers in a sample of children who had undergone kidney transplantation	*N* = 16, 13.1 ± 4.0, M = 7 (43.8), F = 9 (56.2)	Intensity of PA (moderate to vigorous physical activity, steps per day, inactivity) Moderate to vigorous physical activity • Mean time of moderate to vigorous PA measured by accelerometery in 14 patients was 29.3 ± 24.6 min/day • 3 (19%) accumulated ≥1 h moderate to vigorous PA 1 out of the 3 days • 37% of time on light activity and 4.5% of time on moderate to vigorous PA Steps per day • Mean steps per day was 7494.8 ± 4428.8 and ranged from 117 to 19,646.7 • 14 out of 16 below the recommended daily step Inactivity 58.5% of their time was spent in sedentary activity and mean sedentary time was 476.1 ± 174.3 min/day
Daudet G, 2005 [25]	Cross-sectional study	Transplant	France	Evaluation of physical capacity in kidney transplanted children for sport practice adequacy	*N* = 32, 13.9 ± 3.4, M = 13 (40.6), F = 19 (59.4)	Settings of PA(physical education) • 24 out of 32 (75%) engaged in the physical education class
Dobbels, 2010 [26]	Cross-sectional study	Transplant	Belgium	To assess HRQOL, depressive symptoms, side effect experience, and treatment adherence in a sample of adolescent kidney transplant patients, using self-report and parent-report	*N* = 23, median (IQR)15 (14, 17), M = 16 (69.6), F = 7 (30.4)	The levels of PA (sports) and frequency and duration of PA • 11 out of 23 (47.8%) regularly participated in sports • 4 out of 23 (17.4%) everyday; 1 out of 23 (4.3%) 4–5 times a week; 4 out of 23 (17.4%) 2–3 times a week; 2 out of 23 (8.6%) weekly basis
Hamiwka, 2009 [27]	Cross-sectional study	Transplant	Canada	To determine lifestyle PA levels in a group of kidney transplant recipients compared with healthy controls To explore potential physiological correlates to their PA levels To determine HRQOL and psychological associations of PA in kidney transplant recipients	Transplant *n* = 20, 14.3 ± 3.2, M = 8 (40), F = 12 (60) Healthy controls *n* = 33, 14.0 ± 1.6, M = 14 (42.4), F = 19 (57.6)	Level of PA (general PA, sports, frequency and duration) • Mean minutes for physical activity were 107.3 ± 126.8 min per week, lower than 488.2 ± 640.7 min in healthy controls (*P* = 0.005) • No relationship was found in BMI and the total exercise time. Overall HRQOL measured by PedsQL 4.0 was not related with total exercise minutes (no detailed number provided) • Soccer and badminton were reported less frequently by transplant recipients whereas activities like tag, floor hockey, and baseball were reported more commonly by the transplant recipients • Transplant and healthy controls have similar frequency performed ≥3 times per week Settings of PA (physical education) • 12 out of 20 (60%) of transplant recipients and 29 out of 33 (87%) of healthy controls were very active in school physical education classes (*P* = 0.084) Steps per day • Mean steps per day in children with transplants were 9282 ± 4666, similar with healthy controls of steps of 11,449 ± 4638, *P* = 0.124 • 10% of transplant recipients and 25% of healthy controls met the sex cutoffs for steps/day, *P* > 0.05 • No relationship was found in BMI and the steps per day (no detailed number provided) • DTPA-GFR, systolic BP, and diastolic BP were not related with steps per day or mean PAQ summary score (no number provided) Overall activeness (PAQ) • Mean PAQ scores were 2.8 ± 0.8 in transplant recipients and 3.1 ± 0.7 in healthy controls with similar age and gender (*P* = 0.180) • The PAQ score was negatively correlated with age (*r* = −0.46, *P* = 0.04) in the transplant recipients • Higher PAQ scores, the lower waist circumference (*r* = −0.42, *P* = 0.036) • No relationship was found in BMI and the PAQ scores (no detailed number provided) • Higher PAQ activity scores were significantly correlated with perceptions of physical conditioning (*r* = 0.71, *P* = 0.001), sport competence (*r* = 0.64, *P* = 0.003), and body attractiveness (*r* = 0.55, *P* = 0.019)
Heffernan, 1998 [28]	Cross-sectional study	Transplant	Britain and Ireland	To understand the opinion regarding which sports are acceptable and which sports are not permissible for children following kidney transplantation	Paediatric nephrology units *N* = 17	Barriers and facilitators Physicians’ recommendations For children with transplants, physicians agreed on avoiding martial arts and agreed on participating in swimming, tennis, badminton, cycling, field hockey, sailing, canoeing, netball, cricket, track athletics, golf, skiing, and rowing, and had split opinions on horse-riding, fencing, hurling, avoided Gaelic football, long high jump, and individual units were opposed to soccer, basketball, volleyball, and long jump
Kohlmeier, 2024 [29]	Cross-sectional study	Transplant	Germany	To assess frequency, duration, intensity, and setting of PA in kidney transplant recipients To analyse PA’s potential impact on cardiovascular risk factors and subclinical cardiovascular target organ damage	*N* = 48, 13.5 ± 4.2, M = 26 (54.2), F = 22 (45.8)	Levels of PA(sports) • Mean minutes of sports activities per week was 129 (95% CI 97, 162) min, which was lower than the healthy German KiGGS cohort (242 min/week; 95% CI, 230–253) (box diagram provided) • Transplant patients also engaged fewer minutes of sports activities across thesportssettings (school, clubs, and other unorganised sports) and age groups. The biggest discrepancy was in sports clubs. And there was no overlap for the total amount of sport between our study population and the KiGGS cohort for all age groups (box diagram provided) Settings of PA(physical education) • Twenty-six percent of total time of moderate to vigorous activity per day (489 ± 435 min) was spent on sport activities mainly performed during school (51% sportactivitiesin school, 26% sport activities in sport clubs, 23% other unorganised sport activities) Intensity of PA(moderate to vigorous PA, vigorous PA) • Mean minutes of moderate to vigorous PA per week was 489 ± 435 min measured by ‘Motorik–Modul’ PA questionnaire. 88% of it was moderate PA (431 ± 430 min) and 12% was vigorous PA (58 ± 95 min) • 23 (48%) met the recommendation of 60 min moderate to vigorous PA/day (420 min/week) • 74% moderate to vigorous PA was performed as everyday activities with mainly moderate intensity (moderate PA 356 ± 421 min vs. vigorous PA 7 ± 37 min) • 26 (54%) did not practise vigorous PA. Vigorous PA was mostly practised during sport activities • Moderate to vigorous PA o Associated with a higher mitral annular e′ (*P* = 0.01) and lower E/e′ ratio (*P* = 0.01), indicating better left ventricular compliance and less left ventricular filling pressures o Associated with a decrease of mitral A velocity, A-wave (indicating a larger contribution of atrial contraction to left ventricular filling) (*P* = 0.02) o Had no significant effect on rest heart rate (*P* = 0.18) o Had no significant effect on either SBPz (*P* = 0.89) o An additional 22 h per week of MVPA was related to one component less of post-transplantation metabolic syndrome (*P* = 0.04) o Higher eGFR 88.1 ± 43.2 ml/min/1.73 m^2^ was in the group with adequate moderate to vigorous PA (≥420 min per week) compared with 66.4 ± 28.4 ml/min/1.73 m^2^ in the group inadequate moderate to vigorous PA (*P* = 0.04) • 60 min more vigorous PA per week predicted o A decrease of a rest heart rate by 3 beats/min, adjusted for sex, age, and eGFR (*P* = 0.01) o A decrease of 0.18 in SBP z score (*P* = 0.02) Steps per day From 23 participants, the mean steps per day were 7317 ± 3406 Inactivity 14 (29%) of children with transplants indicated an inactive lifestyle <120 min/week of moderate to vigorous intensity PA
Krasnoff, 2006 [30]	Cross-sectional study	Transplant	USA	To measure and compare physical fitness using both laboratory measures and FITNESSGRAM and to assess physical activity participation in paediatric kidney transplant and liver transplant recipients	*N* = 25, 15.7 ± 2.3, M = 16 (64), F = 9 (36)	Settings of PA(physical education, leisure time after school) • 48% of 25 children on transplants participated in physical education (3 days/week for 45 min) • Only 9.6–10% after school time was spent on PA. The majority of time was spent in sedentary activities (television, computer) and self-care tasks (eating, bathing) Intensity of PA(average intensity, highest intensity) • The average MET level of activity over the 3 days was 1.8 METs. The highest level of activity reported averaged 4 METs
Lubrano, 2016 [31]	Cross-sectional study	Transplant	Italy	To compare cardiovascular fitness between children with renal transplantation and children with congenital solitary functioning kidney taking account of weekly sport activity	*n* = 20, 13.3 ± 2.7, NK Children with congenital solitary functioning kidney: *n* = 30, 13.8 ± 5.0, NK	Levels of physical activity Percentage of individuals participating in exercise • 10 out of 20 (50%) children with transplants reported to participating in exercise over 3 h/week. 10 (50%) reported less than 3 h of exercise per week • Those who exercise more than 3 h/week had higher VO_2_max/kg; ml/min/kg measured by a maximal incremental exercise on a treadmill (Bruce protocol) than those who exercising less than 3 h/week, 28.99 ± 1.15 VS 23.22 ± 1.23; *P* < 0.003
Lubrano, 2012 [32]	Cross-sectional study	Transplant	Italy	To test relationship of level of cardiorespiratory fitness in a number of children after successful kidney transplantation, and the amount of regular physical exercise and comparing the findings with matching healthy controls	*N* = 16, 16.0 ± 2.5, M = 14 (87.5), F = 2 (12.5)	Levels of PA (exercise) • 10 (62.5%) reported less than 3 h of exercise per week and 6 (37.5%) reported more than 3 h of exercise per week • Those who exercised more than 3 h per week had o A higher VO_2_max/kg (ml/min/kg) 29.59 ± 4.28, compared to 24.79 ± 1.99 in children exercising less than 3 h, *P* = 0.0048 o Similar respiratory rate (max effort during exercise) 41.48 ± 8.4, compared to 40.20 ± 6.3 in children exercising less than 3 h (*P* > 0.05) o Better left ventricular mass 42.40 ± 19.70 g/m^2^, compared to 66.83 ± 21.59 g/m^2^ in children exercising less than 3 h (*P* = 0.038) o Similar mean height (SDS) −1.86 ± 0.66, compared to −1.83 ± 1.07 in children exercising less than 3 h (*P* > 0.05) o Similar GFR 91.83 ± 23.82 mL/min/1.73 m^2^, compared to 93.1 ± 22.21 mL/min/1.73 m^2^ in children exercising less than 3 h (*P* > 0.05)
Matteucci, 1996 [33]	Cross-sectional study	Transplant	Italy	To investigate cardiovascular and respiratory conditions at rest and cardiovascular response in exercise stress testing on a treadmill in a group of young kidney transplant patients to evaluate the physical activity habits of these patients by means of a questionnaire	*N* = 10, median (range), 15.1 (6.1, 21.5), M = 7 (70), F = 3 (30)	Level of PA(exercise, frequency and duration) • Four out of 10 (40%) participating in exercise in the last 1 year, with training 1–3 days per week in the last 6 months, with 30–60 min each training session
Tangeraas, 2010 [34]	Cross-sectional study		Norway	To assess the level of fitness and daily PA after transplantation compared with healthy children To evaluate the level of cardiorespiratory fitness in relation to traditional cardiovascular risk factors	*N* = 22, median (range) 14.5 (8.6–18.5), M = 16 (72.7), F = 6 (27.2)	Intensity of PA(average intensity, moderate to vigorous PA) • Median METs level per 24 h was 9.6, ranging from 7 to 15.5 • 19 (86%) reported <60 min of daily moderate to vigorous PA, with median 26 (0–81) min • Minutes of moderate to vigorous PA per week, PA recalled by questionnaire o Was related to VO_2_max measured by treadmill (*r* = 0.49, *P* = 0.03) o Was associated with children with 1 metabolic risk factor (median 36 min) or less compared with children with 2 or 3 risk factors (23 min) (hypertension, overweight, and glucose intolerance) (*P* = 0.02)
Tranchita, 2023 [35]	Cross-sectional study	Transplant	Switzerland	To investigate the level of PA after liver or kidney transplant	*N* = 15, NK^b^, NK^b^	Overall activeness (PAQ) The mean score of PAQ was 2.17 ± 0.68. No respondent stated that they ran and played hard most of the time, or at recess and at the lunch time
Tranchita, 2023 [36]	Cross-sectional study	Transplant	Italy	To compare the lifestyle and analyse the practice of physical exercise between young patients with transplants and healthy controls	Transplant *n* = 11^c^, 12.86 ± 3.5, M = 18 (41.9), F = 25 (58.1)^c^ Healthy controls *n*= 61, 12.75 ± 2.8, M = 35 (57.3), F = 26 (42.6)	Overall activeness(PAQ) Mean score of PAQ was 2.16 ± 0.75 in children and adolescents. Mean scores for questions 2 and 8 were 2.82 ± 1.25 and 2.27 ± 1.19, respectively
Weigmann-Fasbender, 2020 [37]	Cross-sectional study	Transplant	Germany	To investigate the cardiorespiratory fitness, muscle strength, motor coordination, daily physical activity, and health-related quality of life of transplantation in comparison with controls	Transplant *n* = 20, 13.5 ± 3.4, M = 16 (80), F = 4 (20) Healthy controls *n* = 33, 13.1 ± 3.2, M = 25 (75.8), F = 8 (24.2)	Steps per day • Average steps per day were 7007 steps measured by SenseWear armband monitor, lower than 10,305 steps on average in healthy controls (*P* = 0.001) • Children with transplant were divided into three groups: (a) no physical activity besides physical education, (b) low (or no regular) physical activity, and (c) moderate physical activity, that is, more than once per week o There were tendencies for VO_2_max and peak power output to be increased in subjects with a higher physical activity level in both groups (detailed number not provided) o There was a tendency for a greater handgrip strength in moderately active subjects (detailed number not provided) o QoL measured by PedsQL ^TM^ version 4.0 Generic Core scales was lower in participants with no regular PA per week (detailed number not provided)
Welch, 1999 [38]	Cross-sectional study	Transplant	USA	To address safety of the climbing harness for adolescents with kidney transplants	*N* = 10, median (range) 20 (15, 23), M = 9 (90), F = 1 (10)	Barriers and facilitators All devices (climbing harnesses) came into contact with all transplants. Sports requiring the use of climbing harnesses (for instance, rock climbing, rappelling, and challenge course participation) may be unsafe for recipients of kidney transplants
Wolf, 2016 [39]	Cross-sectional study	Transplant	USA	To evaluate the level of physical activity and grip strength among children and young adults with kidney transplants To describe attitudes about exercise and exercise counselling regarding sport limitations and allograft injury	*N* = 101, 14.2 ± 3.9, M = 67 (66.3), F = 34 (33.7)	Levels of physical activity(general PA and sports) • 67% participants reported an increase in PA level after transplant; 13% reported less in activity and 20% reported no change in activity despite that limits on activity were more likely after transplant (68%) than before transplant (49%) • 31% of children and young people with kidney transplants played organised sports during the previous year. The most played sports were basketball (31%), baseball (10%), soccer (9%), and cheerleading (8%) Overall activeness(PAQ scores) • The median score of PAQ was 2.2 (IQR 1.6, 2.6). In 59 children with the mean age 11.5 ± 2.5, median PAQ score was 2.4 (IQR 1.9, 2.9); lower than healthy children in USA with a median PAQ of 3.2 (*P* < 0.001). In 42 adolescents with the mean age 18 ± 1.9, median PAQ score was 1.8 (IQR 1.3, 2.3); lower than healthy adolescents in USA with a median PAQ of 2.8 (*P* < 0.001) • PAQ scores were higher in elementary/middle schoolers compared with high school/college. The difference was −0.48 (−0.76, 0.21), *P* < 0.001. The older age (year) was the predictor of the PAQ scores with the estimate −0.081 (−0.11, −0.048), *P* = 0.0006
Palma, 2023 [40]	Retrospective study	CKD 1–4	Italy	To evaluate the incidence of CKD progression within 6 months after the end of the first Italian lockdown and the factors that could have contributed to it	*N* = 21, 10.6 ± 4.8, M = 16 (76.2), F = 5 (23.8)	Levels of PA (general PA) • Mean hours of PA per week were 7.9 ± 4.5 pre lockdown and 3.7 ± 4.1 after lockdown • Before and after lockdown, mean decrease of physical activity hours per week was −4.05 ± 6.0 among 11 children with worsening CKD progression and was −4.35 ± 4.19 among 10 children without worsening CKD progression. The difference was not significant, *P* = 0.90 Inactivity • Mean hours of screen time was 10.1 ± 5.1 h per week pre lockdown and 26.0 ± 10.5 after lockdown Before and after lockdown, the increase of screen time was 17.82 ± 8.49 h per week in children with worsening CKD progression and was 13.85 ± 8.53 h per week in children without worsening CKD progression, *P* = 0.30
Ahmed, 2022 [41]	Retrospective study	Transplant	Canada	To assess height adjusted lean body mass (ht-LBM) levels in paediatric kidney transplant recipients and test its association with variables, including physical activity	*N* = 17, median (IQR) 16.0 (12.3, 17.7)^d^, M = 26 (56.5), F = 20 (43.5)^d^	Overall activeness (PAQ) The mean score of PAQ was 2.4 ± 0.9 Ht-LBM levels were related to PAQ scores (*r* = 0.49, *P* = 0.047). Ht-LBM was related to PAQ scores (*β* = 1.66, *P* = 0.02) after adjusting for the age at transplant
Lui, 2021 [42]	Retrospective study	Transplant	Canada	To assess whether overall HRQOL was associated with the self-reported level of paediatric transplant patients To determine whether this association was independent of indicators	*N* = 27, median (IQR) 15 (12, 18), M = 17 (63.0), F = 10 (37.0)	Overall activeness (PAQ) Median IQR of PAQ was 2.4 (1.4–3.0)
Lui, 2020 [43]	Retrospective study	Transplant	Canada	To assess the PA levels among paediatric solid organ transplant patients To identify factors that may play a contributing role to their level of PA	*N* = 29, median (IQR) 15.6 (12.8~17.8), M = 17 (58.6), F = 12 (41.4)	Overall activeness (PAQ) Median IQR of PAQ was 2.1 (1.6–2.8)
Wong, 2025 [44]	Retrospective chart review	CKD and transplantation (excluding dialysis)	USA	To evaluate: how often patient PA was documented in the medical record, how often the provider recommended PA, and the impact of selected demographics, indexes of disease severity, and prior sport participation on the likelihood of a PA recommendation	*N* = 404, mean (SD) 12.4 ± 4.0, M = 247 (61.4)	Barriers and facilitators (clinicians’ recommendation) In electronic medical record, 239 (59.2%) had documented PA participation, and 119 (29.6%) had a documented PA recommendation in their chart. PA recommendations were more likely in older patients (odds ratio [OR]: 1.08; 95% CI, 1.03–1.13), those with higher body mass index z score (OR: 1.45; 95% CI, 0.96–1.51), higher height z score (OR: 1.55; 95% CI, 1.31–1.82), higher eGFR (OR: 1.01; 95% CI, 1.01–1.02), sport participation (OR: 2.47; 95% CI, 1.46–4.19), and less likely for patients with higher motor score (OR: 0.6; 95% CI, 0.41–0.87; all *P* < 0.01). Adjusted analysis maintained significant associations except sports participation and motor score
Denburg, 2016 [45]	Prospective cohort study	CKD 1–5	North America	To determine the gender-specific incidence of fracture in CKiD (a cohort of children with CKD 1–3) and to identify risk factors for fracture in this high-risk population	*N* = 290, median (IQR) 11.0 (7.4, 14.5)^e^ M = 326 (60.7), F = 211 (39.3)^e^	Level of physical activity (sports and height) • Height z score was positively related to the number of team sports in the prior 12 months (Spearman’s *R* = 0.14–0.23, *P* < 0.03) • The median height of the participants who played >1 team sport was closer to the 50th percentile as compared with those who participated in ≤1 team sport for whom the median height was around the 25th percentile Fracture • Participation in >1 sport was associated with a nearly fivefold higher risk of facture (HR, 4.87; 95% CI, 2.21–10.75; *P* < 0.001). Participation in ≥1 team sport was associated with a HR of 2.35 (95% CI, 1.01–5.47; *P* = 0.047)
Hogan, 2020 [46]	Prospective cohort study	CKD 1–5	North America	To quantify the association between grip strength and kidney function and to explore factors associated with grip strength in children and adolescents with CKD 1–3	*N* = 388^f^, age: median IQR 14 (11, 17) (*n* = 411) M = 253 (62.5) F = 158 (38.5)	Levels of physical activity (exercise and sports) • The median number of the days with more than 60 min of exercise per week was 5 (3, 8) • Grip strength z score increases with the number of days with more than 60 min of exercise; the mean effect 0.06 95% CI 0.02, *P* = 0.008 • Median number of sports teams played for in the 12 months: 0 (0, 2) Intensity of PA(moderate to vigorous PA, vigorous PA) • Percentage of patients with vigorous activity for more than 10 min was 353 (54%). Median (IQR) number of days with vigorous activity was 1 (0, 4) • Grip strength z score increases with the number of days with vigorous activity; the mean effect 0.06 95% CI 0.02, *P* = 0.008. Grip strength z score increases with the practice of vigorous activity more than 10 min; the mean effect 0.36 95% CI 0.02, *P* = 0.0002
Oberdhan 2022 [47]	Qualitative study	ADPKD	13 countries	To describe burden of disease in adolescents with ADPKD, including the symptoms, impact on daily life, and emotional and social issues and concerns. To report qualitative ﬁndings on the burden of disease in adolescents.	*N *= 33,14.6* ± *1.8,M *= *19(57.6)F *= *14(42.4)	ExperienceFeeling constraints by CKD and change of physical activity participation by avoiding sports.Barriers 10 (30.3%) also reported not doing sports or avoiding sports based on the advice of their doctors or parents. Girls tended to report a higher rate of avoiding sports, female 6 (42.9%) VS male 4 (21.1%) in reporting can’t do sports or avoiding sports
Lee, 2018 [48]	Qualitative study	CKD 1–3	Korea	To develop a substantive theory on self-management conducted by the adolescents with chronic kidney disease from their lived experience	*N* = 13, range 14.0–19.0, M = 6 (46.1), F = 7 (53.8)	Theme core category: overcoming unstable self-control to integrate illness as part of life, factors such as catching a cold, engaging in strenuous activities, or experiencing stress can exacerbate or trigger the recurrence of kidney disease symptoms. ‘A little bit, if I walk for a long time, participate in sports events, or exercise for a long time, my body doesn’t follow. When I was hospitalized this time, I had traveled abroad the previous week, walked a lot, and maybe it was because of that, or maybe it started swelling a bit before that, but I was already swollen, and I felt more tired after coming back from abroad, so it felt like it happened faster.’ (Participant 3) Themes action/interaction strategies, active coping Participants learned through trial and error during their prolonged illness to control their actions and actively manage their illness whilst striving for health-related goals. Participants participated only in performance evaluations during school physical activities and adjusted their physical activity within a reasonable range according to their physical condition ‘In middle school, I didn’t participate in soccer or baseball, and I only played badminton or table tennis because there was a performance evaluation in high school.’ (Participant 6)
Muhammad, 2016 [49]	Qualitative study	CKD 4–5	UK	To identify coping strategies in people with CKD 4–5 and healthy group aged 12–18 years	*N* = 7, mean (range) 13.6 (12.0–16.0), M = 4 (57.1), F = 3 (42.9)	Barriers and facilitators Exercise Demonstrating involvement in physical activity is an important strategy. One young person says ‘I’m not great at sport but I enjoy doing it’ (Participant 1:3). Another said ‘I’m not that much of a sporty person’ (Participant 1:2); another comment was ‘She’s done abseiling, canoeing – everything’ (Participant 1:4). Being active is important. One young person said, ‘I just play football with friends and stuff’ (Participant 1:1). Another comment was ‘I think any activities can sort-of takes my mind off it (health related concerns); football, running’ (Participant 1:5) and ‘I like swimming as well but I can’t go swimming’ (Participant 1:6) Pastimes On pastimes, young people from group one had varied comments including ‘Football and FIFA’ (Participant 1:5), ‘I like playing football; I like doing lots of sports and stuff’ (Participant 1:7), ‘I did like running but I can’t do it now’ (Participant 1:6), ‘When it’s sunny and the kids come round, we’ve got a trampoline in our back garden’ (Participant 1:/P4/P5 on behalf of Participant 1:4), and ‘I like playing football’ (Participant 1:1)
Wu, 2025 [50]	Qualitative study	Across all stages of CKD, including dialysis and kidney transplantation	14 countries	To describe the aspects of growth impairment that are most impactful from the perspectives of children with CKD, their parents, and health professionals from qualitative data concerning growth from the standardised outcomes in nephrology-children and adolescents (SONG-Kids) initiative	*N* (children) = 120 aged 8–21 years *N* (parent) = 250 *N* (healthcare professionals) = 445	Barriers and facilitators Constrained life participation and enjoyment (excluded from sports or competing with unfair disadvantage) ‘They say, in volleyball and basketball, that I can’t play because I am too short.’ ‘He can’t mess around with the cousins that are of the same age and my youngest son who’s the same age. It’s hard because he is so small he’s going to get hurt easier, and its upsetting to me because he wants to be there.’ ‘At school events like sports events, sometimes when I am playing a game I am always behind, running behind late to classes.’
de Carvalho Lira, 2005 [51]	Qualitative study	Transplant	Brazil	To determine the physical, social, and emotional alterations in the life of a post-kidney transplant adolescent	*N* = 12, mean (range) 15.8 (12.0–20.0), M = 6 (50), F = 6 (50)	Barriers and facilitators Theme physical alterations in the life of chronic renal adolescent post-transplant; regarding the physical limitations resulting from surgery, the most significant ones referred to the interruption of school studies, restrictions in social life, difficulty in exercising, expressed in the accounts: ‘I stopped studying because I was dizzy. I came to the hospital and ended up missing class.’ (A3). ‘Regarding the party, I always have a set time to come home because of me.’ (A2). ‘Sometimes I play. But I don’t play much because I could get hit in the kidney.’ (A5)
Thorsteinsdottir, 2018 [52]	Interventional study (with a historical control)	Transplant	Norway	To determine whether incremental physiotherapy and increased focus on physical activity during the post-transplant follow-up have a positive impact on cardiorespiratory fitness, traditional cardiovascular risk factor, quality of life, and mental health	Group_before *n* = 20, median (range) 13.0 (8.4~18.0), M = 13 (65), F = 7 (35) Group 2_after n = 22, median (range) 14.5 (8.6~18.5), M = 16 (72.7), F = 6 (27.2)	Hospital physiotherapy and health education Intervention: early post-transplantation physiotherapy and focus on physical activity; 1–2 week after transplantation, regular physiotherapy 2–3 times per week in the inpatient and outpatient hospital until 3 months after transferring to local hospital for follow-up. Health education was done to encourage children to exercise like their peers at each checkup Outcomes: • Cardiopulmonary fitness: median VO_2_max (range) was higher in the children receiving early stage of physiotherapy 44.3 (31.7, 58.8) VS 33.5 (18, 59) ml/kg/min, *P* = 0.031 • Quality of life: mean PedsQL child self-report total score was higher after intervention implemented 80.56 ± 12.61 compared with the score before intervention 69.10 ± 17.98, *P* = 0.003 Mental health by the strength and difficulties questionnaires: mean SDQ child self-report total scores was lower after intervention implemented 7.80 ± 5.16 compared with the score before intervention 11.58 ± 5.69 (*P* = 0.012)
Abd-Elmonem, 2019 [53]	RCT	CKD 3–4	Egypt	To explore the effects of progressive resistance exercises on quality of life and functional capacity in children with CKD	Intervention *n* = 16, 9.8 ± 1.4, M = 12 (75), F = 4 (25) Controls *n* = 16, 10.6 ± 1.3, M = 11 (68.8), F = 5 (31.2)	Traditional full body resistance exercise Intervention group received supervised progressive resistance exercises, including warmup, resisted exercise, and cool down. The training was twice a week with an interval of 48 h between sessions, each session of 60 min Control group received standard medical care with no change of daily activities Outcomes • Quality of life by Paediatric Quality of Life Inventory (PedsQL™) generic core scale (8–12 years): mean PedsQL score was 67.24 ± 3.43 in the intervention group, higher than the score of 53.81 ± 2.22 in the control group (*P* = 0.0001) • Cardiopulmonary fitness by 6MWT: 6MW distance was 522.5 ± 32.96 m in the intervention group, higher than the distance of 430 ± 25.03 m (*P* = 0.0001)
Cavusoglu, 2023 [54]	RCT	CKD 1–5	Germany	To compare the effectiveness of Nintendo Wii-based exergames and home-based fun video exercises on functional capacity, PA, muscle strength, fatigue, depression, and QoL in children with CKD stage 1–5	*N* = 16, 11.2 ± 3.5, M = 11 (68.8), F = 5 (31.2) Intervention I *n* = 8, 11.5 ± 3.5, M = 5.31 (5.43), F = 3 (37.5) Intervention II *n* = 8, 11.1 ± 3.7, M = 6 (75), F = 2 (25)	Intervention I: ehealth exercise cardio and flexibility training • Supervised Nintendo Wii-based exergaming, mixed (cardio + flexibility) stretching + breathing 5 min, 30 min of aerobics, stretching and yoga exercises with Nintendo Wii Fit, 5 min cool down exercise supervised by a paediatric physiotherapist in a clinical setting Intervention II: ehealth exercise cardio and flexibility training • Home-based fun video exercises video recording. 5 min of warmup, 30 min of aerobics, strengthening and yoga exercise, 5 min of cool down explained by a physiotherapist Outcomes: • Cardiopulmonary fitness measured by 6MWT; intervention I increased from 502.56 ± 116.70 to 585.81 ± 99.85 m, *P* = 0.012; and intervention II increased from 522.12 ± 110.92 to 609.31 ± 100.29 m, *P* = 0.012; the increase was not significantly different (*P* = 0.92) • Steps per day by pedometers in intervention I increased from 4788.12 ± 1919.61 to 6648.25 ± 2389.47 steps, *P* = 0.017; the increase was higher than the intervention II, *P* = 0.021 (the increase was from 3792.87 ± 1362.74 to 4486.12 ± 1367.08 steps, *P* = 0.012) • Muscle strength in in right and left shoulder abductor and flexor, right hip abductor, and right knee flexor improved in each group; compared with another group, the left knee flexion strength in the intervention I was higher (*P* = 0.037) • Fatigue and QoL also improved in both groups before and after. Fatigue measured by visual fatigue scale (VFS) decreased from 4.5 ± 3.4 to 1.37 ± 1.76 (*P* = 0.028) in intervention I and decreased from 3 ± 3.11 to 1.37 ± 1.84 in intervention II (*P* = 0.042). Physical health summary score reported by children improved from 58.93 ± 21.09 to 73.82 ± 18.44 in intervention I (*P* = 0.018) and from 67.4 ± 36.61 to 79.01 ± 30.65, *P* = 0.028
Feldkötter, 2021 [55]	RCT	HD	Germany	To assess the effects of exercise training during HD on dialysis efficacy	Intervention n = 23, 14.0 ± 3.4 M = 19 (82.6), F = 4 (17.4) Controls *n* = 22, 15.1 ± 2.4, M = 13 (59.1), F = 9 (40.9)	Intervention: traditional cardio exercise Supervised bicycle ergometer training during the haemodialysis for 12 weeks. At the first 30 min of HD warmup, endurance training, and cool down. The training was delivered three times a week, each session 50—70 min for 12 weeks with the total of 36 sessions Controls: none Outcomes • Single pool Kt/V at baseline in the intervention group was 1.7 (0.33) in intervention group and 1.8 (0.55) in control group. Single pool Kt/V 12 weeks after in the intervention group was 1.7. Single pool Kt/V at both timepoints and 12 weeks after were not different between two groups (*P* = 0.49, *P* = 0.91) • No significant differences were found in secondary endpoints such as PedsQL scores, heart rate per minute, change of solute removal during HD, change of solute removal in a two-compartment model, change in inflammation, C-reactive protein, nutritional status and bone metabolism, albumin, alkaline phosphatase, parathyroid hormone, fat-free mass, fat mass, and overhydration • No significant difference in the number and severity of AEs and serious adverse events (SAEs) • No bleeding or dislocation of the dialysis needles during the 1670 training sessions
Carbonera, 2020 [56]	RCT	Transplant	Brazil	To assess the efficacy of inspiratory muscle training on respiratory muscle strength, functional capacities, and pulmonary function in paediatric kidney transplant recipients	Intervention *n* = 16, 11.6 ± 3.7 M = 8 (50), F = 8 (50) Controls *n* = 15, 11.7 ± 3.6, M = 9 (52.9), F = 6 (35.3)	Intervention: resistance training loaded inspiratory muscle • Training using a respiratory booster for 7 times a week for 6 weeks. Inspiring through the device with enough force to overcome the threshold load, and then exhaling normally. The inspiratory load was fortnightly adjusted to 40% of the previously measured MIP, maintaining a respiratory frequency between 20 and 30 breaths per minute. 20 min a day and rest a 1-min interval during training, in case of fatigue Controls: placebo • Inspiratory muscle training with threshold sham, with a minimum load (9 cm H_2_O) Outcomes • Increase of 35% in the maximal inspiratory pressure predicted and 26% in the maximal expiratory pressure predicted in the intervention group were found, compared with 5 and 4% in the control group (*P* = 0.01, *P* = 0.046) • Maximal inspiratory pressure increased significantly after training in intervention group (*P* = 0.000) from 69.25 to 99.26 cmH_2_O but not in control group from 56.6 to 66.92 cmH_2_O (*P* = 0.082) • 6MWT were not statistical different between groups (number was not provided) • Pulmonary function measured by spirometry was not statistically different between groups • Haemoglobin improved in intervention group (increased from 12.17 ± 0.35 to 13.29 ± 0.42 g/dl) compared with the control group (increased from 11.96 ± 0.36 to 12.04 ± 0.44 g/dl, *P* = 0.047 considering time and group interaction). Creatinine, urea, potassium, and phosphorus levels did not show any significant changes at any time during the evaluation Facilitators to exercise training • Weekly behavioural consultation
Khalf-Allah, 2024 [57]	RCT	HD	Egypt	To investigate the effect of muscle stretching and isometric exercises on QoL of children undergoing haemodialysis	***N* = **60 aged 6–18 years HD: *n* = 30, M = 21 (70) Controls: *n* = 30, M = 18 (60)	Intervention: muscle stretching and isometric exercise. The study group received a 40-min exercise programme 3 times a week during HD for 2 months Personalisation: Children performed the exercises ‘depending on the child’s exercise tolerance’ Progression: Repetitions started at 10 per set and were ‘gradually increased to 15 repetitions’ For isometric training, contractions were held for 3–5 s Outcomes: QoL by PedsQL™ scale. Poor QoL defined as score less than 50%, 50–75% fair, and 75–100% good Children from the study group had a higher proportion of reporting good QoL compared with control group (66.7% VS 3.3%, *P* = 0.001)
Baqai, 2025 [58]	RCT (pilot)	CKD 2–4 with anaemia	USA	To evaluate whether a modest delay in iron initiation is safe and to generate preliminary data to inform future research and policy	Iron therapy group: *n* = 10, 12.8 ± 6.2, M = 3 (50%) No-iron therapy group: *n* = 11, 13.1 ± 2.8, M = 7 (70%)	Intervention: 3-month iron sulphate therapy • PA: there was a trend toward an increase of physical activity in both groups, with no significant different between groups (by 10 item paediatric proxy PA questionnaire from Patient-reported outcomes measurement information system, PROMIS) • Fatigue: there was a trend toward a reduction of fatigue in the iron therapy group, compared to no-iron therapy group (*P* = 0.2) (measured by PROMIS) • Muscle mass: did not change in either group (measured by bioelectrical impedance analysis) • Muscle strength: there was a trend toward a slight reduction in muscle strength measured by grip dynamometer in both groups • Eating behaviour: no noticeable changes in the enjoyment of food in either group using the Child Eating Behavior Questionnaire. There was a trend toward an increase of satiety responsiveness (*P* = 0.051) and emotional undereating (*P* = 0.08) in the study control
Shroff, 2019 [59]	Non-randomised parallel arm trial	HD/HDF	UK, Europe, and Canada	To compare children on HDF and conventional HD to determine annualised change in cardiovascular endpoints and growth	HD: *n* = 106, range 5–20 yrs, F = 45 (42) HDF: *n* = 71, range 5–20 yrs, F = 43 (60)	Intervention: HD or HDF for 4 h per session 3 times per week PA was assessed through self-report as one of secondary endpoints. It was categorised as in a wheelchair, walks < 30 min per day, walks > 30 min per day, and play sports PA outcomes at the 6 and 12 months: • At 12 months, 44% children on HDF playing sports compared with only 13% of children on HD (*P* < 0.01). The baseline PA levels were not reported
Akber, 2014 [60]	Single-arm trial	Across all stages of CKD, including dialysis and kidney transplantation	USA	To determine whether the intervention would increase steps per day and whether any increase in PA would be associated with improvements in physical function or quality of life in children with CKD	*N* = 44, 15.1 ± 3.4, M = 22 (50), F = 22 (50)	A combination of pedometers and behavioural consultation • Pedometers were used to increase average daily step counts for 12 weeks; and received education about the importance of PA and weekly feedback on targeted step counts, barriers to meeting target activity level, set a new goal for the upcoming week by telephone or email Outcomes • Mean daily step count did not change significantly (+48, 95% CI −48 to +145 steps/day per week). Transplant recipients and patients with CKD increased their activity by 100 steps/day (95% CI −14 to 208) and 73 steps/day (95% CI −115 to 262) each week, respectively, and patients on dialysis decreased by 133 steps/day (95% CI −325 to 58; *P* value for interaction 0.03) • 6MW distance increased by 16 m (95% CI 4, −28) from baseline 508 ± 100 m (*P* value was not provided). CKD stages were associated with changes in 6MWT. Transplant recipients and children on dialysis increased their 6MW distance by 31 m (95% CI 19, 42), *P* < 0.0001 and −28 m (95% CI −60, 5), *P* = 0.008. The difference between transplant and dialysis in increase in 6MWT was significant (−35 m 95% CI −58, −12, *P* = 0.003); 6MW distance improved among transplant recipients who increased their daily steps more than 1000 steps compared with those who did not (37 m 95% CI 26, 71 VS 11 m 95% CI −6.5, 30.5, *P* = 0.006). The change in PA was positively related to the change in 6MWT (*r* = 0.74, *P* < 0.001). The association between steps per day and 6 MWT remained after adjustment for CKD categories (statement provided only) • Self-report physical functioning did not change significantly (−7, 95% CI −15 to +1 points) from baseline 72.3 ± 17.3; CKD category was not significantly associated with change in physical functioning (*P* = 0.18) • The change in physical functioning among transplant recipients who increased their steps by more than 1000 steps/day compared to those who did not was not significantly different (3.1 95% CI 0, 94 VS 0 95% CI −1.6, 10.9, *P* = 0.51) The change in PA was positively related to the change in physical functioning scores (*r* = 0.53, *P* = 0.001). The association between steps per day and physical functioning remained after adjustment for CKD categories (statement provided only)
Johns, 2020 [61]	Single-arm trial	CKD 1–2	USA	To evaluate the feasibility and outcomes of a group-based care in adolescents with early-stage CKD	*N* = 10, median age 18 (IQR 16–19), M = 8 (80%)	Group-based care Group meeting with 2–5 adolescents per month for 6 months to discuss self-activities supported by nephrologist or kidney dietitian. Increasing PA was reinforced at each meeting Outcomes • Adherence: only 40% of participants attending 3 or more sessions • Satisfaction: satisfaction at baseline and 6 months were 90% and 100%, unchanged from baseline and follow-up • Blood pressure: an office BP measured at baseline and 6 months, and mean office SBP was similar at both time points, 127 (SD, 8) versus 125 (SD, 10) mmHg • Self-reported medication nonadherence: decreased from 57 to 40% • Estimated sodium intake from the FFQ also decreased by 800 mg/d at 6 months • Median steps per hour increased from 499 (IQR, 111–712) to 688 (IQR, 302–1107) at 6 months in 5 adolescents who completed the baseline and 6-month measurement • Sodium intake as measured by 24-h urinary excretion and paediatric quality-of-life metrics was largely unchanged at 6 months compared with baseline
van Bergen, 2009 [62]	Single-arm trial	Dialysis	Netherlands and Belgium	To determine the feasibility and efficacy of an exercise training programme to improve exercise capacity and fatigue level in children with kidney failure	*N* = 5, median range 12.6 (8.8–17.6), M = 5 (100)	Traditional mixed cardio and resistance exercise Supervised graded community-based exercise programme, including 5-min warmup, aerobic training, resistance exercises, and active games, and 5 min of cool down; starting after the sixth week of the intervention, patients were instructed to perform exercises at home once a week Outcomes • Cardiopulmonary fitness by 6MWT: 6MW distance tended to be lower after intervention with mean change −0.9 ± 6.8 m (only descriptive, inference test not provided) • Cardiopulmonary fitness by electronically braked cycle ergometer: mean change for peak oxygen uptake (VO_2_max) before and after intervention among 5 children was 12.6 ± 10.3, peak work rate (Wpeak) was 15.6 ± 8.9, VO_2_max/kg and Wpeak tended to be higher after intervention (only descriptive, inference test was not provided) • Muscle strength: muscle strength tended to be higher after intervention with mean change 2.4 ± 20 kg (only descriptive, inference test was not conducted) • Fatigue by subjective fatigue subscale of the self-report questionnaire Checklist Individual Strength-20 (CIS-20): the three patients that filled out the CIS-20 showed a decreased perceived fatigue after the exercise programme (only descriptive, inference test was not conducted) • Quality of life by The Child Health Questionnaire Parent Form 50 (CHQ) reported by parents: HRQOL tended to be unchanged with mean change 0.0 ± 4.5 (only descriptive, inference test was not conducted) Barriers and facilitators • Fifteen patients did not complete the exercise programme and took part in an average of seven sessions • Of these patients, seven did not complete or even start the exercise programme because of a combination of lack of time and motivational problems • Six patients were not able to continue the programme or were unable to perform the follow-up measurements because of medical problems related to their kidney disease • One patient received a kidney transplantation during the intervention period • One patient terminated the exercise programme because of family holidays • None of the patients stopped the exercise programme because of adverse effects of the exercise programme itself, such as musculoskeletal injuries or increased levels of fatigue
Goldstein, 2009 [63]	Single-arm trial	HD	USA	To describe the challenges associated with intradialytic exercise programme implementation in children receiving maintenance HD To assess its impact upon physical functioning in children receiving maintenance HD	*N* = 10, median (range) 13.6 (8–25), M = 6 (60), F = 4 (40)	Traditional mixed cardio and resistance exercise Intradialytic training twice a week, with each session 30–60 min lasting for 3 months. Each session consisted of warming up by arm circles, upper/lower extremity biking, medicine ball toss, arm/leg weights, ball tossing, and Theraband exercises. The sessions were modified depending on the location and placement of HD access, blood pressure cuff, and heart rate monitors Outcomes • Feasibility: 10 (50%) completed the training • Cardiopulmonary fitness: 6MW distance increased from 589 ± 90 to 627 ± 86 yards, *P* < 0.01 • Muscle strength: grip strength increased from 23.2 ± 10.9 to 26.6 ± 12.4 kg, *P* = 0.07; lower extremity testing increased from 70 ± 32 to 94 ± 42 N·m/s, *P* = 0.003 Barriers and facilitators • Patients who were non-adherent gave the following reasons: o Physical limitations such as aches and pains, fatigue, chronic joint pains, and muscle contractures o Already exercised at school during gym classes o A belief that exercises were repetitious or boring, resulting in poor motivation o A lack of family support for providing assistance and motivation for their child’s participation in the exercise • Key factors that were important in those adherent patients o Patients receiving dialysis for less than 1 year were more likely to complete the 3-month exercise o Keep a routine o Encouraging active toys and avoiding sedentary video games and television o Encouraging higher expectations o Identifying hobbies or rewards that motivate o Providing a role model for activity and exercise o Starting with simple activities to engage participation and facilitate success with moving large muscle groups • Finding one patient who is the leader in each dialysis group and help him or her motivate others to participate
Paglialonga, 2014 [64]	Single-arm trial	HD	Italy	To assess the feasibility, acceptability, safety, and efficacy of a short-term intradialytic cycling programme in a population of children, adolescents, and young adults on chronic HD	*N* = 10, median (range) 15.3 (9.1–24.2), M = 5 (50), F = 5 (50)	Traditional cardio intradialytic exercise Intervention: to bike on a cycloergometer with resistance during the first half of the HD sessions (the second hour of treatment) including 3 min warmup bike and 3 min cool down. Two to three times a week depending on the availability of physiotherapist, each session 30 min, 3 months in total of 24 to 36 sessions Outcomes • Feasibility: the median number of exercise sessions/patient was 28.5 (range 23–32) • Enjoyable, that the time on HD passed quickly and that they would like to continue the programme • Cardiopulmonary fitness by 6MWT: the median (range) distance before and after intervention were 552.5 (440–640) m and 585.0 (480–650) m, 6MW distance improved (+4.9%; *P* < 0.01) • Pulmonary function: the median (range) forced vital capacity, FVC(% pred) before and after intervention were 87.2 (77.9–100.1) VS 89.8 (84.6–130.2), decreased 1.2 (−4.8, 5.0), *P* = 0.46; the median (range) forced expiratory volume, FEV(% pred) were 85.4 (68.7–126.7) VS 95.2 (80.5–120.6) increased 1.9 (−4.8, 17.2), *P* = 0.17; the median (range) peak expiratory flow PEF(% pred) were 75.5 (61.2–105.1) VS 78.7 (67.7–91.6), increased 10.8 (−23.4, +19.6), *P* = 0.92 • Muscle strength: the median (range) lower extremity strength before and after interventions were 10 (5–15) kg and 15 (5.3–20) kg, increased by 29.3% (6–37.5), *P* = 0.02; the number of the stands before and after intervention were 39 (18–47) and 41.5 (20–60) increased by 19% (11.1–44.8), *P* = 0.01 Barriers and facilitators o All of the patients (8/8) declared that the exercise programme was enjoyable and the time on HD passed quickly and that they would like to continue the programme
Weigmann-Fasbender, 2020 [37]	Single-arm trial	Transplant	Germany	A home-based exercise intervention with the use of active video gaming is an appropriate tool for improving physical capacity and health-related quality of life	*N* = 13, 12.9 ± 3.4, M = 9 (69.2), F = 4 (30.8)	ehealth exercise cardio and flexibility training programme • Home-based exergaming intervention, Wii fit console of Nintendo, and received regular contact with email and phone call • The training was three times a week, 30 min each session and lasted for 6 weeks with a total of 18 sessions • 5 patients performed the intervention exercises as required and were identified as well compliant • Steps per hour were significantly increased after 6 weeks of exergaming (+31%, *P* = 0.043) • Cardiopulmonary function: mean VO_2_max (1.5 ± 0.6 ml/min VS 1.5 ± 0.5 ml/min, *P* = 0.37, 29.2 ± 9.0 ml/min/kg VS 27.2 ± 8.2 ml/min/kg, *P* = 0.044), peak respiratory exchange ratio (1.11 ± 0.10 VS 1.11 ± 0.11 VCO_2_/VO_2_, *P* = 0.77), peak minute ventilation (56.4 ± 22.2 VS 54.4 ± 19.5 L/min, *P* = 0.57), peak heart rate (166 ± 21.9 VS 163 ± 22.5 bpm, *P* = 0.33), remained unchanged before and after intervention exercise Motor coordination test, maximal handgrip strength, and quality of life remained unchanged (detailed number not provided)
Ramírez-Senent, 2023 [65]	Case series	HD, predialysis	Spain	To report a case series with no options of arterio-venous fistulae in which vessel calibre improvement after isometric exercise allowed for radiocephalic fistula creation in all of them	*N* = 4, median range 16 (13.0–19.0), M = 3 (75), F = 1 (25)	Traditional local muscle resistance exercise Children with predialysis and haemodialysis received isometric exercise programme, 30 contractions with a hand grip device, maintaining 3 s each contraction, and twice a day. Each contraction lasted at least 3 s until the day before surgery 5, 23, 16, and 10 weeks respectively, 13 weeks for median duration, ranged from 5 to 23 weeks Outcomes • AVF Patency: Radiocephalic AVF were performed in the four cases. After a median follow-up of 19 months (range 9–30 months), 75% of patients required further interventions but all of them had a functional AVF • DUS of the forearm cephalic vein and the radial artery: case A < 1.5/2.3 mm, case B 1.5/1.6 mm, case C < 1.5/1.6 mm, and case D 2.1/1.3 mm at the baseline to case A 2.7/2.3 mm, case B 2.5/2 mm, case C 2.8/1.8 mm, and case D 2.7/2 mm
Ahmed, 2001 [66]	Case study	Transplant	USA	To report a case in which a renal allograft sustained a severe anatomic distortion as a result of a bicycle accident	*N* = 1, age = 12, male	Barriers and facilitators Reported a case of a boy who sustained an injury to his renal allograft after a bicycle accident. Despite a dramatic anatomic abnormality seen on magnetic resonance imaging, he suffered only minor and self-limited symptomatic and clinical consequences

*6MWT*, 6-min walk test; *ADPKD*, autosomal dominant polycystic kidney disease; *AVF*, arterio-venous fistulae; *CI*, confidence interval; *CKD*, chronic kidney disease; *CHIP-AE*, Child Health and Illness Profile—Adolescent Edition; *F*, female; *HD*, haemodialysis; *HDF*, haemodiafiltration; *HRQOL*, health-related quality of life; *IQR*, interquartile range; *M*, male; *METs*, metabolic equivalents; *N*, total number; *n*, number of participants in a subgroup; *NK*, not known; *PA*, physical activity; *PAQ*, Physical Activity Questionnaire; *PE*, physical education; *QoL*, quality of life; *RCT*, randomised controlled trial; *VO*_*2*_*max*, maximal oxygen uptake; *VS*, versus

^a^The data analysis was conducted in 401 healthy controls

^b^The study was liver and kidney transplant altogether, without demographic information of the subgroup

^c^Demographic was of all 43 transplant patients including kidney and liver transplantation

^d^PA measurement was taken among the subset of children within 1 year of DXA which was 17 and the demographic was of all 46 participants

^e^PA measurement was taken among the subset of children over 12 years old (290); the demographic information was of 537 full cohort

^f^In total, 411 patients were included, only 388 with physical activity data

### Characteristics of included studies

Most studies were observational (63.8%), conducted in Europe and North America (77.2%), published within 10 years of 2024 (63.2%), and targeted at kidney transplant recipients (56.1%). The median age of participants ranged from 11.0 to 20.0 years, and the mean age ranged from 10.2 to 16 years. The sample size for CKD participants ranged from 1 to 404, with a median of 21 (IQR 13, 40). The included studies comprised 3420 children. Among the 2541 children with reported sex, 1529 (60.8%) were male (Table 3).

**Table 3:** Summary of characteristics of studies in the scoping review

Characteristics	*N* = 57 *n* (%)
Study design	*N* = 58*
Observational studies	37 (63.8)
Cross-sectional studies	30 (51.7)
Retrospective descriptive studies	5 (8.6)
Cohort studies	2 (3.4)
Experimental studies	14 (24.1)
Qualitative studies	5 (8.6)
Case studies	2 (3.4)
Regions	
North America	23 (40.4)
Europe	21 (36.8)
Latin America (Brazil)	4 (7.0)
Middle East	2 (3.5)
Africa (Egypt)	2 (3.5)
Asia Pacific (Korea)	1 (1.8)
Oceania	1 (1.8)
Multicontinental	3 (5.3)
Published year	
2014 onwards	36 (63.2)
Pre-2014	21 (36.8)
CKD stages	
Kidney transplantation	24 (42.1)
CKD 1–5 (excluding KRT)	12 (21.1)
Haemodialysis	9 (15.8)
CKD across stages	6 (10.5)
Dialysis	2 (3.5)
KRT	1 (1.8)
CKD 1–5 and dialysis	2 (3.5)
CKD 1–5 and transplantation	1 (1.8)

*CKD*, chronic kidney disease; *KRT*, kidney replacement therapy;

*One paper reported the results of a cross-sectional study and an interventional study

### Current levels and patterns of PA among children and young people with CKD

Thirty-one observational studies [10–19, 21, 23–27, 29–37, 39–43, 46] (including four retrospective and one prospective cohort study) and four interventional studies [54, 58, 59, 61] described PA levels. More than half of the studies included paediatric kidney transplant recipients (Supplementary Table 3).

#### Measurement of PA

**Fig. 2:**
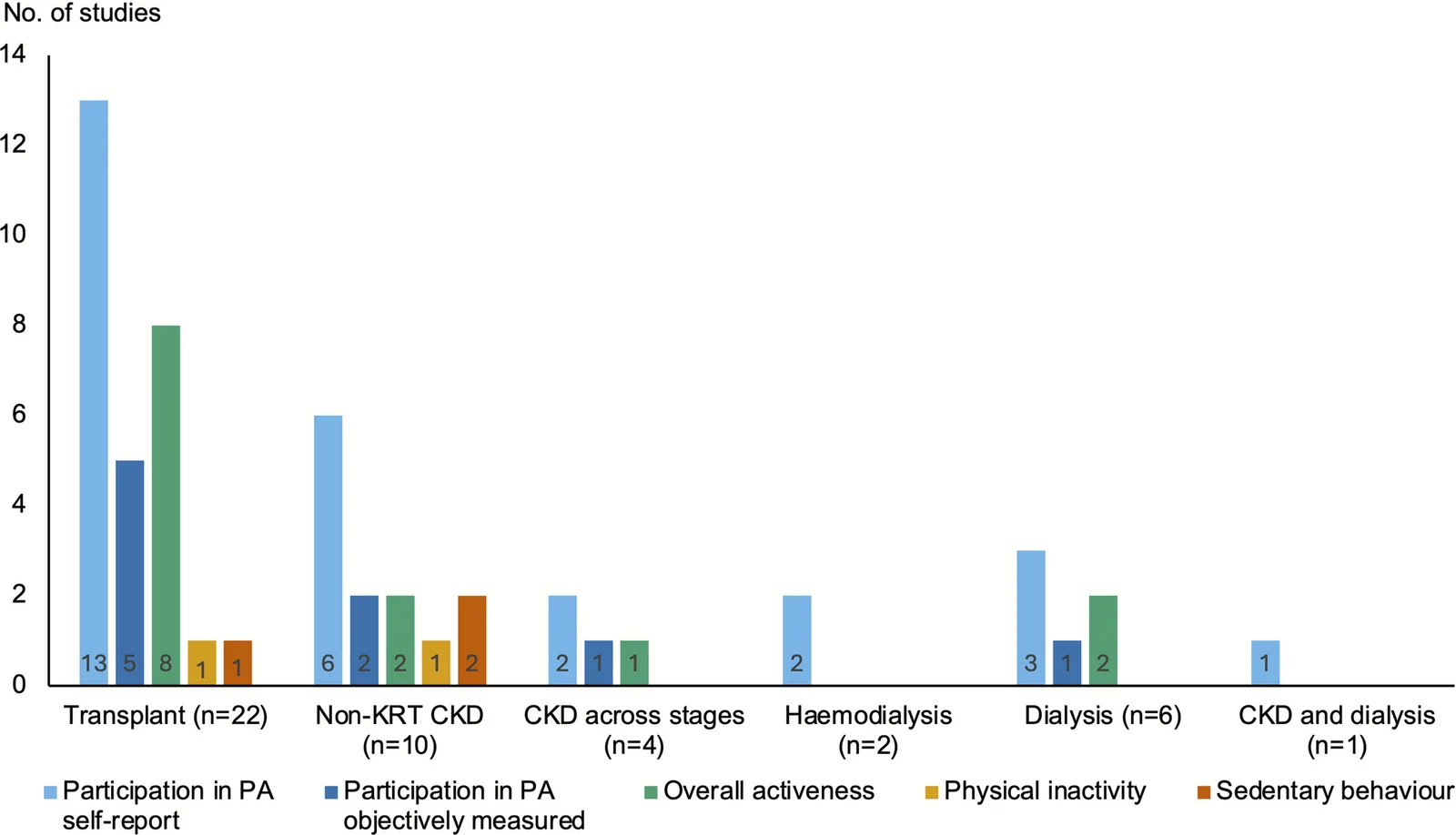
Measurement of PA (*n*=35)

Self-report was the most common method to describe PA participation, overall activeness, physical inactivity, and sedentary behaviours to indicate PA levels in children with CKD (Table 4, Fig. 2). Twenty percent of studies used pedometers [12, 27, 54], accelerometers [24, 29, 61], or multisensors [37] to describe PA, primarily for counting steps [12, 24, 27, 29, 37, 54, 61]. Two studies combined both approaches to assess PA levels [27, 37]. The Physical Activity Questionnaire [19, 27, 35, 36, 39, 41–43] (PAQ-C or/and PAQ-A) was mostly used to assess overall activeness.

**Table 4:** Parameters describing the physical activities among children and young people with chronic kidney disease

Parameters No. studies	Tools	Report
Participation in PA [11, 13–18, 21, 23, 25–27, 29–34, 39, 40, 46, 59] (*n* = 22) • Percentage of individuals participating in PA (general PA, exercise, sports, etc.) • Percentage of individuals reaching recommended levels • Types/settings/volume/intensity/frequency/duration/energy expenditure of PA participation • Number of days spent on PA • The number of sports played or participated in	o Previous Day Physical Activity Recall (PDPAR) [18, 30] o The International Physical Activity Questionnaire (IPAQ) [23] o Youth Risk Behaviour Survey [14, 46] o Habitual Level of Physical Activity (HLPA) questionnaire [11] o The Child Behaviour Checklist [21] (proxy report) o Physical activity recall questionnaire [34] o ‘Motorik–Modul’ PA questionnaire (MoMo-PAQ) [29] o Interviews or self-developed/adapted questionnaires and/or diaries [13–18, 25–27, 30–33, 39, 40, 59]	Self-report
Participation in PA [12, 24, 27, 29, 37, 54, 61] (*n* = 7) • Total steps per day [12, 24, 27, 29, 37, 54, 61] • Time (frequency/duration) spent on various levels of PA [24]	o Pedometers [12, 27, 54] or accelerometery [24, 29, 61] or multisensors (SenseWear) [37]	Device-measured
Overall activeness [10, 19, 27, 35, 36, 39, 41–43, 58] (*n* = 10) • The scores of scales or subscales	o Physical Activity Questionnaire (PAQ-C/A) [19, 27, 35, 36, 39, 41–43] o The Child Health and Illness Profile-Adolescent Edition (CHIP-AE) [10] (Subscales) o Patient-reported outcomes measurement information system (PROMIS) Parent Proxy Items (Physical activity) (proxy report) [58]	Self-report
Physical inactivity [15, 29] (*n* = 2) • Percentage of being physically inactive	o (MoMo-PAQ) [29] (<150 min/week of moderate-intensity physical activity) o Interviews [15] (<150 min/week of moderate-intensity physical activity)	Self-report
Sedentary behaviour [14, 24, 40] (*n* = 3) • Screen time • Sedentary time	o Screen time (hours per day/week) measured by Youth Risk Behaviour Survey [14] and Health Behaviour in School-aged Children Survey [40] o Accelerometers [24]	Self-report Device-measured

*CKD*, chronic kidney disease; *PA*, physical activity

#### Levels of PA

##### Types of PA

**Fig. 3:**
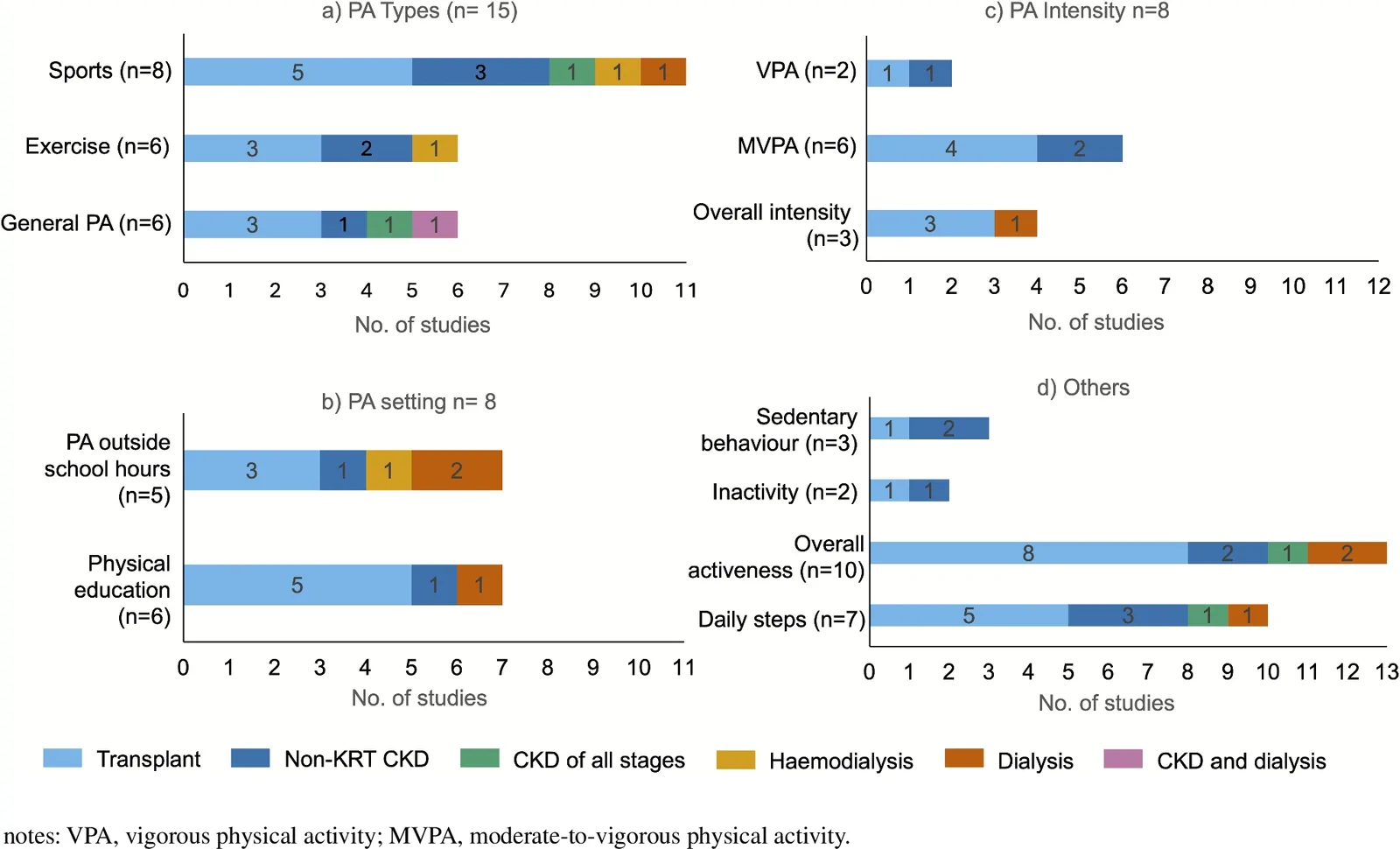
Levels of PA

Fifteen studies [11, 13, 14, 17, 21, 23, 26, 27, 29, 31–33, 39, 46, 59] reported the percentage and duration of participation in sports [13, 14, 26, 27, 29, 39, 46, 59], exercise [14, 21, 31–33, 46], and general PA [11, 14, 17, 23, 27, 39] (Fig. 3a).

Among paediatric transplant recipients, participation in sport ranged from 31 to 64% [13, 26, 39], which was lower than healthy controls [39]. The mean weekly sport time was 129 (95% CI 97, 162) min, lower than that of healthy cohorts across all sports settings and age groups [29]. Exercise participation ranged from 37.5 to 50% [31–33]. Transplant recipients further reported lower PA participation (50% vs. 100%, *P* < 0.0001) [23] and shorter weekly duration (107.3 ± 126.8 vs. 488.2 ± 640.7, *P* = 0.005) compared with healthy controls [27].

Among children on dialysis, participation in sport was 48.8% [13]. Those on haemodiafiltration (HDF) for a year reported higher sports participation than those on HD (44% vs. 13%, *P* < 0.01) [59]. Exercise participation was 23.3% in children on haemodialysis [21].

In children with CKD 1–4, participation in sport was approximately 83% [13]. The mean number of sports played in the past year ranged from 0 [46] to 1 [14]. The median number of days with ≥60 min exercise per week were 3 (IQR 0, 5) [14] and 5 (IQR 3, 7) [46]. Fewer children with non-KRT CKD met WHO PA recommendations compared with healthy controls (13.4% vs. 24.8%, *P* = 0.04) [14].

Across all CKD stages, 48% reported playing no sports [13] and 85% of children did not engage in any regular PA [11]. Compared with CKD 1–2, the incidence rate ratio (IRR) (95% CI) for sports participation among children with CKD 3–5, dialysis, and transplant were 0.84 (0.64–1.09), 0.59 (0.39–0.90), and 0.75 (0.58–0.96), respectively, adjusted for socioeconomic status and ethnicity (*P* = 0.04) [13]. The most common sports across CKD stages were swimming (17%), soccer (17%), football/rugby (12%), dance (9%), and basketball (8%). Participation in football/rugby was higher among children with CKD 1–2 (52%) and CKD 3–5 (28%) than in children treated with dialysis (7%) and with kidney transplants (13%) [13]. No study compared the exercise participation across CKD stages or with healthy controls. One study found that children with CKD 3–5 and those on dialysis had comparable overall PA levels to healthy controls, with similar energy expenditure of PA after adjustment for age, gender, weight, and height [17].

##### Settings of PA

Eight studies [14, 16, 18, 21, 25, 27, 29, 30] described PA levels in school and non-school settings (Fig. 3b). Participation in physical education (PE) ranged from 48 to 75% [18, 25, 27, 30] in transplant recipients and 20% in children on dialysis [18]. Both transplant [18, 30] and dialysis [18] groups spent less than 10% of non-school time on PA. Transplant recipients engaged in vigorous activity mainly at school, which accounted for 26% of moderate to vigorous activity and nearly all vigorous activity [29]. They were reported to be similarly active compared with healthy controls in PE class and had a similar frequency of performing PA outside of school [27]. Children on haemodialysis exercised outside of school, either at home or on the street [21]. Children on dialysis reported comparable daily non-school PA hours compared to healthy controls (median 1.52 IQR 0.78, 2.52 vs. 1.81 IQR 1.17, 2.94) [16].

Children with CKD 1–4 reported a median of 2 PE classes per week with low PA duration per class (median 4 min IQR 3, 5) [14]. Compared to healthy controls (1.81 h), median daily non-school PA was similar in CKD 2–3 (1.91 h, *P* = 0.3) but lower in CKD 4–5 (1.41 h, *P* = 0.03) [16].

##### Intensity of PA

Eight studies [15, 18, 23, 24, 29, 30, 34, 46] reported the overall intensity and PA duration at light, moderate, and vigorous intensities using the metabolic equivalent of task (MET) (defined as the ratio of an activity-specific metabolic rate to the estimated resting metabolic rate [1]) (Fig. 3c). Most studies estimated PA duration at different intensities via recall questionnaires or interviews [15] and adult-based intensity values (METs), with only one study using accelerometers with predefined cutoff values [24].

Transplant recipients engaged in moderate to vigorous PA (MVPA) for 26 (0, 81) min [34] to 69.9 ± 62.1 min [29] per day (via questionnaires), and 29.3 ± 24.6 min per day measured by accelerometers [24]. Vigorous PA (VPA) accounted for 12% of their MVPA duration participation, with a mean of 58 ± 95 min per week [29]. The proportion meeting recommended 60 min/day MVPA ranged from 14 to 48% [29, 34]. Compared with healthy controls, transplant recipients reported a lower proportion achieving ≥3 h weekly MVPA [23]. 

Children on dialysis and transplant recipients reported low average daily MET per activity, ranging from 1.8 METs (light PA) to 4 METs (moderate PA) [18]. Among children with non-KRT CKD, 50% reported more than 150 min MVPA per week [15]. Median VPA days/week was 1 (0, 4), with 46% not performing VPA more than 10 min per week [46]. No comparison of PA intensity was made across CKD stages.

##### Steps, overall activeness, physical inactivity, and sedentary behaviour

Four of seven studies used accelerometers or SenseWear to assess step counts (Fig. 3d). Mean daily steps ranged from 7007 to 9282 ± 4666 in children with transplants [12, 24, 27, 29, 37], 4978 (3058, 6651) or 4788.12 ± 1919.61 in those with non-KRT CKD [12, 54], and 4566 (3500, 9867) in children with dialysis [12]. In early-stage CKD, adolescents had median daily steps of approximately 7000, which increased to 9000 after 6 months of group-based care (assuming 14 waking hours) [61]. Approximately 90% of children across CKD stages were below the recommended daily steps, with a median of 5976 (IQR 3309, 8595), which was 44% lower than healthy cohorts [12]. Differences in steps between CKD 1–4, transplants, and dialysis were consistent with chance (*P* = 0.10) [12].

In children with transplants, median PAQ scores ranged from 2.1 (IQR 1.6, 2.8) to 2.4 (IQR 1.4, 3.0) [39, 42, 43] out of 5, and mean scores ranged from 2.16 ± 0.75 to 2.8 ± 0.8 [27, 35, 36, 41], which were lower than in healthy children in one study [39] but comparable in another study [27]. Among children on dialysis, mean PAQ scores were 2.5 ± 0.7 [19]. In children with CKD 2–4 with anaemia, scores from PROMIS (Parent Proxy Physical Activity Items) ranged from 43.8 ± 9.6 to 45.0 ± 6.9 [58]. Children across CKD stages reported a lower score from the subscale of the CHIP-AE compared to healthy controls [10]. The scores were lower in the KRT group compared to the non-KRT group, with dialysis patients scoring lowest, followed by transplant recipients [10].

Transplant recipients reported 29% of physical inactivity (<120 min/week of MVPA) [29]. Accelerometer data showed they spent 59% of their daily time sedentary, with a mean of 476.1 ± 174.3 min per day [24]. Among children with non-KRT CKD, 50% were physically inactive (<150 min/week of MVPA) [15]. They reported a mean screen time of 10.1 ± 5.1 h per week [40]. Compared with healthy controls, fewer children met the recommended threshold of ≤2 h/day of entertainment screen time (1.8% vs. 27%, *P* < 0.001) [14].

#### Comparisons of PA levels in children with CKD

##### PA levels compared to healthy controls

Twelve studies included healthy controls [10, 12, 14–17, 19, 23, 27, 29, 37, 39] to compare sport participation, general PA participation, non-school PA levels, step counts, and PAQ scores. Compared with healthy controls, transplant recipients reported less weekly sports time [29], engaged in different sport types [27], had reduced regular PA participation [23], shorter weekly PA duration [27], lower step counts [37], and lower PAQ scores [39]. However, some studies reported comparable level of activity in PE, non-school PA duration, step counts, and PAQ scores [27]. Children on dialysis reported similar non-school PA duration [16] and PAQ scores [19]. Among those with non-KRT CKD, fewer met PA [14, 15] and screen time recommendations [14]. Daily non-school PA duration was comparable in CKD 1–2 but lower in CKD 4–5 [16]. Across all stages, children reported 44% fewer steps [12] and lower CHIP-AE scores [10] but comparable PA-related energy expenditure when adjusted for covariates [17].

##### PA levels across CKD stages and treatment modalities

Six studies compared PA levels across CKD stages (1–2 vs. 3–5), KRT vs. non-KRT CKD, and transplant vs. dialysis, and HD vs. HDF [10, 12, 13, 15, 17, 18, 59]. Across CKD stages, self-reported activeness scores were comparable between CKD 1–2 and CKD 3–5 [10]. Self-reported PA (activeness and sport participation) were lower in those on KRT, particularly among children on dialysis [10, 13]. One study found that physical inactivity tended to increase from CKD 3 to 5, although no detailed figures were provided [15]. However, device-based measurements found that daily step counts were comparable across transplant, CKD 1–4, and dialysis groups [12]. The intensity and duration of non-school physical activity were also similar between dialysis and transplant groups [18]. One non-RCT found that children on HDF for a year reported higher sport participation compared with those on HD [59].

##### PA levels by demographic factors and over time

Two studies compared PA levels by sex with inconsistent findings [12, 14]. One reported higher step counts in male children with CKD across all stages [12], whilst the other found boys and girls with non-KRT CKD equally likely to meet PA recommendations [14]. Three studies reported lower PA levels in older children [12, 27, 39], with fewer daily steps in those aged 18–20 years across CKD stages than those younger peers [12], and PAQ scores in transplant recipients declining with age [27, 39].

Four studies reported a declining trend in PA levels over time [14, 18, 39, 40] with retrospective [39, 40] and subgroup longitudinal data [14, 18]. Following transplantation, 67% of children with CKD reported increased PA [39], although another study found a decline [18]. In children with CKD 1–4, PA hours fell (7.9 to 3.7 h/week) and screen time increased (10.1 to 26 h/week) 6 months after lockdown [40]. Within 1 year, the proportion meeting PA recommendations (14.7 to 7.5%) and sports participation (57 to 48%) decreased [14].

#### Relationships between levels of PA and outcomes

**Fig. 4:**
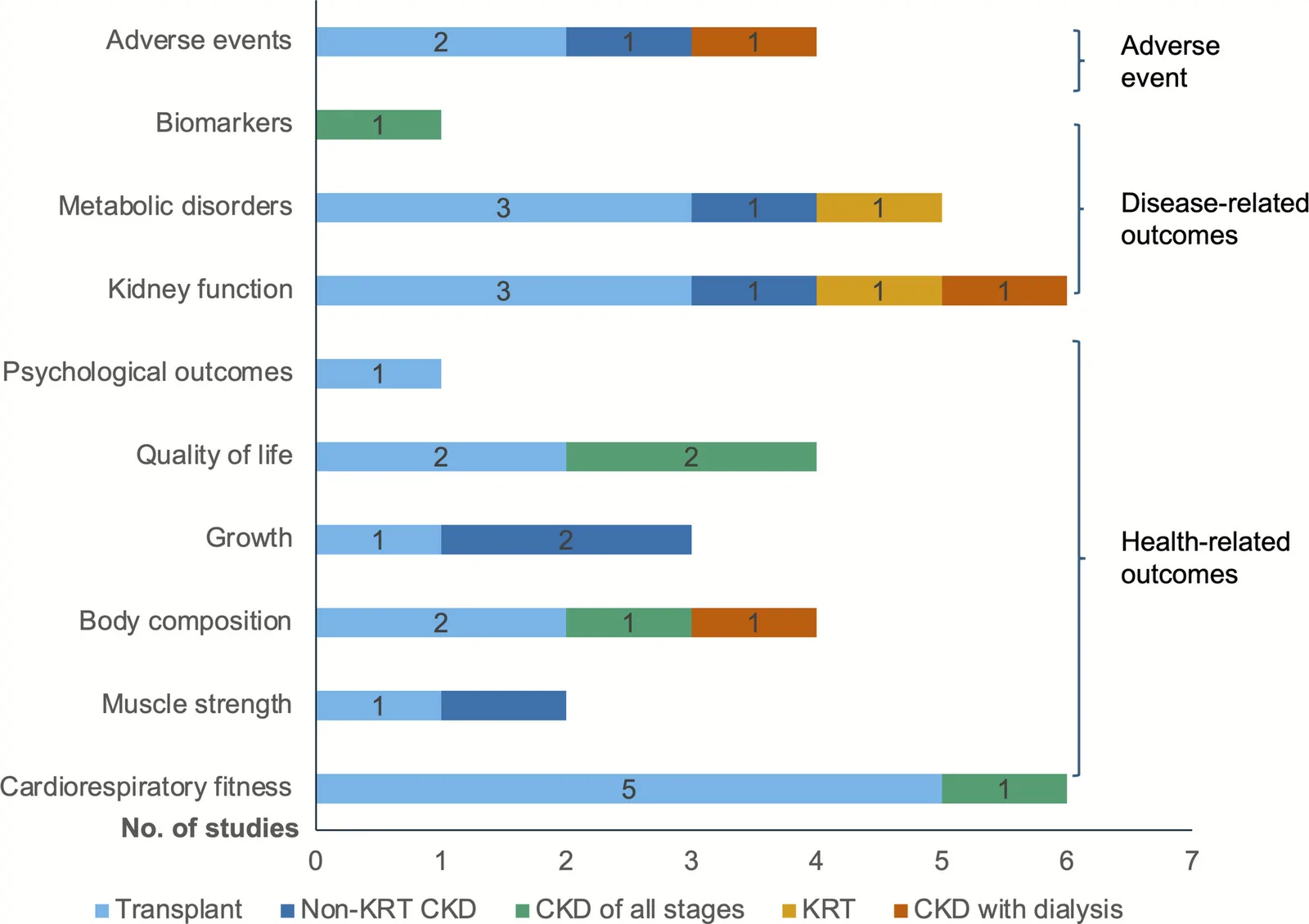
Outcomes reported in studies describing physical activity (n=19)

Nineteen studies investigated the relationships between levels of PA with health-related [11, 12, 16, 27, 29, 31, 32, 34, 37, 41, 45, 46], disease-related [12, 14, 17, 18, 29, 32, 34, 40] and adverse outcomes [20, 39, 45, 66]. Cardiorespiratory fitness/function and kidney function were mostly reported, followed by metabolic disorders, quality of life, body composition, and adverse events (Fig. 4, Supplementary Table 4). Positive correlations or tendencies were found between VO_2_max or 6MWD and PA levels in transplant recipients [31, 32, 34] and in children with CKD across stages [12]. Regarding the association between kidney function and PA levels, five cross-sectional studies reported mixed findings among transplant recipients and non-transplant CKD patients [14, 17, 27, 29, 32]. One retrospective study reported that PA and screen time were not associated with worsening CKD before and after lockdown [40]. No prospective longitudinal studies have examined the associations between physical activity and CKD progression.

### The experience of children and young people with chronic kidney disease engaging in PA

No studies directly described the experience of engaging in PA. However, children and young people described the impacts of CKD or its treatment on PA or exercising as a coping method in one cross-sectional study [22] and five qualitative studies [47–51]. The cross-sectional study described children’s perceived impacts of CKD and haemodialysis on their participation in different types of sports [22]. The five qualitative studies in adolescents explored the impacts of kidney transplantation [51], autosomal dominant polycystic kidney disease (ADPKD) [47], and short stature from CKD across stages [50] as well as a coping strategy for CKD 4–5 [49] and self-management of chronic nephritis [48] (Table 5).

**Table 5:** Findings of qualitative studies in children and young people with chronic kidney disease

Theme 1	Having difficulties in physical activity in adolescents with CKD
Summary	Adolescents reported having difficulties in physical activity in early stages of CKD [47, 48], after transplantation [51], and across CKD stages [50].
‘Difficulty in physical activity’ and ‘restriction in daily life’ in children with chronic nephritis [48]. • *‘A little bit, if I walk for a long time, participate in sports events, or exercise for a long time, my body doesn't follow.’* ‘Impact on sports or physical activity’ in children with autosomal dominant polycystic kidney disease, ADPKD [47]. • *‘I need to use my body sparingly. For example, I try to avoid running, because I am afraid it will affect my health.’*. • *Ten adolescences (30%) avoided sports based on the advice of their doctor or parent because they experienced pain or discomfort during physical activity, or for other reasons. More females reportedly avoiding sports 6 (42.9%), compared to males 4 (21.1%).* ‘Physical changes after kidney transplantation in adolescence’, difficulty in exercising is one of the most significant physical changes [51]. • *‘Sometimes I play. But I don't play much because I could get hit in the kidney.’* ‘Constrained life participation and enjoyment’ due to the short stature in children across CKD stages [50]. • *‘They say, in volleyball and basketball, that I can’t play because I am too short.’* • *‘He can’t mess around with the cousins that are of the same age and my youngest son who’s the same age. It’s hard because he is so small he’s going to get hurt easier, and it’s upsetting to me because he wants to be there. ’*	
Theme 2	PA was adopted as a coping strategy in children with CKD
Summary	Adolescents mentioned about using physical activity as a coping strategy to manage CKD 4–5 [49] and chronic nephritis [48].
‘Controlling physical activity’ as one of a strategy for self-management in children with chronic nephritis [48]. • *‘In middle school, I didn't participate in soccer or baseball, and I only played badminton or table tennis because there was a performance evaluation in high school.’.* • *I relieve stress through exercise, but since I can't exercise, I think if I do some moderate exercise... When I exercise, I deliberately lift heavy things. If I lift and put it down like that and sweat while feeling a sense of achievement, it gives me a sense of achievement.’* ‘Exercise and pastimes’ as coping strategies in adolescences with CKD 4–5 [49]. • *‘I’m not great at sport but I enjoy doing it’* • *‘I think any activities can sort-of takes my mind off it (health related concerns); football, running’*	

Italic text represents quotes taken directly from the original papers

## Interventions developed to promote or encourage PA among children and young people with chronic kidney disease

One case series [65] and fourteen experimental studies, including six RCTs [53–58], six single-arm before-and-after studies [37, 60–64], one non-randomised parallel-arm trial [59] and one with historic control [52], reported PA-related intervention or interventions to improve PA. Ten of 15 studies were in children on KRT, especially on haemodialysis/dialysis (*n* = 7). The sample sizes of interventional studies ranged from 4 to 177, with a median of 21 (IQR 10, 44) (Supplementary Table 5).

### Characteristics of interventions

**Fig. 5:**
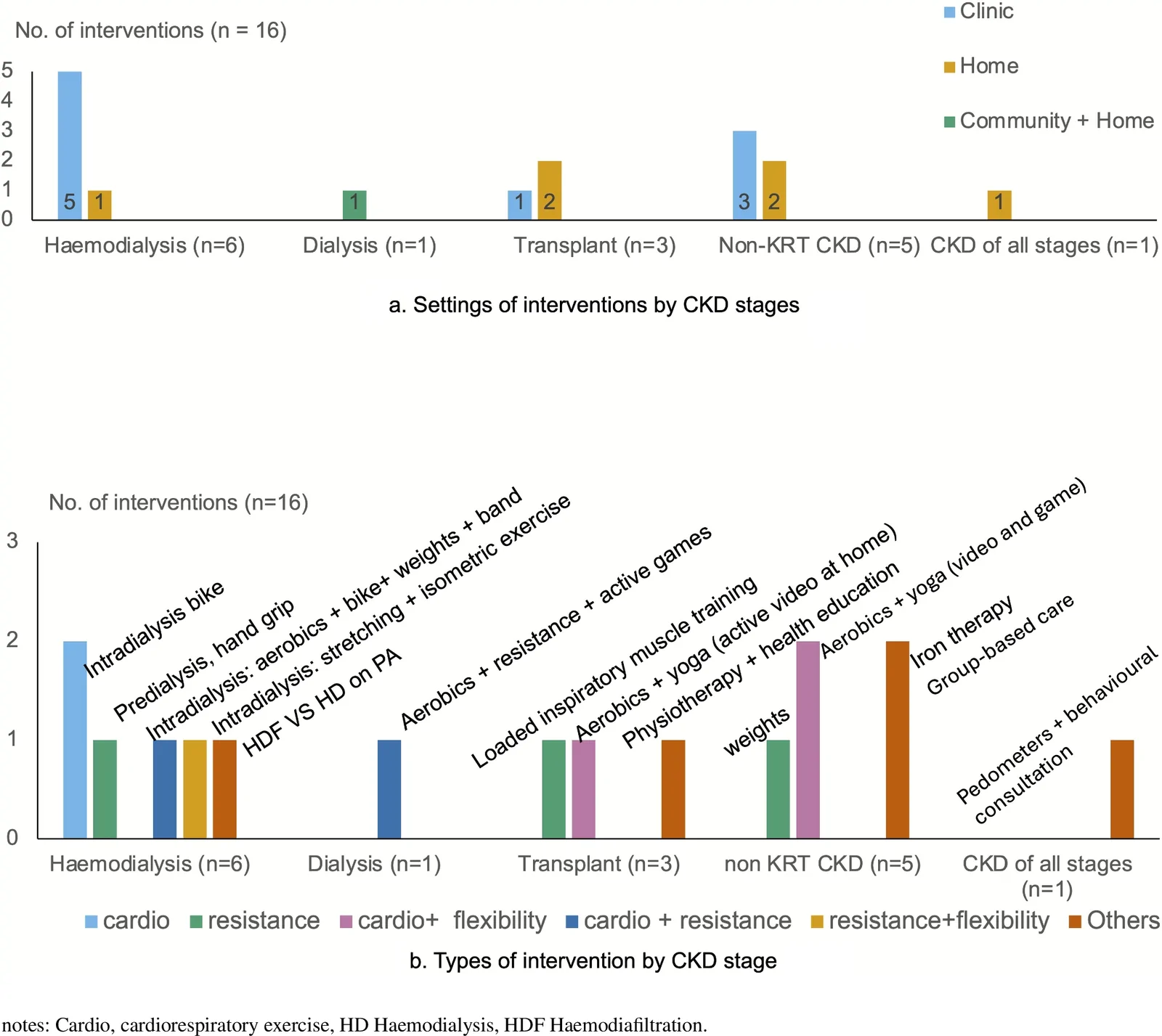
Characteristics of interventions

Ten studies evaluated 11 exercise training interventions, with one study comparing two interventions: exergaming (interactive exercise video games) at the clinic and a home active video intervention [54]. One study adopted PA promotion strategies through early post-transplantation physiotherapy and health education in a clinic setting [52]. The remaining four studies included PA as one of the outcomes of interventions examining a combination of pedometers and behavioural consultation [60], group-based care [61], different modalities of haemodialysis [59] and iron therapy in children with CKD 2–4 and anaemia [58]. These interventions were most frequently delivered individually, either at home (unsupervised) or in clinical settings (supervised) (Fig. 5a). Endurance training interventions were mostly used in the training programmes (Fig. 5b). In children with dialysis, six studies examined intradialytic exercise (ergometer bikes [55, 63, 64] and/or resistance training [57, 63]), mixed aerobic and resistance training during the interdialysis period [62], and predialysis isometric exercise training [65] (Fig. 5b).

#### Type, frequency, duration, intensity, and volume of exercise interventions included

Eight [53, 55–57, 62–65] out of ten studies were traditional exercise training programmes, and two studies [37, 54] adopted e-health interventions including Nintendo Wii-based exergaming [37, 54] and home active video [54]. Each exercise session lasted from 20 to 90 min. The frequency of the intervention ranged from twice a day to seven times a week. Programmes lasted from 6 weeks to 6 months. The weekly training volume ranged from 21 to 210 min, with the total training minutes ranging from 210 to 2520. Six studies specified the training intensity [37, 53, 55, 56, 62, 64] measured by heart rate [62, 64], perceived exertion rate [37, 64], or percentage of repetition maximum [53] (Table 6). Eight studies personalised aspects of resistance in weight training [53, 56, 65], intensity in endurance training [54, 55, 62, 64], and intensity and types of exercise [63]. Seventy percent of the exercise programmes included progression in terms of increasing resistance [53, 63], exercise repetitions [53, 54, 57], intensity [64], duration [52, 54, 57], change of settings, and adding new exercise types [62].

**Table 6:** Characteristics of studies testing exercise interventions (*N* = 10)

Characteristics	No. of studies *n* (%)
Time per session (min)	
≤30 min	4 (40)
>30 min ≤ 70 min	5 (50)
>70 min	1 (10)
Frequency (times per week)	
≤3	8 (70)
>3 ≤ 7	1 (10)
>7	1 (10)
Durations (weeks)	
≤6	3 (30)
>6 ≤ 12	5 (50)
>12	2 (20)
Intensity	
Light	3 (30)
Moderate	1 (10)
Moderate to hard	2 (20)
Unknown	4 (40)
Volume per week (min)	
≤60	3 (30)
>60 ≤ 120	5 (50)
>120	2 (20)
Volume in total (min) (Cumulative amount of time spent on intervention during study period)	
≤720	4 (40)
>720 ≤ 1440	5 (50)
>1440	1 (10)

### Main outcomes of interventions

One study that included PA promotion strategies reported improvements in VO_2_max, quality of life, and Strength and Difficulties Questionnaires [52]. Among four studies included PA as primary or secondary outcomes, iron therapy did not increase proxy-reported PA levels in a pilot RCT involving children with CKD 2–4 and anaemia [58]. In a non-randomised controlled trial, children receiving HDF for a year reported higher sports participation than those on HD [59]. Two single-arm trials indicated either similar PA levels or a trend of improvements after intervention [60, 61]. Among exercise interventions, four studies assessed intervention feasibility, four in dialysis or predialysis participants, with intervention completion rates ranging from 20 to 100% [62–65]. Cardiopulmonary fitness was the most frequently reported outcome across CKD stages, followed by quality of life and muscle strength. Other outcomes included daily steps and fatigue. Disease-related outcomes and adverse events were measured only in one study where Kt/V was reported in children on HD as the primary outcomes, and adverse events such as bleeding and dislocation of the dialysis needles were noted (Table 7, Supplementary Table 6).

**Table 7:** Outcomes of studies evaluating exercise interventions in children and young people with chronic kidney disease

	Transplant *n* = 2	Dialysis *n* = 1	Non-KRT CKD *n* = 2	HD *n* = 5
Feasibility	△[37]	△[62]		△[63] △[64] ☐[65]
6WMD	☆[56] ↦	△[62] ↦	☆[53] ↥ ☆[54] ↦*	△[63] ↥ △[64] ↥
VO_2_max	△[37] ↦	△[62] ↦		
QoL	△[37] ↦	△[62] ↦	☆[53]↥ ☆[54] ↥	☆[57] ↥ ☆[55] ↦
Daily steps	△[37] ↥	△[62] ↦	☆[54] ↥	
Muscle strength	△[37] ↦ (handgrip) ☆[56] ↥ (maximal Insp/Exp pressure)	△[62] ↦	☆[54] ↥ (left knee flexion)	△[63] ↦ (handgrip) △[63] ↥ (lower extremity) △[64] ↥ (lower extremity) △[64] ↥ (the number of stands)
Fatigue	☆[56] ↦	△[62] ↦	☆[54] ↧	
Others	☆[56] ↥ Haemoglobin ↦ Pulmonary function, motor coordination test ↦ Creatinine, urea, potassium, and phosphorus			☆[55] ↦ Single Kt/V ↦ Change of solute removal, etc. ↦ Adverse events △[64] ↦ Pulmonary function ☐[65] ↥ AVF patency and vessel calibre

☆ = randomised controlled trial; △ = single-arm study; ☐ = case series; ↦ = the change is consistent with chance or inference test not provided; ↥ = increase; ↧ = decrease

*6MWD*, 6-min walk distance; *AVF*, arterio-venous fistulae; *CKD*, chronic kidney disease; *Insp/Exp*, inspirator/expiratory; *HD*, haemodialysis; *KRT*, kidney replacement therapy; *QoL*, quality of life; *VO*_*2*_*max*, maximal oxygen consumption

^*^Cavusoglu [54] compared supervised Nintendo Wii-based exergaming with home-based fun video exercise

### Barriers or facilitators of engaging in PA among children and young adults with chronic kidney disease

**Fig. 6:**
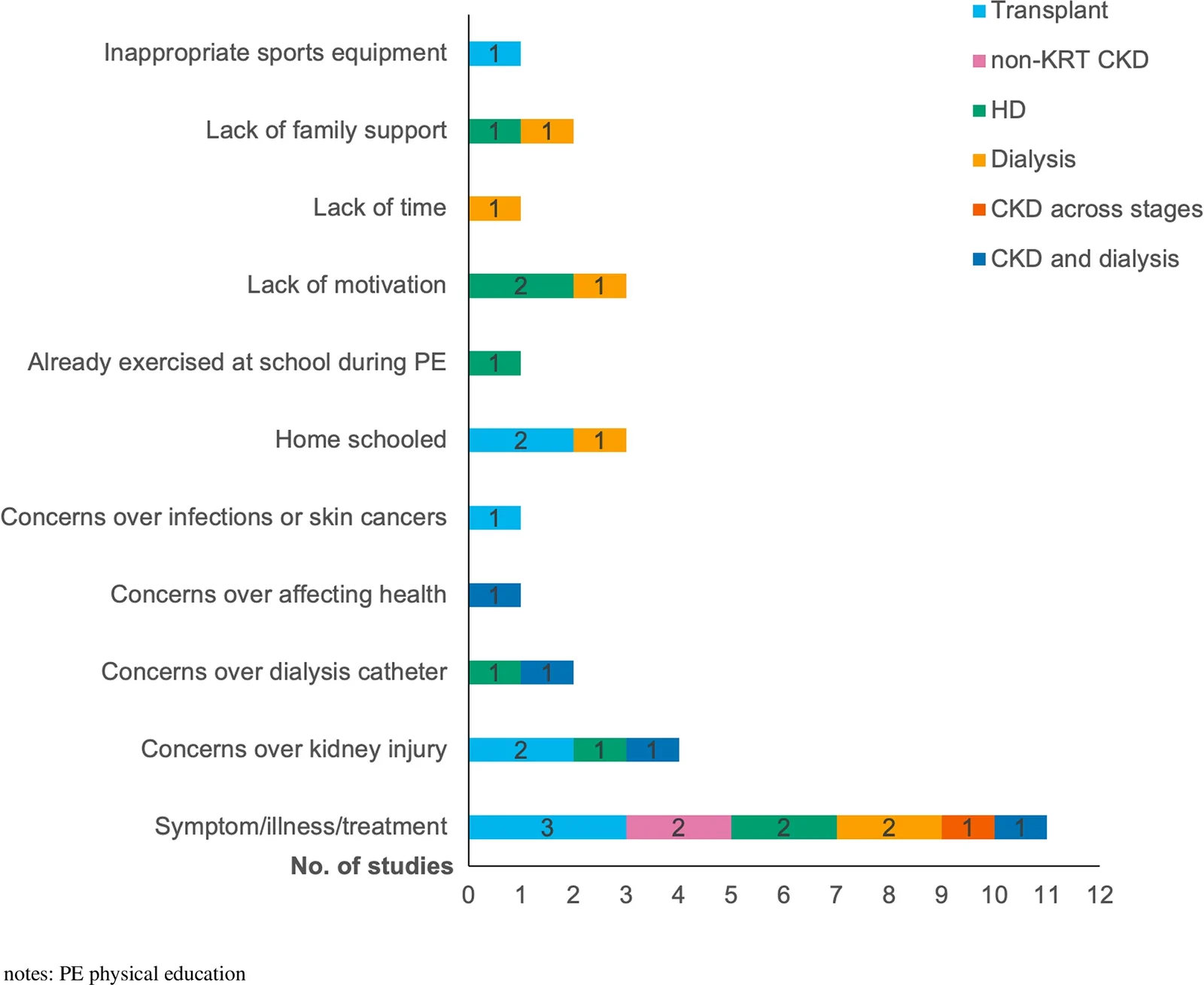
Barriers to engaging in PA (*n*=16)

Four qualitative studies [47, 48, 50, 51] reported CKD limited children’s PA, and seven observational studies [18, 20, 21, 28, 30, 38, 39] identified the reasons for non-participation. Five interventional studies [55, 56, 62–64] reported reasons for attrition [55], low adherence [56, 62, 63] and potential facilitators of participation [63, 64]. Across CKD stages, the most common barriers to PA were compromised physical conditions due to illness, treatment, symptoms, and short stature [18, 21, 39, 47, 48, 50, 51, 62, 63] as well as concerns about adverse events [21, 39, 47, 51, 55]. Transplant recipients reported additional barriers including concerns over infection and skin cancer, home schooling, and inappropriate sports equipment [38]. Children on dialysis described the greatest number of barriers, including concerns over dialysis access, physical education arrangements (home schooled, etc.) [18, 30, 48, 63], lack of time or motivation [55, 62, 63] and limited family support [62, 63] (Fig. 6). Facilitators for intervention adherence included enjoyment [64], role modelling, and peer support [63].

Clinicians’ recommendations play an important role in both encouraging and limiting PA in children across CKD stages [44, 47] and treatment modalities [39]. Surveys of physicians’ recommendations on sports participation in haemodialysis [20] and transplant [28] found mixed responses regarding weight training [20] and some ball games [28]. Children with transplant were more often advised to limit rather than increase PA [39]. In electronic medical records of 404 children with CKD and transplants, 29.6% had a charted PA recommendation, which were more likely in older patients (OR 1.08, 95% CI 1.03–1.13), those with higher BMI z scores (OR 1.45, 95% CI 0.96, 1.51), higher height z scores (OR 1.55, 95% CI 1.31, 1.82), higher eGFR (OR 1.01, 95% CI 1.01, 1.02), and those already participating in sports (OR 2.47, 95% CI 1.46, 4.19), but less likely for children with higher motor scores (OR 0.6, 95% CI 0.41, 0.87) [44].

## Discussion

This scoping review aimed to present the available evidence relating to PA with CKD and found high heterogeneity in study findings. The current evidence mainly described PA levels among paediatric kidney transplant recipients in cross-sectional studies in Europe and North America, generally with relatively small sample sizes except for two regional cohort studies (CKD 1–4 in CKiD in the USA and Canada [14, 46] and CKD across stages in KCAD conducted in Austria and New Zealand [13]). Children of younger age and those on peritoneal dialysis were underrepresented in the included studies, limiting the generalisability of findings. The studies have identified potential important factors influencing physical activity levels—including age, sex, and CKD stages—as well as potential associations with outcomes such as cardiopulmonary fitness, muscle strength, quality of life, growth, and dialysis efficacy. No prospective longitudinal studies were identified that examined associations between physical activity and CKD progression. Nor did any explicitly describe children’s/carer’s experience of engaging in physical activity. Barriers such as safety and health concerns, lack of time and motivation, and facilitators such as fun and enjoyment were extracted from survey findings or results of interventional studies. Most interventional studies were traditional, home-training programmes targeting endurance, resistance, or flexibility with single-arm design in children on haemodialysis.

We found great heterogeneity in the measurement and report of PA in children with CKD. Inconsistency exists between subjective and objective measurements, among various tools, and between self-reports and parents’ proxy reports, making it challenging to compare PA levels across studies in some cases. The challenge of capturing PA levels in children with CKD is partly due to the multidimensional and context-specific nature of PA [67]. Most studies relied on the self-report method to measure PA in different domains and contexts; however, it is subject to recall bias or proxy report bias, especially for younger children. Accelerometers, which are considered more accurate for the capture of PA intensity, were used in four studies mainly to describe steps in adolescent transplant recipients and may be biased by low adherence [29]. The use of device-based measurements to quantify PA at different intensity needs to be further explored in children with CKD, with limited evidence currently available in the existing literature. Like children with other chronic diseases, children with CKD lack questionnaires to adequately quantify PA, as highlighted by a systematic review [67]. PAQ-A/C, the most used questionnaire to describe the general level of PA in children with CKD, is only appropriate during the school year [68]. However, the fact that children with CKD experience relatively high absenteeism [69, 70] may compromise its accuracy. Moreover, heterogeneity in CKD severity, treatment modality, and the rarity and complexity of underlying causes further complicate PA assessment [70]. This implies that adopting a combination of tools and reporting methods, tailored to both treatment context and underlying disease, might better capture patterns of PA among children with CKD.

Compared to healthy controls, among whom physical inactivity and sedentary lifestyle are prevalent, with 81% of adolescents aged 11 to 17 years failing to meet WHO recommendations on PA for health [1], children with CKD had similar or worse levels of physical inactivity. According to WHO 2020 guidelines on PA and sedentary behaviour, those with chronic medical conditions and/or disability should try to meet these recommendations where possible and as able [1]. In the latest KDIGO guideline, children with CKD are encouraged to undertake PA aiming for WHO-advised levels (i.e. ≥60 min daily) [71]. This implies children with CKD might be lagging in the chance of acquiring health benefits of being active, and thus understanding and promoting PA among children with CKD is urgently needed. As many studies were cross-sectional, it remains unclear whether PA preferences change with disease progression or age. This highlights the need for prospective, longitudinal studies, as well as studies that examine PA by granular subgroups such as dialysis modality and disease type.

PA interventions in children with CKD are limited in form, content, and are less tailored to CKD stage compared to those designed for adults. The interventional studies promoting PA among children with CKD were mostly on an individual level, in children on haemodialysis, which adopted traditional exercise training methods, with a focus particularly on endurance training, and lasting at most 6 months. In adults with CKD, more diverse exercise programmes and interventions to promote PA have been tested [72, 73]. An umbrella review of systematic reviews summarised that in adults with CKD, exercise had a moderate positive effect on cardiorespiratory fitness, muscle strength, and body composition and a small positive effect on muscle endurance, cardiovascular risk factors, dialysis-related symptoms, and health-related quality of life outcomes [73]. In the paediatric CKD population, two systematic reviews [74, 75], based on five clinical trials, suggested that the 6-min walking distance (6MWD) did not improve following exercise programmes, with only low grade evidence. The discrepancies in heterogeneity estimates and effect sizes among these reviews highlight the challenge of summarising the impact of exercise in children with CKD due to small sample sizes, high dropout rates, single-arm designs, and heterogeneity across studies. One systematic review [75] further explored the heterogeneity using subgroup and meta regression analysis, and found that studies with longer intervention duration, longer session duration, and better methodological quality tended to report greater improvements in 6MWD and quality of life. However, given the limited number of studies and methodological issues, more rigorous interventional trials are needed in this population to explore the benefits of exercise.

## Strengths and limitations

This scoping review is comprehensive and inclusive of children’s age ranges, languages, study types, PA definition, and CKD stages. It offers a broad overview of the evidence of PA in children and young people with CKD based on a comprehensive literature search. To our knowledge, this is the first scoping review to map both quantitative and qualitative studies on PA in this population, covering measurements, levels, interventions, and outcomes, as well as experience and barriers and facilitators.

Due to the narrative nature of scoping reviews, quality assessment of the included studies was not undertaken and syntheses of effect size of PA interventions on the outcomes or associations between PA and outcomes were not performed. The heterogeneity in measurement and reporting levels of PA make it challenging to compare across the studies. What is more, topics such as physical functioning or exercise capacity were beyond the focus of this review.

## Implications for future research

In conclusion, evidence on physical activity in children with CKD is predominantly quantitative, relying on self-reported data, and focusing largely on kidney transplant recipients. Studies in children with CKD are still needed to address these key gaps: providing more precise PA estimates, reducing the heterogeneity in PA and its outcomes, understanding longitudinal PA change, capturing stakeholders’ perspectives on PA participation and developing feasible and effective PA interventions (Table 8).

**Table 8:** Implications for future research

Future research is needed, including:
To provide better PA estimates in diverse populations
• Studies that examine children’s PA patterns across CKD stages and treatment modalities, including age-appropriate types, settings, and intensities, particularly in younger children under 12 years • Studies based on regional cohorts or registry data, allowing for larger sample sizes, more precise effect estimates, and greater generalisability across diverse populations •Studies reporting PA data from different regions, countries, ethnicities, and cultures, especially from currently underrepresented areas • Studies presenting discrete PA data by CKD stages (especially non-transplant patients), primary kidney diseases, and key demographic features such as age and sex
To reduce the heterogeneity and measure PA comprehensively
• Studies exploring adherence, feasibility, and efficacy of device-based measures to capture PA at different intensities in children with CKD, especially in younger children • Studies assessing the reliability and validity of self-report or proxy-report tools, including questionnaires or scales • Studies incorporating device-based measures and/or validated self-report methods in measuring and reporting PA, especially in non-transplant populations such as peritoneal dialysis • Studies identifying key parameters of PA measurement and/or relevant patient-reported outcomes that are important to children and young people and their families
To understand longitudinal PA change
• Studies investigating longitudinal changes in PA patterns across CKD and treatment progression, especially with prospective study designs
To capture stakeholders’ perspectives on PA participation
• Studies exploring experiences, preferences, and attitudes of PA participation from children’s and carers’ perspectives across the CKD stages, in both children and adolescents • Studies exploring attitudes, practices, and recommendations of PA among healthcare providers to children with CKD across CKD stages, not limited to haemodialysis and transplant
To develop and test interventions across CKD and developmental stages
• Studies engaging children and relevant stakeholders (carers, healthcare providers) in designing more acceptable, diverse, and enjoyable interventions. This requires tailoring interventions to children’s developmental stages (beyond adolescence) and to different CKD stages (beyond intradialytic exercise) as well as incorporating broader PA types (not only the structured exercise programmes) • Studies evaluating PA interventions beyond health-related outcomes such as cardiopulmonary fitness and quality of life, to also assess kidney disease-related outcomes and adverse events

## Supplementary Information

Below is the link to the electronic supplementary material.

ESM1 (DOCX 250 KB)

ESM2 (DOCX 410 KB)

Graphical Abstract (PPTX 125 KB)

## Data Availability

No new data were generated or analysed in support of this research.
