# Supplementary material for: Physical activity among children and young people with chronic kidney diseases: a scoping review
Source: Pediatr Nephrol. 2026 Apr 6;41(11):3713–47. doi: 10.1007/s00467-025-07142-0 (PMC13612716; doi:10.1007/s00467-025-07142-0)
Supplement: Supplementary file 1 [file 467_2025_7142_MOESM1_ESM.docx]

Supplement 1 Search Strategies

**Database: Ovid MEDLINE(R) ALL <1946 to February 19, 2024>**
**Search Strategy:**
**1**  exp Renal Insufficiency, Chronic/ or exp Uremia/ or exp renal replacement therapy/ or "Chronic Kidney Disease-Mineral and Bone Disorder"/ (321298)
**2**  ((chronic* or progressi*) adj1 (renal* or kidney*)).tw. (114123)
**3**  ((kidney* or renal*) adj1 insufficien*).tw. (24618)
**4**  (ckd or ((kidney* or renal*) adj1 disease*)).tw. (179230)
**5**  ((kidney* or renal*) adj1 fail*).tw. (105466)
**6**  ((endstage* or end-stage* or "end stage*") adj1 (renal* or kidney*)).tw. (51865)
**7**  (esrd or eskd or esrf or eskf).tw. (22833)
**8**  ((renal or kidney) adj2 replace*).tw. (20208)
**9**  (hemodialys* or haemodialys* or peritoneal dialysis or RRT or KRT).tw. (115145)
**10**  ((kidney* or renal) adj3 (transplant* or graft* or allograft*)).tw. (109690)
**11**  (CAPD or APD or NIPD).tw. (13115)
**12**  dialys*.tw. (131715)
**13**  (artificial adj1 kidney*).tw. (1594)
**14**  1 or 2 or 3 or 4 or 5 or 6 or 7 or 8 or 9 or 10 or 11 or 12 or 13 (568168)
**15**  exp Exercise Test/ or exp Exercise/ (304686)
**16**  exp Sports/ (220979)
**17**  exp Physical Fitness/ or exp Physical Exertion/ (91746)
**18**  exp Mind-Body Therapies/ (47981)
**19**  exp Exercise Therapy/ (65091)
**20**  exp Exercise Movement Techniques/ (10592)
**21**  (exercis* or physical therap* or physical activity or physical inactiv* or sport or sports or movement or mobility or sedentar* or physiotherapy* or muscle training or strength training or functional therap* or kinesiotherap* or resistance training or resistance program* or physical fitness).tw. (1053655)
**22**  15 or 16 or 17 or 18 or 19 or 20 or 21 (1271092)
**23**  exp Puberty/ (19285)
**24**  exp Child/ (2188669)
**25**  exp Infant/ (1267619)
**26**  exp Adolescent/ (2235516)
**27**  exp Young Adult/ (1021787)
**28**  (infan* or neonat* or newborn or baby or babies or child* or kid or kids or toddler* or adolescen* or teen* or boy* or girl* or juvenil* or youth* or kindergar* or puber* or pubescen* or prepubescen* or prepuberty* or pediatric* or paediatric* or peadiatric* or school* or preschool* or college).tw. (3208556)
**29**  (young adj2 adult*).tw. (132630)
**30**  (young adj2 person*).tw. (4784)
**31**  (young adj2 individual*).tw. (7290)
**32**  (young adj2 population*).tw. (8578)
**33**  (young adj2 wom#n).tw. (42968)
**34**  (young adj man).tw. (7162)
**35**  (young adj2 men).tw. (20367)
**36**  (young adj2 people*).tw. (40293)
**37**  23 or 24 or 25 or 26 or 27 or 28 or 29 or 30 or 31 or 32 or 33 or 34 or 35 or 36 (5660478)
**38**  14 and 22 and 37 (1489)

Updated search:

Ovid MEDLINE(R) ALL <1946 to September 15, 2025>

Ovid MEDLINE(R) ALL <1946 to September 26, 2025>

1 exp Renal Insufficiency, Chronic/ or exp Uremia/ or exp renal replacement therapy/ or "Chronic Kidney Disease-Mineral and Bone Disorder"/ (336293)

2 ((chronic* or progressi*) adj1 (renal* or kidney*)).tw. (129217)

3 ((kidney* or renal*) adj1 insufficien*).tw. (25420)

4 (ckd or ((kidney* or renal*) adj1 disease*)).tw. (203326)

5 ((kidney* or renal*) adj1 fail*).tw. (110470)

6 ((endstage* or end-stage* or "end stage*") adj1 (renal* or kidney*)).tw. (56488)

7 (esrd or eskd or esrf or eskf).tw. (25005)

8 ((renal or kidney) adj2 replace*).tw. (23256)

9 (hemodialys* or haemodialys* or peritoneal dialysis or RRT or KRT).tw. (123097)

10 ((kidney* or renal) adj3 (transplant* or graft* or allograft*)).tw. (116324)

11 (CAPD or APD or NIPD).tw. (13752)

12 dialys*.tw. (139720)

13 (artificial adj1 kidney*).tw. (1613)

14 1 or 2 or 3 or 4 or 5 or 6 or 7 or 8 or 9 or 10 or 11 or 12 or 13 (610224)

15 exp Exercise Test/ or exp Exercise/ (330090)

16 exp Sports/ (235900)

17 exp Physical Fitness/ or exp Physical Exertion/ (94914)

18 exp Mind-Body Therapies/ (50803)

19 exp Exercise Therapy/ (72499)

20 exp Exercise Movement Techniques/ (11865)

21 (exercis* or physical therap* or physical activity or physical inactiv* or sport or sports or movement or mobility or sedentar* or physiotherapy* or muscle training or strength training or functional therap* or kinesiotherap* or resistance training or resistance program* or physical fitness).tw. (1167733)

22 15 or 16 or 17 or 18 or 19 or 20 or 21 (1394574)

23 exp Puberty/ (19882)

24 exp Child/ (2289120)

25 exp Infant/ (1320575)

26 exp Adolescent/ (2354862)

27 exp Young Adult/ (1166996)

28 (infan* or neonat* or newborn or baby or babies or child* or kid or kids or toddler* or adolescen* or teen* or boy* or girl* or juvenil* or youth* or kindergar* or puber* or pubescen* or prepubescen* or prepuberty* or pediatric* or paediatric* or peadiatric* or school* or preschool* or college).tw. (3469307)

29 (young adj2 adult*).tw. (148147)

30 (young adj2 person*).tw. (5117)

31 (young adj2 individual*).tw. (8682)

32 (young adj2 population*).tw. (9566)

33 (young adj2 wom#n).tw.(46089)

34 (young adj man).tw.	(7578)

35 (young adj2 men).tw.	(21456)

36 (young adj2 people*).tw.(45843)

37 23 or 24 or 25 or 26 or 27 or 28 or 29 or 30 or 31 or 32 or 33 or 34 or 35 or 36	(6053543)

38 14 and 22 and 37(1606)

39 limit 38 to dt=20240215-20250912 (117)

**Database: Embase <1974 to 2024 February 16>**
**Search Strategy:**
**1**  kidney failure/ or exp chronic kidney failure/ or exp end stage renal disease/ or exp mild renal impairment/ or exp moderate renal impairment/ or exp renal replacement therapy-dependent renal disease/ or exp severe renal impairment/ or exp subclinical renal impairment/ or exp uremia/ (380262)
**2**  exp renal replacement therapy/ (242813)
**3**  exp dialysis/ (236418)
**4**  exp kidney transplantation/ (185906)
**5**  ((chronic* or progressi*) adj1 (renal* or kidney*)).tw. (179236)
**6**  ((kidney* or renal*) adj1 insufficien*).tw. (34091)
**7**  (ckd or ((kidney* or renal*) adj1 disease*)).tw. (288861)
**8**  ((kidney* or renal*) adj1 fail*).tw. (157184)
**9**  ((endstage* or end-stage* or "end stage*") adj1 (renal* or kidney*)).tw. (80103)
**10**  (esrd or eskd or esrf or eskf).tw. (43282)
**11**  ((renal or kidney) adj2 replace*).tw. (35507)
**12**  (hemodialys* or haemodialys* or peritoneal dialysis or RRT or KRT).tw. (170313)
**13**  ((kidney* or renal) adj3 (transplant* or graft* or allograft*)).tw. (172853)
**14**  (CAPD or APD or NIPD).tw. (18366)
**15**  dialys*.tw. (195297)
**16**  (artificial adj1 kidney*).tw. (1045)
**17**  1 or 2 or 3 or 4 or 5 or 6 or 7 or 8 or 9 or 10 or 11 or 12 or 13 or 14 or 15 or 16 (931160)
**18**  exp Exercise Test/ or exp Exercise/ (525673)
**19**  exp Sport/ (218054)
**20**  exp Fitness/ (43568)
**21**  exp physical activity/ (551275)
**22**  exp kinesiotherapy/ (102829)
**23**  (exercis* or physical therap* or physical activity or physical inactiv* or sport or sports or movement or mobility or sedentar* or physiotherapy* or muscle training or strength training or functional therap* or kinesiotherap* or resistance training or resistance program* or physical fitness).tw. (1348499)
**24**  18 or 19 or 20 or 21 or 22 or 23 (1851049)
**25**  exp Puberty/ (49494)
**26**  exp Child/ (3157160)
**27**  exp Adolescent/ (1815246)
**28**  exp Young Adult/ (533631)
**29**  exp school/ (404108)
**30**  (infan* or neonat* or newborn or baby or babies or child* or kid or kids or toddler* or adolescen* or teen* or boy* or girl* or juvenil* or youth* or kindergar* or puber* or pubescen* or prepubescen* or prepuberty* or pediatric* or paediatric* or peadiatric* or school* or preschool* or college).tw. (4089622)
**31**  (young adj2 adult*).tw. (174773)
**32**  (young adj2 person*).tw. (6996)
**33**  (young adj2 individual*).tw. (9920)
**34**  (young adj2 population*).tw. (12882)
**35**  (young adj2 wom#n).tw. (57192)
**36**  (young adj man).tw. (9115)
**37**  (young adj2 men).tw. (24785)
**38**  (young adj2 people*).tw. (55247)
**39**  25 or 26 or 27 or 28 or 29 or 30 or 31 or 32 or 33 or 34 or 35 or 36 or 37 or 38 (6100017)
**40**  17 and 24 and 39 (3390)

Updated search **Embase <1974 to 2024 September 15>**

1	kidney failure/ or exp chronic kidney failure/ or exp end stage renal disease/ or exp mild renal impairment/ or exp moderate renal impairment/ or exp renal replacement therapy-dependent renal disease/ or exp severe renal impairment/ or exp subclinical renal impairment/ or exp uremia/ (487912)

2	exp renal replacement therapy/ (272348)

3	exp dialysis/ (261918)

4	exp kidney transplantation/ (203857)

5	((chronic* or progressi*) adj1 (renal* or kidney*)).tw. (208849)

6	((kidney* or renal*) adj1 insufficien*).tw. (36608)

7	(ckd or ((kidney* or renal*) adj1 disease*)).tw. (337404)

8	((kidney* or renal*) adj1 fail*).tw. (170506)

9	((endstage* or end-stage* or "end stage*") adj1 (renal* or kidney*)).tw. (90516)

10	(esrd or eskd or esrf or eskf).tw. (49392)

11	((renal or kidney) adj2 replace*).tw. (42266)

12	(hemodialys* or haemodialys* or peritoneal dialysis or RRT or KRT).tw. (188123)

13	((kidney* or renal) adj3 (transplant* or graft* or allograft*)).tw. (189346)

14	(CAPD or APD or NIPD).tw. (19652)

15	dialys*.tw. (214539)

16	(artificial adj1 kidney*).tw. (1101)

17	1 or 2 or 3 or 4 or 5 or 6 or 7 or 8 or 9 or 10 or 11 or 12 or 13 or 14 or 15 or 16 (1049815)

18	exp Exercise Test/ or exp Exercise/ (627137)

19	exp Sport/ (248186)

20	exp Fitness/ (50955)

21	exp physical activity/ (648671)

22	exp kinesiotherapy/ (124177)

23	(exercis* or physical therap* or physical activity or physical inactiv* or sport or sports or movement or mobility or sedentar* or physiotherapy* or muscle training or strength training or functional therap* or kinesiotherap* or resistance training or resistance program* or physical fitness).tw. (1545250)

24	18 or 19 or 20 or 21 or 22 or 23 (2113663)

25	exp Puberty/ (58169)

26	exp Child/ (3468719)

27	exp Adolescent/ (2001950)

28	exp Young Adult/ (651868)

29	exp school/ (429882)

30	(infan* or neonat* or newborn or baby or babies or child* or kid or kids or toddler* or adolescen* or teen* or boy* or girl* or juvenil* or youth* or kindergar* or puber* or pubescen* or prepubescen* or prepuberty* or pediatric* or paediatric* or peadiatric* or school* or preschool* or college).tw. (4521340)

31	(young adj2 adult*).tw. (200693)

32	(young adj2 person*).tw. (7777)

33	(young adj2 individual*).tw. (12098)

34	(young adj2 population*).tw. (14727)

35	(young adj2 wom#n).tw. (62635)

36	(young adj man).tw. (9717)

37	(young adj2 men).tw. (26789)

38	(young adj2 people*).tw. (64329)

39	25 or 26 or 27 or 28 or 29 or 30 or 31 or 32 or 33 or 34 or 35 or 36 or 37 or 38 (6712790)

40	17 and 24 and 39 (4210)

41	limit 40 to dc=20240215-20250912 (782)

CENTRAL Cochrane Central Register of Controlled Trial search strategy

| #1 | MeSH descriptor: [Renal Insufficiency, Chronic] explode all trees | 9583 |
| --- | --- | --- |
| #2 | MeSH descriptor: [Uremia] explode all trees | 565 |
| #3 | MeSH descriptor: [Renal Replacement Therapy] explode all trees | 12572 |
| #4 | MeSH descriptor: [Chronic Kidney Disease-Mineral and Bone Disorder] explode all trees | 116 |
| #5 | ((chronic* or progressi*) NEAR/1 (renal* or kidney*)):ti,ab,kw (Word variations have been searched) | 14669 |
| #6 | ((kidney* or renal*) NEAR/1 insufficien*):ti,ab,kw (Word variations have been searched) | 7638 |
| #7 | ((kidney* or renal*) NEAR/1 disease*):ti,ab,kw | 25197 |
| #8 | ((kidney* or renal*) NEAR/1 fail*):ti,ab,kw | 22148 |
| #9 | ((endstage or end-stage or "end stage") NEAR/1 (renal* or kidney*)):ti,ab,kw | 6101 |
| #10 | (esrd or eskd or esrf or eskf):ti,ab,kw | 2829 |
| #11 | ((renal or kidney) NEAR/2 replace*):ti,ab,kw | 3328 |
| #12 | (hemodialys* or haemodialys* or peritoneal dialysis or RRT or KRT):ti,ab,kw | 17250 |
| #13 | ((kidney* or renal) NEAR/3 (transplant* or graft* or allograft*)):ti,ab,kw | 12622 |
| #14 | (CAPD or APD or NIPD):ti,ab,kw | 1218 |
| #15 | (dialys* or ckd):ti,ab,kw | 23828 |
| #16 | (artificial NEAR/1 kidney*):ti,ab,kw | 202 |
| #17 | #1 or #2 or #3 or #4 or #5 or #6 or #7 or #8 or #9 or #10 or #11 or #12 or #13 or #14 or #15 or #16 | 63188 |
| #18 | MeSH descriptor: [Exercise] explode all trees | 38282 |
| #19 | MeSH descriptor: [Sports] explode all trees | 21906 |
| #20 | MeSH descriptor: [Physical Fitness] explode all trees | 4891 |
| #21 | MeSH descriptor: [Exercise Test] explode all trees | 11002 |
| #22 | MeSH descriptor: [Physical Exertion] explode all trees | 4828 |
| #23 | MeSH descriptor: [Mind-Body Therapies] explode all trees | 9261 |
| #24 | MeSH descriptor: [Exercise Therapy] explode all trees | 21433 |
| #25 | MeSH descriptor: [Exercise Movement Techniques] explode all trees | 3480 |
| #26 | (exercis* or physical therap* or physical activity or physical inactiv* or sport or sports or movement or mobility or sedentar* or physiotherapy* or muscle training or strength training or functional therap* or weight lifting or kinesiotherap* or resistance training or resistance program* or physical fitness):ti,ab,kw | 295685 |
| #27 | (rehab* NEAR/2 physical):ti,ab,kw | 2028 |
| #28 | #18 or #19 or #20 or #21 or #22 or #23 or #24 or #25 or #26 or #27 | 303505 |
| #29 | MeSH descriptor: [Pediatrics] explode all trees | 1042 |
| #30 | MeSH descriptor: [Child] explode all trees | 81197 |
| #31 | MeSH descriptor: [Infant] explode all trees | 45750 |
| #32 | MeSH descriptor: [Adolescent] explode all trees | 136261 |
| #33 | MeSH descriptor: [Young Adult] explode all trees | 95088 |
| #34 | MeSH descriptor: [Puberty] explode all trees | 473 |
| #35 | (infan* or neonat* or newborn or baby or babies or child* or kid or kids or toddler* or adolescen* or teen* or boy* or girl* or juvenil* or youth* or kindergar* or puber* or pubescen* or prepubescen* or prepuberty* or pediatric* or paediatric* or peadiatric* or school* or preschool* or college):ti,ab,kw | 399144 |
| #36 | ((young NEAR/2 adult*)):ti,ab,kw | 116385 |
| #37 | (young NEAR/2 person*):ti,ab,kw | 845 |
| #38 | (young NEAR/2 individual*):ti,ab,kw | 667 |
| #39 | (young NEAR/2 population):ti,ab,kw | 668 |
| #40 | (young NEAR/2 women):ti,ab,kw | 3848 |
| #41 | (young NEAR/2 men):ti,ab,kw | 3469 |
| #42 | (young NEAR/2 people*):ti,ab,kw | 3568 |
| #43 | #29 or #30 or #31 or #32 or #33 or #34 or #35 or #36 or #37 or #38 or #39 or #40 or #41 or #42 | 462102 |
| #44 | #17 and #28 and #43 | 651 |

Updated search

| #1 | MeSH descriptor: [Renal Insufficiency, Chronic] explode all trees | 10056 |
| --- | --- | --- |
| #2 | MeSH descriptor: [Uremia] explode all trees | 584 |
| #3 | MeSH descriptor: [Renal Replacement Therapy] explode all trees | 12764 |
| #4 | MeSH descriptor: [Chronic Kidney Disease-Mineral and Bone Disorder] explode all trees | 118 |
| #5 | ((chronic* or progressi*) NEAR/1 (renal* or kidney*)):ti,ab,kw (Word variations have been searched) | 16446 |
| #6 | ((kidney* or renal*) NEAR/1 insufficien*):ti,ab,kw (Word variations have been searched) | 8107 |
| #7 | ((kidney* or renal*) NEAR/1 disease*):ti,ab,kw | 27733 |
| #8 | ((kidney* or renal*) NEAR/1 fail*):ti,ab,kw | 23583 |
| #9 | ((endstage or end-stage or "end stage") NEAR/1 (renal* or kidney*)):ti,ab,kw | 6631 |
| #10 | (esrd or eskd or esrf or eskf):ti,ab,kw | 3142 |
| #11 | ((renal or kidney) NEAR/2 replace*):ti,ab,kw | 3773 |
| #12 | (hemodialys* or haemodialys* or peritoneal dialysis or RRT or KRT):ti,ab,kw | 18791 |
| #13 | ((kidney* or renal) NEAR/3 (transplant* or graft* or allograft*)):ti,ab,kw | 13188 |
| #14 | (CAPD or APD or NIPD):ti,ab,kw | 1331 |
| #15 | (dialys* or ckd):ti,ab,kw | 26088 |
| #16 | (artificial NEAR/1 kidney*):ti,ab,kw | 206 |
| #17 | #1 or #2 or #3 or #4 or #5 or #6 or #7 or #8 or #9 or #10 or #11 or #12 or #13 or #14 or #15 or #16 | 68027 |
| #18 | MeSH descriptor: [Exercise] explode all trees | 40660 |
| #19 | MeSH descriptor: [Sports] explode all trees | 22999 |
| #20 | MeSH descriptor: [Physical Fitness] explode all trees | 5117 |
| #21 | MeSH descriptor: [Exercise Test] explode all trees | 110171 |
| #22 | MeSH descriptor: [Physical Exertion] explode all trees | 4850 |
| #23 | MeSH descriptor: [Mind-Body Therapies] explode all trees | 9877 |
| #24 | MeSH descriptor: [Exercise Therapy] explode all trees | 23260 |
| #25 | MeSH descriptor: [Exercise Movement Techniques] explode all trees | 3761 |
| #26 | (exercis* or physical therap* or physical activity or physical inactiv* or sport or sports or movement or mobility or sedentar* or physiotherapy* or muscle training or strength training or functional therap* or weight lifting or kinesiotherap* or resistance training or resistance program* or physical fitness):ti,ab,kw | 475359 |
| #27 | (rehab* NEAR/2 physical):ti,ab,kw | 2411 |
| #28 | #18 or #19 or #20 or #21 or #22 or #23 or #24 or #25 or #26 or #27 | 482635 |
| #29 | MeSH descriptor: [Pediatrics] explode all trees | 1067 |
| #30 | MeSH descriptor: [Child] explode all trees | 83147 |
| #31 | MeSH descriptor: [Infant] explode all trees | 46120 |
| #32 | MeSH descriptor: [Adolescent] explode all trees | 138148 |
| #33 | MeSH descriptor: [Young Adult] explode all trees | 101527 |
| #34 | MeSH descriptor: [Puberty] explode all trees | 472 |
| #35 | (infan* or neonat* or newborn or baby or babies or child* or kid or kids or toddler* or adolescen* or teen* or boy* or girl* or juvenil* or youth* or kindergar* or puber* or pubescen* or prepubescen* or prepuberty* or pediatric* or paediatric* or peadiatric* or school* or preschool* or college):ti,ab,kw | 431972 |
| #36 | ((young NEAR/2 adult*)):ti,ab,kw | 125080 |
| #37 | (young NEAR/2 person*):ti,ab,kw | 877 |
| #38 | (young NEAR/2 individual*):ti,ab,kw | 775 |
| #39 | (young NEAR/2 population):ti,ab,kw | 833 |
| #40 | (young NEAR/2 women):ti,ab,kw | 4154 |
| #41 | (young NEAR/2 men):ti,ab,kw | 3654 |
| #42 | (young NEAR/2 people*):ti,ab,kw | 4083 |
| #43 | #29 or #30 or #31 or #32 or #33 or #34 or #35 or #36 or #37 or #38 or #39 or #40 or #41 or #42 | 501215 |
| #44 | #17 and #28 and #43  with Publication Year from 2024 to 2025, with Cochrane Library publication date from Feb 2024 to Sep 2025, in Trials | 147 |

**CINAHL (EBSCO) 12 FEB 2024**

| ID | Search Terms | Results |
| --- | --- | --- |
| S1 | (MH "Exercise Test+") | 36,232 |
| S2 | (MH "Exercise+") | 130,613 |
| S3 | (MH "Sports+") | 91,199 |
| S4 | (MH "Physical Fitness+") | 21,434 |
| S5 | (MH "Mind Body Techniques+") | 47,842 |
| S6 | (MH "Therapeutic Exercise+") | 64,404 |
| S7 | (MH “Recreation+”) OR (MH “Walking+”) OR (MH "Physical Activity") | 118,398 |
| S8 | rehab* adj2 physical OR ( exercis* or physical therap* or physical activity or physical inactiv* or sport or sports or movement or mobility or sedentar* or physiotherapy* or muscle training or strength training or functional therap* or weight lifting or kinesiotherap* or resistance training or resistance program* or physical fitness ) | 552,940 |
| S9 | (MH "Renal Insufficiency, Chronic+") | 33,246 |
| S10 | (MH "Uremia+") | 2,458 |
| S11 | (MH "Renal Replacement Therapy+") | 37,652 |
| S12 | (MH "Renal Osteodystrophy") | 264 |
| S13 | (MH "Dialysis+") | 26,793 |
| S14 | ( ((chronic* or progressi*) adj1 (renal* or kidney*)) ) OR ( (kidney* or renal*) adj1 insufficien* ) OR ( ckd or ((kidney* or renal*) adj1 disease*) ) OR ( (kidney* or renal*) adj1 fail* ) OR ( (endstage* or end-stage* or "end stage*") adj1 (renal* or kidney*) ) OR ( esrd or eskd or esrf or eskf ) OR ( (renal or kidney) adj2 replace* ) OR ( hemodialys* or haemodialys* or peritoneal dialysis or RRT or KRT ) OR ( (kidney* or renal) adj3 (transplant* or graft* or allograft*) ) OR ( CAPD or APD or NIPD ) OR dialys* OR artificial adj1 kidney* | 63,519 |
| S15 | (MH "Pediatrics+") | 23,101 |
| S16 | (MH "Puberty+") | 4,918 |
| S17 | (MH "Child+") | 762,093 |
| S18 | (MH "Adolescence+") | 610,798 |
| S19 | (MH "Young Adult") | 293,562 |
| S20 | ( infan* or neonat* or newborn or baby or babies or child* or kid or kids or toddler* or adolescen* or teen* or boy* or girl* or juvenil* or youth* or kindergar* or puber* or pubescen* or prepubescen* or prepuberty* or pediatric* or paediatric* or peadiatric* or school* or preschool* or college ) OR young adj2 adult* OR young adj2 person* OR young adj2 individual* OR young adj2 population* OR young adj2 wom#n OR young adj man OR young adj2 men OR young adj2 people* | 1,744,547 |
|  | S1 OR S2 OR S3 OR S4 OR S5 OR S6 OR S7 OR S8 | 658,558 |
|  | S9 OR S10 OR S11 OR S12 OR S13 OR S14 | 82,206 |
|  | S15 OR S16 OR S17 OR S18 OR S19 OR S20 | 1,853,235 |
|  | S21 AND S22 AND S23 | 434 |

**Updated search**

|  | S21 AND S22 AND S23 | 474 |
| --- | --- | --- |
|  | EM 20240219-20250912 | 47 |

**Database: APA PsycInfo <1806 to February Week 3 2024>**
**Search Strategy:**
**1**  exp Kidney Diseases/ or exp Dialysis/ (4176)
**2**  ((chronic* or progressi*) adj1 (renal* or kidney*)).tw. (1843)
**3**  ((kidney* or renal*) adj1 insufficien*).tw. (299)
**4**  (ckd or ((kidney* or renal*) adj1 disease*)).tw. (3515)
**5**  ((kidney* or renal*) adj1 fail*).tw. (1432)
**6**  ((endstage* or end-stage* or "end stage*") adj1 (renal* or kidney*)).tw. (1424)
**7**  (esrd or eskd or esrf or eskf).tw. (631)
**8**  ((renal or kidney) adj2 replace*).tw. (191)
**9**  (hemodialys* or haemodialys* or peritoneal dialysis or RRT or KRT).tw. (2263)
**10**  ((kidney* or renal) adj3 (transplant* or graft* or allograft*)).tw. (1107)
**11**  (CAPD or APD or NIPD).tw. (1183)
**12**  dialys*.tw. (2902)
**13**  (artificial adj1 kidney*).tw. (17)
**14**  1 or 2 or 3 or 4 or 5 or 6 or 7 or 8 or 9 or 10 or 11 or 12 or 13 (9533)
**15**  exp Exercise/ (33159)
**16**  exp Sports/ (45091)
**17**  exp Physical Fitness/ (5045)
**18**  exp "sport and exercise measures"/ (539)
**19**  exp movement therapy/ (1519)
**20**  exp Mind-body therapy/ (9902)
**21**  exp Active Living/ (282)
**22**  (exercis* or physical therap* or physical activity or physical inactiv* or sport or sports or movement or mobility or sedentar* or physiotherapy* or muscle training or strength training or functional therap* or kinesiotherap* or resistance training or resistance program* or physical fitness).tw. (294171)
**23**  15 or 16 or 17 or 18 or 19 or 20 or 21 or 22 (319220)
**24**  exp Pediatrics/ (37936)
**25**  exp Puberty/ (3371)
**26**  exp Adolescent Development/ (70622)
**27**  developmental stages/ or exp early adolescence/ or exp emerging adulthood/ or exp late adolescence/ (20022)
**28**  exp Childhood Development/ (128961)
**29**  exp Child Care/ (12550)
**30**  (infan* or neonat* or newborn or baby or babies or child* or kid or kids or toddler* or adolescen* or teen* or boy* or girl* or juvenil* or youth* or kindergar* or puber* or pubescen* or prepubescen* or prepuberty* or pediatric* or paediatric* or peadiatric* or school* or preschool* or college).tw. (1595040)
**31**  (young adj2 adult*).tw. (66904)
**32**  (young adj2 person*).tw. (3794)
**33**  (young adj2 individual*).tw. (1646)
**34**  (young adj2 population*).tw. (2115)
**35**  (young adj2 wom#n).tw. (13316)
**36**  (young adj man).tw. (1626)
**37**  (young adj2 men).tw. (8027)
**38**  (young adj2 people*).tw. (39699)
**39**  24 or 25 or 26 or 27 or 28 or 29 or 30 or 31 or 32 or 33 or 34 or 35 or 36 or 37 or 38 (1654643)
**40**  14 and 23 and 39 (93)

Updated search **APA PsycInfo <1806 to September Week 2 2025>**

1	exp Kidney Diseases/ or exp Dialysis/ (4560)

2	((chronic* or progressi*) adj1 (renal* or kidney*)).tw. (2063)

3	((kidney* or renal*) adj1 insufficien*).tw. (316)

4	(ckd or ((kidney* or renal*) adj1 disease*)).tw. (3883)

5	((kidney* or renal*) adj1 fail*).tw. (1521)

6	((endstage* or end-stage* or "end stage*") adj1 (renal* or kidney*)).tw. (1518)

7	(esrd or eskd or esrf or eskf).tw. (668)

8	((renal or kidney) adj2 replace*).tw. (223)

9	(hemodialys* or haemodialys* or peritoneal dialysis or RRT or KRT).tw. (2470)

10	((kidney* or renal) adj3 (transplant* or graft* or allograft*)).tw. (1179)

11	(CAPD or APD or NIPD).tw. (1245)

12	dialys*.tw. (3064)

13	(artificial adj1 kidney*).tw. (17)

14	1 or 2 or 3 or 4 or 5 or 6 or 7 or 8 or 9 or 10 or 11 or 12 or 13 (10218)

15	exp Exercise/ (36234)

16	exp Sports/ (50211)

17	exp Physical Fitness/ (5411)

18	exp "sport and exercise measures"/ (1009)

19	exp movement therapy/ (1654)

20	exp Mind-body therapy/ (0)

21	exp Active Living/ (302)

22	(exercis* or physical therap* or physical activity or physical inactiv* or sport or sports or movement or mobility or sedentar* or physiotherapy* or muscle training or strength training or functional therap* or kinesiotherap* or resistance training or resistance program* or physical fitness).tw. (317411)

23	15 or 16 or 17 or 18 or 19 or 20 or 21 or 22 (335642)

24	exp Pediatrics/ (35795)

25	exp Puberty/ (3616)

26	exp Adolescent Development/ (77792)

27	developmental stages/ or exp early adolescence/ or exp emerging adulthood/ or exp late adolescence/ (24706)

28	exp Childhood Development/ (138908)

29	exp Child Care/ (13263)

30	(infan* or neonat* or newborn or baby or babies or child* or kid or kids or toddler* or adolescen* or teen* or boy* or girl* or juvenil* or youth* or kindergar* or puber* or pubescen* or prepubescen* or prepuberty* or pediatric* or paediatric* or peadiatric* or school* or preschool* or college).tw. (1686295)

31	(young adj2 adult*).tw. (73654)

32	(young adj2 person*).tw. (4102)

33	(young adj2 individual*).tw. (1944)

34	(young adj2 population*).tw. (2367)

35	(young adj2 wom#n).tw. (14173)

36	(young adj man).tw. (1669)

37	(young adj2 men).tw. (8453)

38	(young adj2 people*).tw. (44198)

39	25 or 26 or 27 or 28 or 29 or 30 or 31 or 32 or 33 or 34 or 35 or 36 or 37 or 38 (1746905)

40	14 and 23 and 39 (99)

41	limit 40 to up=20240212-20250912 (6)

Web of science core collection search strategy 12 Feb 2024

| Items | search | results |
| --- | --- | --- |
| 1 | **TS=((chronic* or progressi*) NEAR/1 (renal* or kidney*))** | 151,698 |
| 2 | **TS=((kidney* or renal*) NEAR/1 insufficien*)** | 23,857 |
| 3 | **TS=(ckd or ((kidney* or renal*) NEAR/1 disease*))** | 228,920 |
| 4 | **TS=((kidney* or renal*) NEAR/1 fail*)** | 126,910 |
| 5 | **TS=((endstage* or end-stage* or "end stage*") NEAR/1 (renal* or kidney*))** | 54,204 |
| 6 | **TS=(esrd or eskd or esrf or eskf)** | 26,005 |
| 7 | **TS=((renal or kidney) NEAR/2 replace*)** | 22,676 |
| 8 | **TS=(hemodialys* or haemodialys* or peritoneal dialysis or RRT or KRT)** | 148,136 |
| 9 | **TS=((kidney* or renal) NEAR/3 (transplant* or graft* or allograft*))** | 160,850 |
| 10 | **TS=(CAPD or APD or NIPD)** | 21,546 |
| 11 | **TS=(dialys*)** | 149,747 |
| 12 | **TS=(artificial NEAR/1 kidney*)** | 1,283 |
| 13 | **#12 OR #11 OR #10 OR #9 OR #8 OR #7 OR #6 OR #5 OR #4 OR #3 OR #2 OR #1** | 632,931 |
| 14 | **TS=(exercis* or physical therap* or physical activity or physical inactiv* or sport or sports or movement or mobility or sedentar* or physiotherapy* or muscle training or strength training or functional therap* or weight lifting or kinesiotherap* or resistance training or resistance program* or physical fitness)** | 2,893,434 |
| 15 | **TS=(rehab* NEAR/2 physical)** | 11,182 |
| 16 | **#15 OR #14** | 2,897,486 |
| 17 | **TS=(infan* or neonat* or newborn or baby or babies or child* or kid or kids or toddler* or adolescen* or teen* or boy* or girl* or juvenil* or youth* or kindergar* or puber* or pubescen* or prepubescen* or prepuberty* or pediatric* or paediatric* or peadiatric* or school* or preschool* or college)** | 4,729,034 |
| 18 | **TS=(young NEAR/2 adult*)** | 204,642 |
| 19 | **TS=(young NEAR/2 person*)** | 8,591 |
| 20 | **TS=(young NEAR/2 individual*)** | 17,145 |
| 21 | **TS=(young NEAR/2 population*)** | 20,642 |
| 22 | **TS=(young NEAR/2 women)** | 71,351 |
| 23 | **TS=(young NEAR/2 men)** | 43,328 |
| 24 | **TS=(young NEAR/2 people*)** | 90,007 |
| 25 | **#17 OR #18 OR #19 OR #20 OR #21 OR #22 OR #23 OR #24** | 4,954,747 |
| 26 | **#25 AND #16 AND #13** | 1937 |

Updated Search

| 26 | **#25 AND #16 AND #13**  Timespan: 2024-02-15 to 2025-09-12 (Index Date) | 239 |
| --- | --- | --- |

PEDro physiotherapy evidence database

Search date 12 February 2024

Title and abstract “kidney”

Therapy fitness training (selected)

Results: 201

Updated search

Search date 12 September 2025

**Title and abstract** “kidney”

**Therapy** fitness training (selected)

**New record added since: 12/02/2024**

Results 19

Grey literature search

ClinicalTrials.gov

Condition/disease Chronic kidney disease

Other terms Children

Intervention physical activity

All studies

| NCT Number | Study Title | Study URL | Study Status | Study Results |
| --- | --- | --- | --- | --- |
| NCT01270529 | Pedometers to Assess and Increase Physical Activity Among Children With Chronic Kidney Disease | <https://clinicaltrials.gov/study/NCT01270529> | COMPLETED | YES |
| NCT05354726 | Skeletal and Respiratory Muscle Strength, Pulmonary Function, Exercise Capacity and Physical Activity in Pediatric Chronic Kidney Disease | <https://clinicaltrials.gov/study/NCT05354726> | COMPLETED | NO |
| NCT05657236 | Effect of Strength and Conditioning Training on Functional Performance in Children With Chronic Kidney Disease | <https://clinicaltrials.gov/study/NCT05657236> | NOT_YET_RECRUITING | NO |
| NCT03991169 | Oral Iron in Children With Chronic Kidney Disease | <https://clinicaltrials.gov/study/NCT03991169> | RECRUITING | NO |
| NCT04010981 | Comparison of the Effectiveness of Virtual Reality and Video Assisted Exercises in Pediatric Chronic Kidney Disease. | <https://clinicaltrials.gov/study/NCT04010981> | COMPLETED | NO |
| NCT05970172 | A Study of Roxadustat to Treat Anemia in Children and Teenagers With Chronic Kidney Disease | <https://clinicaltrials.gov/study/NCT05970172> | RECRUITING | NO |
| NCT02803541 | Does a Mainstream Summer Camp Experience Improve Pediatric Kidney Patients' Physical Fitness and Self Concept? | <https://clinicaltrials.gov/study/NCT02803541> | COMPLETED | NO |
| NCT06655181 | Evaluating the Effectiveness of Respiratory Muscle Training in Pediatric Kidney Transplant Patient | <https://clinicaltrials.gov/study/NCT06655181> | ACTIVE_NOT_RECRUITING | NO |
| NCT05064267 | Hemostatic Profiles in Pediatric CKD | <https://clinicaltrials.gov/study/NCT05064267> | COMPLETED | NO |
| NCT01561118 | DiaSport - Endurance-orientated Training Program With Children and Adolescents on Maintenance Hemodialysis | <https://clinicaltrials.gov/study/NCT01561118> | COMPLETED | NO |
| NCT01479088 | Cinacalcet in Paediatric Secondary Hyperparathyroidism (SHPT) Due to Chronic Kidney Disease (CKD) | <https://clinicaltrials.gov/study/NCT01479088> | UNKNOWN | NO |
| NCT03999840 | Eculizumab to Cemdisiran Switch in aHUS | <https://clinicaltrials.gov/study/NCT03999840> | WITHDRAWN | NO |

Updated search new results

| NCT Number | Study Title | Study URL | Study Status | Study Results |
| --- | --- | --- | --- | --- |
| **NCT06981585** | Physical Impairments in Children With Chronic Renal Failure | <https://clinicaltrials.gov/study/NCT06981585> | RECRUITING | NO |
| **NCT06677021** | Understanding Physical Activity and Quality of Life in Children and Young People with Chronic Kidney Disease | <https://clinicaltrials.gov/study/NCT06677021> | NOT_YET_RECRUITING | NO |
| NCT07129083 | Exploring Predictors of Quality of Life in Older Adults With End-Stage Kidney Disease Receiving In-Center Hemodialysis | https://clinicaltrials.gov/study/NCT07129083 | COMPLETED | YES |
| **NCT06861816** | Ventilatory Functions and Physical Intolerance in ESRD Adolescents: Response to Program of Walking With Dogs | <https://clinicaltrials.gov/study/NCT06861816> | RECRUITING | NO |
| **NCT07162220** | The Effect of a Mobile Game Application on Children With CRF | <https://clinicaltrials.gov/study/NCT07162220> | COMPLETED | YES |
| **NCT05630326** | Association Between COVID-19 and UTIs Caused by ESBL Organism in Infants | <https://clinicaltrials.gov/study/NCT05630326> | COMPLETED | YES |
| **NCT06862466** | Impact of an Intervention Education Program on the Knowledge, Attitudes, and Practices of Dietitians | <https://clinicaltrials.gov/study/NCT06862466> | RECRUITING | No |

ISRCTN registry

Condition: chronic kidney disease

Interventions: physical activity

Results:10

| **ISRCTN** | **Title** | **Registered with ISRCTN** | **Registered with ISRCTN** | **Age group** |
| --- | --- | --- | --- | --- |
| **ISRCTN15497320** | Steroid hormone metabolism and muscle loss in chronic kidney disease | 11/04/2022 | 11/04/2022 | Adult |
| **ISRCTN71490154** | Testing a health coaching intervention for early-stage chronic kidney diseases | 31/01/2022 | 31/01/2022 | Adult |
| **ISRCTN18314195** | Online education self-management programme in people with kidney disease | 18/12/2020 | 18/12/2020 | Adult |
| **ISRCTN53557203** | Home-based resistance exercise training in chronic kidney disease | 05/03/2020 | 05/03/2020 | Adult |
| **ISRCTN12609324** | The GFR-Exercise feasibility study | 01/10/2018 | 01/10/2018 | Adult |
| **ISRCTN11596292** | The effect of exercise and the immune system on muscle wasting in chronic kidney disease | 07/11/2017 | 07/11/2017 | Adult |
| **ISRCTN18308813** | Physical activity, depression and illness perceptions in CKD | 26/09/2016 | 26/09/2016 | Adult |
| **ISRCTN87066351** | Physical activity opinions in kidney patients | 23/03/2016 | 23/03/2016 | Adult |
| **ISRCTN11615440** | Cardiovascular risk and physical condition in kidney patients | 22/01/2016 | 22/01/2016 | Adult |
| **ISRCTN69257140** | Programmes to encourage exercise and active lifestyles in kidney disease | 21/08/2015 | 21/08/2015 | Adult |

Updated search:

Urological and genital diseases; Intervention: physical activity, Chronic kidney disease

New results

| **ISRCTN** | **Title** | **Age group** |
| --- | --- | --- |
| ISRCTN84422148 | Investigating lifestyle factors in kidney disease | adults |
| ISRCTN66295470 | Effects of a personalized exercise prescription after kidney transplantation | adults |
| ISRCTN95031194 | Kidney injury after major surgery | adults |
| ISRCTN73187232 | Multifactorial Approach and Superior Treatment Efficacy in Renal Patients with the Aid of Nurse practitioners | adults |

WHO International Clinical Trials Registry Platforms

<https://trialsearch.who.int/AdvSearch.aspx>

International Society for peritoneal dialysis (<https://ispd.org/guidelines/>): Physical activity and exercise in PD: ISPD and GREX practice recommendations

National Kidney Foundation (https://www.kidney.org ) KDIGO guidelines <https://www.ajkd.org/content/kdoqiguidelines>;

UK kidney association <https://ukkidney.org/health-professionals/guidelines/guidelines-commentaries>: Clinical practice guideline exercise and lifestyle in chronic kidney disease

Supplement 2 Data Extraction Instrument and A Guidance Sheet

| Author | Eg, Smith, Smith & Hunt, Smith et al. (for more than 2 authors) |
| --- | --- |
| Title | Full title of the articles/ books/ conference articles, etc. |
| Year | The year the source is published. |
| Publication | The journal name, or organization name if it is a guideline, or website if it is a blog or other types of resources. |
| Country | The country where the study was conducted. |
| Type | Preprints  Peer-reviewed research articles  Primary research (observational study, qualitative study, health economic studies, case study, RCT, single-arm, CCT)  Evidence syntheses: Guidelines, evidence summary, reviews including umbrella reviews, narrative reviews, scoping reviews, rapid reviews, systematic reviews, etc.  Theses or expert opinions, editorials, comments, etc.  Conference materials: abstracts or slides  Website resources: blog, patient education material, etc. |
| Aim | Aim and objectives of the articles or guidelines. |
| Participants | inclusion or exclusion criteria for participants |
| Sample Size | - The number of participants is included in the study - The number of participants included in the data analysis of outcomes. - if there are more than 1 arm, state sample size for each arm. |
| Date of data collection | For primary research, extract the date of the data collection; and for the review, extract the time period for literature search. If not mentioned, NA. |
| Ages | The range and mean/median age of the study participants, if there are more than 1 arm, state ages for each arm. |
| Sex | State sex composition for each arm. |
| Ethnicity |  |
| The stage of CKD  Standard for eGFR | 1-5 CKD |
| KRT modality | Non, haemodialysis, peritoneal dialysis, transplantation, NK |
| Other demographics | Primary disease, comorbidities |
| Settings | Where the study is conducted. (Eg. Home, school, primary outpatient clinic, inpatient hospital room) |
| Levels of physical activity | What is the current level/pattern of physical activity among children and young people with CKD?  Level How it has been measured? (measurement)  To whom it compared with? Comparison  Related factors? |
| Barriers of physical activity | What factors decreasing PA levels among children with chronic kidney disease? |
| Facilitators of physical activity | What are factors are promoting PA among children with chronic kidney disease? |
| PA experience | perceived importance; feeling of participating PA |
| Type of physical activity | Aerobic, resistance, combination, mind (yoga, stretch, taichi, qigong, baduanqin, etc), sport, leisure |
| Physical activity intervention details (for experimental study or SR of interventions) | If there is an intervention conducted, extract following data^a^.   - Provided the name or a phrase that describes the intervention. - The study addressing which aspects of interventions? Safety, feasibility, or efficacy - Describe any rationale, theory, or goal of the elements essential to the intervention - Who delivers the intervention - Requirements for the intervention, publicly or privately funded care, volume of activity, details of the healthcare system, or the availability of certain facilities or equipment. - Delivery of the PA intervention: face to face or online or combined, individual or in a group,etc.. - Content of the PA intervention: Intensity, mild, moderate, moderate to vigorous, vigorous intensity; Frequency; Duration. According to ACSM, exercise prescription included the FITT-VP (frequency, intensity, time and type, volume of the exercise, progression); and limitations and contraindications for specific groups. - Adherence of PA intervention: the percentage of the interventional group adhere to the exercise protocol. - If the intervention is personalized, describe what, why, when and how the intervention is adapted. - If the intervention was modified during the course of the study, what why when and how it is adjusted. |
| Outcomes (health-related)  (retrospective or prospective cohort study, or interventional study or SR) | - What are the outcomes have been tested in terms of feasibility (pilot) or effectiveness? Patient-reported outcome or objectively-measure outcome? - How the outcomes measured: Patient reported or objectively measured, extract the scales involved. - When and frequency of the outcomes measurements. |
| Implication | Does the author discuss the research or practice implication for physical activity among children and young adults with chronic kidney disease?  If so, what are these implications? |
