# Supplementary material for: Physical activity among children and young people with chronic kidney diseases: a scoping review
Source: Pediatr Nephrol. 2026 Apr 6;41(11):3713–47. doi: 10.1007/s00467-025-07142-0 (PMC13612716; doi:10.1007/s00467-025-07142-0)
Supplement: Supplementary file 2 [file 467_2025_7142_MOESM2_ESM.docx]

Supplementary Table 3 levels of physical activity among children and young people with chronic kidney disease

**Types of PA**

Levels of sports participation

Transplant

- Thirty-one percent of 101 children and young people with kidney transplants played organized sports during the previous year, reported by children and their families using self-developed questions[39].
- The most played sports were basketball (31%), baseball (10%), soccer (9%), cheerleading (8%) among 101 children with transplants[39].
- Eleven out of 23 (47.8%) children with transplants regularly participated in sports assessed using a semi-structured interview[26]. Four (17.4%) reported playing sports everyday; one (4.3%) reported playing sports 4-5 times a week; 4 (17.4%) playing sports 2–3 times a week; 2 (8.6%) playing sports on weekly basis[26].
- In 20 children with transplants and 33 healthy controls, soccer and badminton were reported less frequently by transplant recipients whereas activities like tag, ﬂoor hockey and baseball were reported more commonly by the transplant recipients[27].
- Forty-eight out of 135 children (36%) with transplants played no sports. Compared with CKD1-2, the incidence rate ratio (IRR) (95%CI) for sports participation amongst transplant recipients was 0.75 (0.58–0.96), adjusted for socioeconomic status and ethnicity (P=0.04). Participation in football/rugby was higher amongst children with CKD 1–2 (52%) and CKD 3–5 (28%) than children treated with dialysis (7%) and with kidney transplants (13%)[13].
- Mean minutes of sport activities per week from 48 children with transplants was 129(95%CI 97,162), assessed by the “Motorik–Modul” PA questionnaire. Those transplant patients also engaged fewer **minutes of sports activities** **across the sports settings** compared with healthy children cohort**.** (school, clubs, and other unorganized sports) and ages groups. The biggest discrepancy was in sports clubs. And there was no overlap for the total amount of sport between our study population and the control cohort for all age groups. Twenty-six percentage of total time of moderate to vigorous activity per day was spent on sport activities mainly performed during school (51% sport activities in school, 26% sport activities in sport clubs, 23% other unorganized sport activities)[29].

Non-KRT CKD

- In 388 children with non KRT-CKD, median number of sports team played in the 12 months: 0 (0,2) [46].
- In a sub cohort of 136 children with CKD1-4, 78(57%) CKD reported sports team participation, and a year later, the percentage dropped to 65(48%) (P=0.02) using Youth Risk Behaviour Survey. The median number of sports teams played on in the past 12 months was 1(0,2)[14].
- Twenty percentage of CKD1-2 and CKD 3-5 played two or more types of sports, 7% of children on dialysis played two or more types of sports[13].

CKD of all stages (and discrete data by CKD1-2, CKD3-5, transplant, and dialysis)

- Overall, 62% out of 377 participants played sport, 48 out of 135 children (36%) with transplants played no sports, 21 out of 43 (50%) children on dialysis played no sports, 23 out of 92(25%) CKD 3–5 played no sports, and 28 out of 107(27%) CKD 1–2 played no sports, reported by participants and their caregivers through questions. Compared with CKD1-2, the incidence rate ratio (IRR) (95%CI) for sports participation amongst children with CKD 3-5, dialysis, and transplant were 0.84 (0.64–1.09), 0.59 (0.39–0.90), and 0.75 (0.58–0.96), respectively, adjusted for socioeconomic status and ethnicity (P=0.04)[13].
- The most common sports: swimming (17%), soccer (17%), football/ rugby (12%), dance (9%), and basketball (8%) among 377 children with CKD 1-5 including KRT. Participation in football/rugby was higher amongst children with CKD 1–2 (52%) and CKD 3–5 (28%) than children treated with dialysis (7%) and with kidney transplants (13%)[13].

Haemodialysis

- In a non-randomized controlled trial, 106 children were on HD and 71 children were on HDF. At 12 months, 44% children on HDF playing sports compared with only 13% of children on HD (P<0.01)[59].

Dialysis

- Twenty-one out of 43 (50%) children on dialysis played no sports, Compared with CKD1-2, the incidence rate ratio (IRR) (95%CI) for sports participation amongst children with on dialysis was 0.59 (0.39–0.90), adjusted for socioeconomic status and ethnicity (P=0.04)[13].

Levels of general exercise participation

Transplant

- Ten (50%) children with transplants reported exercising more than 3 hours/week using PAQ and 10(50%) reported less than 3 hours of exercise per week[31]
- Six (37.5%) children with transplants reported exercising more than 3 hours/week using PAQ and 10 (62.5%) children with transplants reported less than 3 hours of exercise per week[32].
- Four out of 10 (40%) children with transplants reported participating in exercise in the last one year through questions. Each exercise session lasts from 30 to 60 minutes, 1-3 days per week in the last 6 months[33].

Non-KRT CKD

- From 639 visits by 388 children with CKD1-4, median (IQR) number of days with more than 60 mins of exercise over the past 7 days was 5 (3,8) measuring by Youth Risk Behaviour Survey[46].
- From 224 children with CKD1-4, median (IQR) number of days with more than 60 mins of exercise over the past 7 days was 3 (0, 5), median (IQR) number of days with more than 20 mins of exercise over the past 7 days was 3(1, 5), and median (IQR) number of days with more than 30 mins of exercise over the past 7 days was 2 (1,5) using Youth Risk Behaviour Survey[14].

Dialysis (haemodialysis)

- Seven (23.3%) haemodialysis participants reported practicing exercise regularly measuring by The Child Behaviour Checklist and through interviewing with main caregivers[21].

Levels of general PA participation

Transplant

- Nineteen out of 38 (50%) transplant recipients reported no regular physical activity compared to 0 out of 17 healthy controls reported no regular PA (P<0.0001)[23].
- Mean minutes of physical activity per week was 107.3± 126.8 among transplants and 488.2±640.7 in healthy controls, P=0.005[27].
- In 101 transplant recipients with median (IQR) of time with transplant of 4.3 (2.4 – 6.6) years, 67% reported an increase in PA level after transplant; 13% reported less in activity and 20% reported no change in activity despite that limits on activity were more likely after transplant (68%) than before transplant (49%)[39].

Non-KRT CKD

- In 224 children with CKD 1-4, using Youth Risk Behaviour Survey, 30 (13.4%) met PA recommendation of 60 mins of daily physical activity, lower than 24.8% among 510 healthy controls (P < 0.001); 13.3% females with CKD met PA recommendations, lower than 22.5% among 251 female healthy controls (P=0.04); 13.4% males with CKD met PA recommendations, lower than 27.0% among 259 male healthy controls (P< 0.001). There was no significant difference between males and females meeting physical activity recommendations within either the CKD group or healthy cohorts[14].

CKD of all stages

- In 40 children with CKD1-5 including children on dialysis and with transplants, 34(85%) did not practice any physical activity and were sedentary. Six (15%) reported to participate regular physical activity (≤2 h/week)[11] using Habitual Level of Physical Activity (HLPA) questionnaire.

CKD3-5 and dialysis

- In 18 children CKD 3-5 and dialysis, energy expenditure on PA was comparable with 20 healthy controls, adjusted for age, gender, weight, and height[17].

**Settings of PA**

Levels of physical activity in school setting

Transplant

- Forty-eight percent of 25 children with transplants participated in physical education[30].
- Twenty-four (75%) of 32 children with transplants engaged in the physical education class[25].
- Twelve (60%) of 20 children with transplants were very active in school physical education classes, similar to 29 (87%) of 33 healthy controls which were very active in school physical education classes, (P=0.084)[27].
- Forty-eight percent of 25 children with transplants and 20% of 15 children on dialysis participated in physical education[18].
- In 48 transplant recipients with 489 ± 435 mins moderate of vigorous activity per day, 26% of total time was spent on sport activities which mainly were performed during school[29].

Non-KRT CKD

- In 224 children with CKD1-4, median days of participating physical education per week was 2 (0,5) and median (IQR) number of minutes exercising in the average physical education class 4(3,5) mins[14].

Dialysis

- Forty-eight percent of 25 children with transplants and 20% of 15 children on dialysis participated in physical education[18].

Levels of PA in non-school time

Transplant

- Among 25 children with transplants, only 9.6% - 10% after school time was spent on PA. The majority of time was spent in sedentary activities (television, computer) and self-care tasks (eating, bathing)[30].
- In 40 children on KRT, <10% non-school time were spent on participating in physical activity. This number was 9.6% in 25 transplant recipients and 7% in 15 dialysis participants[18].
- Twenty children with transplants and 33 healthy controls have similar frequency performed physical activity outside school measured by the first question in PAQ[27].

Non-KRT CKD

- Median (IQR) hours of physical activity per day using an epidemiologic questionnaire to assess past year non-school time physical activity was 1.81 (1.17,2.94) hours in 401 healthy controls. It was similar with 1.91 (1.30, 2.87) hours in 64 children CKD 2 to 3 (P=0.3), similar with 1.52 (0.78, 2.52) hours in 34 children on dialysis (P=0.2), and higher than 1.41 (0.53, 2.67) in 45 children with CKD 4 to 5 (P=0.03)[16].

Haemodialysis

- In 30 children on haemodialysis, 0 children participated exercise at school, with 3 (10.0%) exercised at home and 4 (13.3) exercised at street regularly[21].

Dialysis

- In 40 children on KRT, <10% non-school time were spent on participating in physical activity. This number was 9.6% in 25 transplant recipients and 7% in 15 dialysis participants[18].
- Median (IQR) hours of physical activity per day using an epidemiologic questionnaire to assess past year non-school time physical activity was 1.52 (0.78, 2.52) hours in 34 children on dialysis (P=0.2) [16].

**Intensity of PA**

Overall intensity

Transplant

- Among 25 children with transplants, the average MET level of activity over the 3 days was 1.8 METs. The highest level of activity reported averaged 4 METs. PA types and durations were recalled by the previous day physical activity recall (PDPAR)[30].
- In 22 children with transplants, the median METs level per 24h was 9.6, ranging from 7 to 15.5 METs. PA types and duration were assessed by Physical Activity Recall Questionnaire[34].
- In 25 transplant recipients, average METs over 3 days (non-school time) was 1.8±0.63 with the highest level of activity was 3.5±2.3. In 15 dialysis participants, average METs over 3 days (non-school time) was 1.9±1.1. The highest level of activity was 3.9± 2.9. PA types and durations were recalled by PDPAR[18].

Dialysis

- In 15 dialysis participants, average METs over 3 days (non-school time) was 1.9±1.1. The highest level of activity was 3.9± 2.9. The average METs of activities were calculated using the previous day physical activity recall (PDPAR). PA types and durations were recalled by PDPAR[18].

Moderate to vigorous PA

Transplant

- Forty-eight children with transplants reported by “Motorik–Modul” Physical Activity Questionnaire (MoMo-PAQ), 23 (48%) met the recommendation of 60 min moderate to vigorous PA per day. Twenty-six percentage of moderate to vigorous PA was spent on sport activities. These sport activities were mainly performed during school (51%), 26% in sport clubs, 23% other unorganized sport activities[29].
- In 38 children with transplants reporting with the International Physical Activity Questionnaire (IPAQ), 12 (31.6%) transplant recipients performed 1-2h per week moderate to vigorous physical activity, 7 (18.4%) transplant recipients reported ≥3 h per w eek moderate to vigorous physical activity. In 17 healthy controls, 8 (47.1%) performed 1-2h per week moderate to vigorous physical activity and 9 (52.9%) performed ≥3 h per week moderate to vigorous physical activity. The distribution of the intensity of PA among these two groups were different (P= 0.0003)[23].
- In 48 children with transplants, mean minutes of moderate to vigorous PA per week were 489 ± 435 mins measured by “Motorik– Modul” PA questionnaire. Eight-eight percentage of the amount of moderate and vigorous activity was moderate physical activity. Mean minutes of moderate PA per week were 431 ± 430 mins[29].
- In 48 children with transplants, 74% of moderate to vigorous PA was performed as everyday activities with mainly moderate intensity (moderate PA 356 ± 421 min and vigorous PA 7 ± 37 min), twenty-six percentage of moderate to vigorous PA was spent on sport activities[29].
- In 22 children with transplants, 19(86%) reported less than 60 min of daily moderate to vigorous PA, with median 26 (0–81) mins. [34].
- In 16 transplant recipients measured by accelerometers, 3(19%) accumulated ≥1h moderate to vigorous PA 1 out of the 3 days. Mean time of moderate to vigorous PA measured by accelerometery in 14 patients was 29.3±24.6 min/day. 58.5% of their time was spent in sedentary activities, 37% of time on light activity and 4.5% of time on moderate to vigorous PA[24].

Non-KRT CKD

- In 388 children with CKD 1-5 reporting with Youth Risk Behaviour Survey, median (IQR) number of days with more than 20 mins of sweating or breathing hard exercise over the past 7 days was 3 (1,5); median (IQR) number of days with more than 30 min non-sweating/breathing hard exercise was 4 (2,6)[46].
- In 60 children with CKD 2-5, 30 (50%) reported no PA beyond baseline activities of daily living or less than 150 minutes/week of moderate-intensity PA. 30(50%) were “Active”, with ≥150 minutes/week of moderate to vigorous intensity activity. PA duration at different intensity levels were assessed during an interview[15].

Vigorous PA

Transplant

- In 48 children with transplants, mean minutes of vigorous PA per week were 58±95 mins measured by “Motorik– Modul” PA questionnaire. Twenty-six (54%) didn’t practice vigorous PA. Vigorous PA was mostly practiced during sport activities[29].

Non-KRT CKD

- In 388 children with CKD 1-5 reporting with Youth Risk Behaviour Survey, median (IQR) number of days with vigorous activity was 1 (0,4) and percentage of patients with vigorous activity for more than 10 min was 353 (54%)[46].

**Steps**

Transplant

- Steps were measured by pedometers for 3 days in 20 children with transplants. Mean steps per day were 9282 ± 4666, similar with the mean steps per day among 33 healthy controls of steps of 11449±4638, P=0.124. In total, 10% of transplant recipients and 25% of healthy controls met the sex cutoﬀs for steps/day (12 000 for females and 15 000 for males). The percentages of participants meeting the sex cutoﬀs for steps per day between two groups were similar, P>0.05[27].
- The median (IQR) step counts per day in 25 transplant recipients were 8175 (4174 to 11798)[12].
- Steps were measured by accelerometery in 14 children with transplants. Mean steps per day were 7,494.8±4,428.8 and ranged from 1,17 to 19,646.7. Fourteen out of 16 were below the recommended daily step[24].
- Steps were measured by accelerometers for 7 days in 23 children with transplants. Mean steps per day were 7317 ± 3406[29].
- Steps were measured by Sensewear armband monitor in 20 children with transplants and 33 healthy controls, average steps per day was 7007 on the average 15.3 hours in transplant recipients lower than the 10305 steps per day in healthy controls on the average 15 hours, P=0.001[37].

Non-KRT CKD

- Steps were measured by pedometers for 7 days. In 16 children with CKD 1-5, the mean daily steps per day were 4290.5±1820.4, with 4788.12 ± 1919.61 in one group and 3792.87±1362.74 in other group[54].
- In early stage CKD, adolescents had median daily steps of approximately 7000, which increased to 9000 after 6 months of group-based care (assuming 14 waking hours)[61].
- The median (IQR) step counts per day in 12 children with CKD stage 1-4 was 4978 (3058, 6651)[12].

CKD of all stages (with discrete data by CKD 1-4, dialysis, and transplant)

- Steps were measured by pedometers over 7 days. In 44 children with CKD 1-5 including KRT, median (IQR) steps per day over 7 days were 5976 (3309, 8595), 44% lower than healthy young people. 90% of the males and 95% of the females was below the recommended levels of 15000 steps per day for males and 12,000 steps per day for female). 22 boys with CKD recorded 9122 (6547, 11,951) steps compared with 22 Girls with CKD recorded 4027 (2682, 5667) steps (P<0.001). Participants aged 18-20 were less active than younger participants (P<0.05)[12].
- Steps were measured by pedometers over 7 days. In 44 children with CKD 1-5 including KRT, the mean daily step counts per day were 6218 (95%CI 3637 to 9828). The median (IQR) step counts per day in 25 transplant recipients were 8175 (4174 to 11798), 4978 (3058, 6651) in 12 children with CKD stage 1-4 and 4566 (3500, 9867) in 7 children on dialysis. The differences in steps of different CKD stages were consistent with chance (P=0.10) [12].

Dialysis

- The median (IQR) step counts per day in 12 children with CKD stage 1-4 and 4566 (3500, 9867) in 7 children on dialysis[12].

**Overall activeness**

PAQ

Transplant

- In 101 children with transplants, the median score was 2.2 (IQR 1.6, 2.6). In 59 children with the mean age 11.5±2.5, median PAQ score was 2.4 (IQR 1.9, 2.9); lower than healthy children in US with a median PAQ of 3.2 (P<0.001). In 42 adolescents with the mean age 18±1.9, median PAQ score was 1.8 (IQR 1.3, 2.3); lower than healthy adolescents in US with a median PAQ of 2.8 (P<0.001). PAQ scores was higher in elementary/middle schoolers compared with high school/college. The difference was -0.48 (-0.76, 0.21) P<0.001. The older age (year) was the predictor of the PAQ scores with the estimate -0.081 (-0.11, -0.048), P=0.0006[39].
- In 27 children with transplants, median (IQR) was 2.4 (1.4–3.0)[42].
- In 29 children with transplants, median (IQR) was 2.1 (1.6–2.8)[43].
- In 20 children with transplants, mean PAQ summary score was 2.8± 0.8 compared with 3.1±0.7 in 33 healthy controls. PAQ scores were similar between two groups(P=0.180). The PAQ score was negatively correlated with age (r=-0.46, P =0.04) in the transplant[27].
- In 15 children with transplants, mean score was 2.17 ± 0.68. No respondent stated that they ran and played hard most of the time, or at recess and at the lunch time[35].
- In 11 children with transplants, the mean score was 2.16 ± 0.75. Mean score of question 2 was 2.82 ± 1.25 and mean score of question 8 was 2.27±1.19[36].
- In 17 children with transplants, mean score of PAQ was 2.4 ± 0.9[41].

Dialysis

- Mean scores of PAQ in children with dialysis and 8 aged matched healthy controls were 2.5±0.7 and 2.8±0.6. Both are moderate level of PA, with no significant difference in the groups’ activity score(P>0.05)[19].

Sub-domain Physical Activity of The Child Health and Illness Profile-Adolescent Edition (CHIP-AE)

Transplant

- Mean score was 19.3 ± 4.9 in 39 children with non-KRT CKD, 14.2 ± 3.3 in 21 children on dialysis, and 17.0 ± 4.2 in 53 children with transplant. Compared with non-KRT group, children on dialysis (p<0.05) and with transplant (p<0.05) reported less physical activity. Transplant group reported more physical activity than dialysis group (p<0.05)[10].

Non-KRT CKD

- Mean score was 19.3 ± 4.9 in 39 children with non-KRT CKD, 14.2 ± 3.3 in 21 children on dialysis, and 17.0 ± 4.2 in 53 children with transplant. Compared with non-KRT group, children on dialysis (p<0.05) and with transplant (p<0.05) reported less physical activity. Transplant group reported more physical activity than dialysis group (p<0.05)[10].

CKD of all stages (with discrete data by CKD 1-5, dialysis, and transplant)

- Compared with the score of 20.7 ± 4.7 in a national sample of 226 age-matched healthy adolescents, 113 adolescents with CKD 1-5 including KRT reported being less physically active with score of 17.3 ± 4.6 (P<0.001). Mean score was 19.3 ± 4.9 in 39 children with non-KRT CKD, 14.2 ± 3.3 in 21 children on dialysis, and 17.0 ± 4.2 in 53 children with transplant. Compared with non-KRT group, children on dialysis (p<0.05) and with transplant (p<0.05) reported less physical activity. Transplant group reported more physical activity than dialysis group (p<0.05)[10].

Dialysis

- Mean score was 19.3 ± 4.9 in 39 children with non-KRT CKD, 14.2 ± 3.3 in 21 children on dialysis, and 17.0 ± 4.2 in 53 children with transplant. Compared with non-KRT group, children on dialysis (p<0.05) and with transplant (p<0.05) reported less physical activity. Transplant group reported more physical activity than dialysis group (p<0.05)[10].

PROMIS Parent Proxy Physical Activity Items

Non-KRT CKD

- In children with CKD 2-4 with anaemia, scores from PROMIS Parent Proxy Physical Activity Items ranged from 43.8±9.6 to 45.0±6.9[58].

**Inactivity**

Percentage of physical inactivity

Transplant

- In 48 children with transplant, 29% indicated an inactive lifestyle less than 120 min/week of moderate to vigorous intensity PA[29].

Non-KRT CKD

- In 60 children with CKD 2-5 (not on KRT), 30 (50%) children were identified as Inactive with no PA beyond baseline activities of daily living or less than 150 minutes/week of moderate-intensity PA. Frequency of physical inactivity among patients was two-fold higher than 52 healthy controls (50% vs. 25%). The prevalence of physical inactivity was also increased as CKD developed from 3 to 5[15].

**Sedentary behaviour**

Sedentary time

Transplant

- In 16 children with transplant, 58.5% of their time was spent in sedentary activity, with the mean of 476.1 ± 174.3 min/day[24].

Screen time

Non-KRT CKD

- In 224 children with CKD 1-4 and 510 healthy controls, 4 (1.8%) children with CKD met screen time recommendations, lower than 27.0% of healthy controls who met the recommendations of limiting the total amount of entertainment screen time to ≤2 h per day (P<0.001). In females, 1.1% of children with CKD met the screen time recommendations, lower than 29.0% healthy females children (P<0.001). In males, 2.2% of children with CKD met screen time recommendations compared with 25% of healthy males[14].
- In 21 children with non-KRT CKD, mean hours of screen time per week was 10.1 ± 5.1 hours per week pre lockdown and 26.0 ± 10.5 after lockdown[40].

**Levels of PA compared to healthy controls**

PA participation (Percentage, Minutes per day)

Transplant

- Nineteen out 38 (50%) transplant recipients reported no regular physical activity compared to 0 out of 17 healthy controls reported no regular PA (P<0.0001)[23].

Non-KRT CKD

- Thirty (13.4%) CKD met PA 2008 recommendation, which was **lower** than that of 24.8% in 510 healthy controls (P < 0.001)[14].
- Thirty (50%) children with non-KRT CKD reported no PA beyond baseline activities of daily living or less than 150 minutes/week of moderate-intensity PA. The frequency of physical inactivity among patients was two-fold higher than 52 healthy controls (50% vs. 25%)[15].

Non-KRT and dialysis

- Compared with 401 healthy control (1.81, IQR 1.17 to 2.94), median PA hours per day was comparable in 64 children with CKD 2-3 (1.91, IQR 1.30 to 2.87, P=0.3) and 34 children on dialysis (1.52, IQR 0.78 to 2.52, P=0.2), and higher than 45 children with CKD 4-5 (1.41 IQR 0.53 to 2.67, P=0.03)[16].
- In 18 children CKD 3-5 and dialysis, energy expenditure on PA was comparable with 20 healthy controls, adjusted for age, gender, weight, and height[17].

Sports participation

Transplant

- Mean self-reported sport activities time per week was 129 (95%CI, 97 to 162) mins in 48 transplant participants. Compared to the healthy German healthy cohort (242 min/week; 95%CI, 230 to 253), transplant recipients specifically lacked engagement in sport activities[29].

Steps counts

Transplant

- Twenty transplant recipients did 229 steps less than 33 age-matched healthy controls (P=0.001)[37].
- Twenty transplant recipients (9282±4666) and 33 healthy controls (11449±4638) recorded **comparable** steps per day in 7 days by pedometers. In total, 10% and 25% of transplant recipients and healthy controls met the gender cutoffs for steps/day (12000 for females and 15000 for males). The difference was consistent with chance[27].

Non-KRT CKD

- Median (IQR) steps per day over 7 days in 44 children with CKD 1-5: 5976 (3309, 8595). Physical activity was 44% lower than healthy young people[12].

Overall activeness

Transplant

- Median PAQ score in 59 children aged 11.5±2.5 years was 2.4 (IQR 1.9, 2.9), lower than healthy children in US with a median PAQ of 3.2 (P<0.001). Median PAQ score in 42 adolescents aged 18±1.9 years was 1.8 (IQR 1.3, 2.3), lower than healthy adolescents in US with a median PAQ of 2.8 (P<0.001)[39].
- Mean PAQ score was 2.8±0.8 in 20 transplant recipients and 3.1±0.7 in 33 healthy controls. The difference was consistent with chance[27].

CKD of all stages

- Compared with a national sample of 226 age-matched healthy adolescents, 113 adolescents with CKD reported being less physically active. Scores of Subdomain physical activity from the Child Health and Illness Profile-Adolescent Edition (CHIP-AE) in children with CKD of all stages were 17.3 ± 4.6 VS 20.7 ± 4.7 in age-matched healthy controls (P<0.001)[10].

Dialysis

- Mean PAQ score was 2.5±0.7 in 14 children with dialysis and was 2.8±0.6 in 8 age matched healthy controls. Both are moderate PA. The difference of mean PAQ score between two groups was consistent with chance (P>0.05)[19].

PA frequency

Transplant

- PA frequency ≥ 3 times per week was comparable in 20 transplant recipients and 33 healthy controls[27].

Physical education participation

Transplant

- Twelve out of 20 (60%) of transplant recipients reported being very active in school physical education classes, which is comparable to that of 29 out of 33 (87%) in healthy controls (P=0.084)[27].

Screen time

Non-KRT CKD

- Four (1.8%) children with CKD met screen time recommendations, lower than that of 27.0% in 510 healthy controls (P<0.001)[14].

**Levels of PA by CKD stages**

CKD1-2 VS CKD3-5

- In children with non-KRT CKD, prevalence of physical inactivity (no PA beyond baseline activities of daily living or less than 150 minutes/week of moderate-intensity PA) increased from CKD stages 3 to 5[15]. (no detail number provided)
- Compared with 107 children with CKD1-2, 92 children with CKD 3-5 reported a similar level of sports participation, with the incidence rate ratio (IRR) of 0.84 (95% CI, 0.64 to 1.09)[13].

KRT VS non-KRT CKD

- Thirty-nine children with non-KRT CKD reported a higher score of the Child Health and Illness Profile-Adolescent Edition (19.3 ± 4.9) than 21 children on dialysis (14.2 ± 3.3, P<0.05) and 53 children with a transplant (17.0 ± 4.2, P<0.05)[10].
- Compared with 107 children with CKD 1-2, 43 children on dialysis and with transplant reported a lower level of sports participation with IRR of 0.59 (95% CI, 0.39 to 0.90) and 0.75 (95%CI, 0.58 to 0.96)[13].
- Median steps per day over 7 days, measured by pedometers, were similar across 25 children with a transplant (8175, IQR 4174 to 11798), 12 children with CKD 1-4 (4978, IQR 3058, 6651), and 7 children on dialysis (4566 IQR 3500, 9867), P=0.10[12].

Transplant VS Dialysis

- Fifteen children on established dialysis and 25 children with a transplant more than 3 months reported comparable average PA participation in the 3 day from 3 to 11 pm in terms of percentage of physical activity in non-school time (8.7% in dialysis group and 9.6% transplant group) and the average MET level of activity over the 3 days (non-school time) (1.9 in dialysis group and 1.8 in transplant group)[18].
- Fifty-three transplant recipients had a higher score of overall activeness using the Child Health and Illness Profile-Adolescent Edition (17.0 ± 4.2) than 21 children on dialysis (14.2 ± 3.3, P<0.05)[10].

HD VS HDF

- Children on HDF for 12 months reported a higher sport participation compared with those on HD in a non-randomised controlled trial (RCT)[59].

eGFR

•	PAEE with or without adjustments for age, gender, weight, and height did not differ between the groups and was not related to eGFR[17].

**Levels of PA by sex and age**

Sex

CKD of all stages

- Pedometers in 22 boys recorded a higher median steps/day than girls (9122 (IQR 6547, 11951) Vs 4027(IQR 2682, 8595), P<0.001)[12].

Non-KRT CKD

- One hundred thirty-four adolescent boys and ninety adolescent girls with CKD 1-4 reported a similar percentage of meeting physical activity recommendations (OR=1.00, 95% CI 0.42-2.35, P=0.98)[14].

Age

Transplant

- In 101 children with transplants, higher mean scores of PAQ were found in elementary/middle schoolers compared with high school/colleges, the difference was -0.48 (-0.76, 0.21), P<0.001[39]. The older age was a predictor of lower PAQ scores with estimates = -0.081 (-0.11,-0.048, P = 0.0006)[39].
- The PAQ score was negatively correlated with age (r= -0.46, P = 0.04) in 20 children with transplants[27].

CKD of all stages

- Steps per day was 44% lower among young adults (18-20 years) than younger participants, P <0.05; There was an age-related gradient in steps per day (-310, 95%CI -575 to -45, P=0.03)[12].

**Levels of PA over time**

Transplant

- In nine children undergoing transplantation, PA participation dropped 3 months post-transplantation[18].
- Among 101 children after transplantation, 67% participants reported an increase in PA level after transplant[39].

Non-KRT CKD

- Percentage of meeting PA recommendations (14.7% to 7.5%) and sports participation (57% to 48%) decreased within 1 year in 136 children with CKD 1-4 (P=0.03)[14].
- PA hours fell (7.9±4.5 to 3.7±4.1 hours/week) and mean screen time increased (10.1±5.1 to 26±10.5 hours/week) in 21 children with CKD 1-2 six months after COVID-associated lockdown[40].

Supplement Table 4 outcomes related to the levels of physical activity among children and young people with chronic kidney disease

**Cardiorespiratory Fitness/Function**

VO_2_max

Transplant

- 10 children with transplants exercising more than 3 hours/week had a higher VO_2_max/kg (ml/min/kg) measured by a maximal incremental exercise on a treadmill than those who exercising less than 3 hours/week, 28.99±1.15 VS 23.22 ±1.23; P <0.003[31].
- 10 children with transplants exercising more than 3hours per/week had a higher VO_2_max/kg ml/min/kg than 10 children with transplants exercising less than 3 hours/week, 29.59 ± 4.28 VS 24.79 ± 1.99 P = 0.0048[32].
- There were tendencies for VO2max and peak power peak output to be increased in subjects with a higher physical activity level in 20 children with transplants[37]. (no details offered)
- VO_2_max in 22 children with transplants was significantly correlated with moderate to vigorous PA (r=0.49, P=0.03)[34].

Six-minute walking distance (6MWD)

CKD of all stages

- Higher steps per day measured for 7 days using pedometers were related to higher 6MWD in 44 children with CKD of all stages. Adjusted for age, sex, and CKD categories, steps increased per 100 and 6MWD increased 1.5 (1.1-2.0) meters, P <0.001[12].

Resting heart rate

Transplant

- Amounts of MVPA or MPA measured by motorik–modul PA questionnaire had no significant effect on rest heart rate (P=0.18) in 48 children with transplants[29];
- Among 48 children with transplants vigorous physical activity VPA measured by motorik – modul PA questionnaire, 60mins VPA predicted a decrease of a resting heart rate by 3 beats/min after adjusted for sex, age and eGFR, (P=0.01)[29].

Left heart function

Transplant

- Among 48 children with transplants, MVPA was significantly associated with a higher mitral annular e’ (P= 0.01) and lower E/e´ ratio (P=0.01), indicating better left ventricular compliance and less left ventricular filling pressures[29].
- Among 48 children with transplants, MVPA was significantly associated with a decrease of mitral A velocity, A-wave (multivariable analysis) (indicating a larger contribution of atrial contraction to left ventricular filling) (P= 0.02)[29].
- 10 children with transplants who exercised more than 3 hours/week had a lower left ventricular mass compared with 10 children exercising less than 3 hours per week, 42.40 ± 19.70 g/m^2^ VS 66.83 ± 21.59 g/m^2^, P=0.038[32].

Maximum respiratory rate

Transplant

- 10 children with transplants exercising more than 3 hours/week had a similar maximum respiratory rate compared with 10 participants exercising less than 3 hours/week; 41.48 ± 8.41 VS 40.20 ± 6.3 (P>0.05)[32].

**Grip strength**

Grip strength z score

Non-KRT CKD

- From 639 person-visit from 388 children with CKD 1~4, grip strength z score increases with the number of days with more than 60 mins of exercise; the mean effect 0.06 95%CI 0.02 P= 0.008[46].
- From 639 person-visit from 388 children with CKD 1~4, grip strength z score increases with the number of days with vigorous activity; the mean effect 0.06 95%CI 0.02 P=0.008[46].
- From 639 person-visit from 388 children with CKD 1~4, grip strength z score increases with the practice of vigorous activity more than 10 mins; the mean effect 0.36 95% CI 0.02 P=0.0002[46].

Grip strength

Transplant

- Among 20 children with transplants, there was a tendency for a greater handgrip strength in moderately active subjects. (no details provided)[37].

**Body composition**

Waist circumference

Transplants

- Among 20 children with transplants, higher PAQ scores was related to the lower waist circumference (r=-0.42, P=0.036)[27].
- Among 20 children with transplants, there is insufficient evidence to find the correlation between waist circumference and total exercise time[27]. (no details provided)

BMI

Transplants

- The correlations between BMI and total exercise time, steps per day or PAQ scores were not found in 20 children with transplants[27]. (no figures or tests provided)

CKD of all stages

- One unit increase in BMI per kg/m^2^ was associated with the decrease of steps per day 182 (95%CI −356 to −8) among 44 children with CKD of all stages[12].

Height-adjusted lean body mass

Transplants

- Height adjusted lean body mass was positively correlated with PAQ score (r=0.49, P=0.047) among 17 children with transplants[41].
- After adjusted for age at transplant (beta 1.66, P=0.02) or age at DXA (beta = 1.55, p = 0.02), lower PAQ scores were significant predictors of lower height-adjusted lean body mass[41].
- Adjusted for annualized rate of percent eGFR, the positively relationship between height adjusted lean body mass and PAQ scores was not confirmed(beta=1.13 P=0.11) [41].

Lean mass Z-scores

CKD and dialysis

- Among 143 children with CKD and dialysis, per 1h/d increase in PA contributed to an increase in lean mass Z- scores; 0.05 increase in whole body (0.001, 0.10) (P = 0.06), 0.07 increase in trunk (0.02, 0.12) (P = 0.008) and 0.04 increase in leg (0.004, 0.09) (P = 0.07)[16].

Fat mass Z-scores

CKD and dialysis

- Among 143 children with CKD and dialysis, per 1h/d increase in PA contributed to a decrease in fat mass Z- scores; 0.05 decrease in whole body (0.11, 0.008) (P = 0.09), 0.05 decrease in trunk (0.11, 0.01) (P = 0.1), 0.06 decrease in leg (0.13, 0.002) (P = 0.04)[16].

**Growth**

Height standard deviation score (mean heights, SDS)

Transplant

- Similar mean heights (SDS) were found in 10 children with transplants exercising over 3h per week and 6 children with transplant exercising less than 3h per week, 1.86±0.66 VS 1.83±1.07, P>0.05[32].

Height percentile

Non-KRT CKD

- Among 224 children with CKD1-4, increased height percentile was associated with the number of the days in the past 7 engaged in 60 min of physical activity (β coefficient= 0.01, p = 0.03)[14].

Height Z-score

Non-KRT CKD

- 483 observations from 290 children with CKD1-4, at baseline year 1,3,5, height Z-score was positively related to the number of team sports in the prior 12 months (spearman’s R=0.14–0.23, P < 0.03)[45].
- Among 290 children with CKD1-4, aged above 12 years, the median height of the participants who played > 1 team sport was closer to the 50th percentile as compared with those who participated in ≤1 team sport for whom the median height was around the 25th percentile[45].

**Quality of life and psychosocial outcomes**

Transplants

- Among 20 children with transplant, PedsQL^TM^ version 4.0 Generic Core scales scores were lower in participants with no regular PA per week[37](no details provided).
- Among 20 children with transplants, PedsQL 4.0 scores were not related to total exercise time[27] (no details provided).

Quality of life

CKD of all stages

- Among 40 children with CKD (KRT included), PedsQL^TM^ questionnaire (version 3.0) scores were higher in 6 children practicing regular activity compared to 34 children having a sedentary life 69.2±13.5 VS 47.6± 14.9 (P=0.002)[11].
- Among 44 children with CKD (including KRT), the increase in steps per day predicted an increase of 0.15 (95% 0.04, 0.26) in physical functioning score on PedsQL[12].

Psychosocial outcomes

Transplants

- Among 20 children with transplants, higher PAQ activity scores were significantly correlated with perceptions of physical conditioning (r = 0.71, P= 0.001), sport competence (r = 0.64, P= 0.003), and body attractiveness (r = 0.55, P=0.019)[27].

**Kidney function**

Glomerular ﬁltration rate

Transplants

- Similar glomerular ﬁltration rate was found in 6 children with transplants exercising over 3 hours/week and 10 children with transplants exercising less than 3 hours/week. 91.83 ± 23.82 mL/min/1.73 m^2^ VS 93.1±22.21 mL/min/1.73 m^2^ (P>0.05)[32].
- eGFR (ml/min/1.73m2) in 25 paediatric transplant recipients with an inadequate level of moderate to vigorous intensity physical activity (MVPA: <420 minutes per week) were lower than 23 paediatric transplant recipients with adequate MVPA levels (MVPA: ≥420 minutes per week) 66.4 ± 28.4 and 88.1 ± 43.2, P = 0.04[29].
- DTPA-GFR was found not related to steps per day and mean PAQ score in 20 transplant recipients[27]. (no figure nor test was provided)

Non-KRT CKD

- Among 224 children with CKD1-4, lower eGFR (β coefficient =−0.02, p = 0.03) was independently associated with greater screen time[14].

CKD and dialysis

- Among 18 children with CKD 3-5 and those on dialysis in a cross-sectional study, PA energy expenditure was not associated with eGFR[17].

CKD worsening (transition to higher CKD stage or by the drop in estimated glomerular ﬁltration rate by a 25%)

- Before and after lockdown among children with CKD (not KRT), mean decrease of physical activity hours per week was -4.05 ± 6.0 among 11 children with worsening CKD progression with and was -4.35±4.19 among 10 children without worsening CKD progression. The difference was not significant, P = 0.90[40].
- Before and after lockdown among children with CKD (not KRT), the increase of screen time was 17.82± 8.49 hours per week in children with worsening CKD progression and was 13.85±8.53 hours per week in children without worsening CKD progression, P =0.30[40].

**Metabolic disorders**

Obese

Non-KRT CKD

- Among 224 children with CKD1-4, obesity was associated with a decrease in the number of days per week engaged in 20 min of PA (β coefficient = −0.75, p= 0.04) or 60 min of PA (β coefficient = −0.95, p = 0.01)[14].
- Among 224 children with CKD1-4, obesity (β coefficient = 1.04, p = 0.04) was independently associated with greater screen time[14].

Number of metabolic risk factors

Transplant

- Eighteen transplant recipients with 1 metabolic risk factor or less had a higher minute of MVPA compared to 4 transplant recipients with at least 2 risk factors (36 min vs 23 min P=0.02)[34].

Post-transplant metabolic syndrome components

Transplant

- Among 48 children with transplants, an additional 22 h per week of MVPA was related to one component less of post-transplantation metabolic syndrome in children with transplants(P=0.04)[29].

BP

Transplant

- Systolic BP and diastolic BP were not related to steps per day or PAQ scores in 22 children with transplants[27]. (no number and test were not provided)
- Among 48 children with transplants, 60 min more of VPA per week predicted a decrease of 0.18 in systolic BP z score (P=0.02), while amounts of MVPA or MPA had no significant effect on systolic BP z score (P=0.89)[29].

KRT

- Among 40 children with KRT, positive correlations were found between resting diastolic blood pressure and the average activity METs and the maximal activity METs (r=0.521; r=0.507; p<0.01)[18].

Biomarkers

CKD of all stages

- Among 44 children with CKD of all stages, the steps per day were related to the increase of haemoglobin per dg/dl 1039 (95%CI 539–1539) P<0.001[12].

Adverse event

Fracture

Non-KRT CKD

- Among 483 observations from 290 children with CKD (non-KRT), participation in more than 1 sport was associated with a nearly 5-fold higher risk of fracture (HR, 4.87; 95% CI, 2.21–10.75; P,0.001) during 5 year of follow-up. Participation in 1 team sport and more was associated with a HR of 2.35 (95% CI, 1.01–5.47; P=0.047)[45].

Injuries

Transplant

- In a study of children with kidney transplants, 51 injuries due to PA and 3 allograft injuries were reported within median follow-up time of 4.3 (2.4-6.6) years after transplantation. Total injuries per patient-year of transplant follow-up was 0.11 and allograft injuries per patient-year of transplant follow-up was 0.007[39]. None of these allograft injuries required hospitalization[39].
- One case study reported allograft injury when biking. The injury didn’t have any apparent long-term sequelae[66].

Dialysis

- Physicians didn’t report dialysis access damage or loss that was attributed to PA or sports participation[20].

Supplement 5 Interventions developed to promote or encourage physical activity among children and young people with chronic kidney disease

| Author and year | Design / participants / /intervention/ settings | Descriptions | Characteristics of intervention |
| --- | --- | --- | --- |
| **Interventions to Promote PA** | | | |
| Thorsteinsdottir 2018[52] | Historic control in transplant post-transplantation physiotherapy and health education  clinic | 22 in the 2008-2009 transplant cohort and 20 in the 2000-2015 transplant cohort; Early post-transplantation physiotherapy and focus on physical activity; 1-2 week after transplantation, regular physiotherapy 2-3 times per week in the inpatient and outpatient hospital until 3 months after transferring to local hospital for follow up. Health education was done to encourage children to exercise like their peers at each checkup. | frequency/duration  2-3 times a week or more  1-2 weeks after transplantation until 3 months after transferring to local hospital  personalization and progression  Individualized increments in intensity |
| Shroff 2019[59] | Non-RCT on dialysis  HD of HDF | 106 children on HD with age ranged from 5 -20 years received HD for 4-hour session, 3 times a week for a year.  71 children on HFD with age ranged from 5 -20 years received HDF for 4-hour session, 3 times a week for a year.  PA was assessed through self-report as one of secondary end-points. | Frequency/duration  for 4-hour session, 3 times a week for a year |
| Akber 2014[60] | Single arm study in CKD of all stages a combination of pedometers and behavioural consultation home | 44 children with CKD of all stages used pedometers to increase average daily step counts; and received education about the importance of the physical activity and weekly feedback on weekly targeted step counts, barriers to meeting target activity level, set a new goal for the upcoming week by telephone or email; | frequency/duration  12 weeks  personalization and progression  personalized by weekly contact with participant to provide behaviour consultation to reach weekly goal |
| Baqai 2025 [58] | RCT in CKD 2-4 with anaemia  Iron therapy | 10 children aged 12.8±6.2 received oral iron sulfate therapy for 3 months to increase PA measured by 10-item PROMIS paediatric proxy scale.  11 children aged 13.1 ±2.8 received usual care | Frequency/duration  3 months  personalization and progression  In the iron-therapy group, oral iron was given in the form of immediate-release iron sulfate, 3-6 mg/kg/day. |
| John 2020[61] | Single arm trial in CKD1-2  Group-based care | 10 adolescents aged 18 (IQR 16-19) received group-based care for 6 months to discuss self-activities supported by nephrologist or kidney dietitian | Frequency/duration  Group meeting with 2-5 adolescents per month for 6 months |
| **Exercise training programmes** | | | |
| Carbonera 2020[56] | RCT in transplant  traditional exercise_resistance_local muscle  home | 16 transplant children with 11.6 ± 3.7 years old; I: resistance training loaded inspiratory muscle training using a respiratory booster, inspiring through the device with enough force to overcome the threshold load, and then exhaling normally. 15 transplant children C with 11.7 ± 3.6 years old: placebo, inspiratory muscle training with threshold sham, with a minimum load (9 cm H2O) | intensity/repetitions/ sets  The inspiratory load was fortnightly adjusted to 40% of the previously measured MIP, repetitions: maintaining a respiratory frequency between 20 and 30 breaths per minute. Sets; 20 min a day； Rest: a 1-min interval during training, in case of fatigue  frequency/duration  seven times a week 6 weeks  personalization and progression  Personalized based on personal maximum inspiratory pressure; training load progressed (adjusted) fornightly during outpatient clinic visits to maintain 40% of the previously measured maximum inspiratory pressure |
| Weigmann-Fasbender 2020 [37], | Single arm study in transplant ehealth exercise_cardio and flexibility training  home | 13 children with transplants used home based exergaming intervention, Wii fit console of Nintendo and received regular contact with email and phone | frequency/duration  three times a week each session 30 mins 6 week a total of 18 sessions  personalization and progression  no personalization or progression |
| Cavusoglu 2023 [54] | RCT in CKD ehealth exercise_cardio and flexibility training  home | 8 children with CKD 1-5 C: home-based fun video exercises video recording. 5 mins of warmup, 30 min of aerobics, strengthening and yoga exercise, 5 min of cool down explained by a physiotherapist. | frequency/duration  twice a week each session 30 min 6 weeks with a total of 12 sessions |
| Cavusoglu 2023[54] | RCT in CKD ehealth exercise_cardio and flexibility training  clinic | 8 children with CKD 1-5 I: Supervised Nintendo Wii-based exergaming, mixed (cardio + flexibility) stretching + breathing 5 min 30 min of aerobics, stretching and yoga exercises with Nintendo Wii Fit 5 min cool down exercise supervised by a paediatric physiotherapist in a clinical setting. | frequency/duration  twice a week each session 30 min 6 weeks with a total of 12 sessions  personalization and progression  intensity was personalized and progressed based on the personal progress by increasing the number of repetitions or the duration of the exercise. |
| Abd-Elmonem 2019[53] | RCT in CKD 3 - 4 traditional exercise_resistance_full body  clinic | 16 children with CKD 3-4 received supervised progressive resistance exercises, including warm up, resisted exercise, and cool down.  16 children with CKD 3-4 received non standard medical care no change of daily activities | frequency/duration  twice a week giving an interval of 48 hours between sessions. each session 60 min 6 months, a total of 48 sessions  personalization and progression  the intensity of the exercise was personalized individually at 60-75% of 1 RM. Depending up on the child exercise tolerance, each set was performed 10 gradually increased to 15 repetitions The intensity (resistance) of the resisted exercise increased gradually in three phases. During phase 1 (first two months) the weight used was determined according to the RM. In phase two (the second two months) the weight increased by 0.25 kilogram. During the third phase (the last two months) the weight increased by 0.5 kilogram.  the repetition also increased from 10 to 15. |
| Feldkötter 2021 [55] | RCT in HD traditional exercise_cardio intradialytic | 23 children on HD aged 14.0 ± 3.4 years had supervised bicycle ergometer training during the haemodialysis for 12 weeks and extended to another 12 weeks. 22 children on HD aged 15.1± 2.4 years had none for 12 weeks and then received the intervention for 12 weeks | intensity/repetitions/ sets  first 30 min of HD  warm-up Endurance training cool down  frequency/duration  three times a week each session 50 - 70 mins  12 weeks 36 sessions  personalization and progression  the training was personalized with 70 - 80% of the patient specific maximum workload which was tested by endurance testing scheduled at the study visits using ergometric testing. This test was always performed on dialysis days prior to start of HD. The tests were conducted at the baseline and 3 months (end of the first 12 week intervention) and 6 months after(end of the 12 week intervention in the control group.) no progression within 12 week training block |
| Khalf-allah 2024[57] | RCT in HD traditional flexibility + resistance intradialytic exercise  clinic | Study group: 30 children on HD received a 40-min exercise program 3 times a week during HD for 2 months. Each session consisted of 20-minute muscle stretching and 20-minute isometric exercise.  Control group: 30 children on HD received usual care | intensity/repetitions/ sets  Children did not do any activity in the first hour.  Stretching exercises in the first 20 min of the second hour of haemodialysis. This involved flexing or  stretching the calf, gastrocnemius, soleus, hamstring, and quadriceps muscles at a frequency of 10 times per session.  In the first 20 min of the third hour, the children received isometric exercise. This type of strength training involved contracting the muscles (such as the chest, biceps, stomach, and hip abductor and  adductor) for 3–5 s and then relaxing them at a frequency of 10 times per session  frequency/duration  40 minute per sessions, 3 days a week for 2 months.  personalization and progression  Depending on the child’s exercise tolerance, each set was performed with 10 repetitions, gradually increasing to 15 repetitions. |
| Goldstein 2009 [63] | Single arm study in HD traditional mixed cardio and resistance intradialytic exercise | 10 children on HD with median age of 13.6 (8-25) pilot intervention of Warming up by arm circles; kicking ball, overhead and chest passes with light ball;  Leg/arm bike Dumbbell weights (3~10 lb) Theraband exercise; Medicine ball tossing Wrist/ankle weights | intensity/repetitions/ sets  The heart rate was monitored and graphic data was obtained during each session to keep patients within aerobic heart rate zones. For weights and theraband 10~ 15 repetitions Warm up 5~ 10min and leg/arm bike 10~ 20 min  frequency/duration  twice a week each session 30~60 min last for 3 months 24 sessions in total  personalization and progression  the training intensity was personalized using the heart rate monitor during the exercise to make sure the exercise was within the aerobic zone;  the type of exercise was personalized considering the depending on the location and placement of HD access, blood pressure cuff and heart rate monitor leads. The theraband training started from yellow and progress to the orange, green and blue, ending with purple if patients could complete 10 repetitions without difficulty. |
| Paglialonga 2014 [64] | Single arm study in HD traditional cardio intradialytic exercise | 10 children on HD with median age of 15.6 (8-25) cycloergometer with resistance during the first half of the HD sessions( the second hour of treatment) included warmup bike and cool down | intensity/repetitions/ sets  2 to 3 mins of warm up and ended 2 to 3 mins cool down.  frequency/duration  two three times a week depending on the availability of physiotherapist each session 30 min 3 months in total of 24 to 36 sessions  personalization and progression  The intensity of the bike exercise - the cycloergometer resistance was personalized (adjusted) on the basis of heart rate and subjective Borg scale at 3 the exercise duration was 10 min for the first session and increased up to 30 min within a few sessions.  the exercise intensity was progressively increased. |
| van Bergen 2009 [62] | Single arm study in dialysis traditional mixed cardio and resistance  combined (clinic + home) | 5 children on dialysis aged 12.6 years old received supervised graded community-based exercise program, including warm up- aerobic training, resistance exercises, and active games – cool down; starting after the sixth week of the intervention, patients were instructed to perform exercises at home once a week | intensity/repetitions/ sets  5 mins of warmup; aerobic training with intensity from 55-90% of HR peak, resistance exercises, and active games. The session ended with 5 mins of cool down exercise. The resistance training was not with the heavy weights. frequency/duration  twice a week each session 50 min 12 weeks a total of 24 sessions  personalization and progression  the exercise intensity was individually prescribed for each patient based on the data from the baseline exercise test, aerobic training (with an intensity ranging from 55–90% of HRpeak).  The progressions were at the sixth week, patients would do exercise once a week at home; and in the final 4 weeks, 10mins of interval exercise was included. |
| Ramírez-Senent 2023 [65] | Case series in pre-haemodialysis  traditional resistance_local muscle  home | 4 children with predialysis received isometric exercise program, 30 contractions with a hand grip devices, maintaining 3 seconds each contraction, and twice a day, | frequency/duration  Twice a day each contraction at least three seconds until the day before surgery 5,23,16,10 weeks respectively, 13 weeks for median duration, ranged from 10 to 46 weeks.  personalization and progression  The device was previously adjusted to half of their maximum strength, measured with a dynamometer with 30 repetitions. |

*CKD , chronic kidney disease; HD, haemodialysis.; min, minutes;

Supplement Table 6 Outcomes of interventions to promote physical activity among children and young people with CKD

**Intervention with PA promotion strategies**

**Interventional study with historic control**

Transplant

- Twenty children with transplants have a higher median (range) VO_2_ peak 44.3 (31.7, 58.8)ml/kg · min compared to 22 children with transplants from previous cohort 33.5 (18, 59)ml/kg · min after implementing post-transplantation physiotherapy and health education at clinics[52].

**PA as primary or secondary interventions**

- In a pilot RCT, ten children with CKD 2-4 with anaemia received iron therapy and 11 children didn’t receive iron therapy for 3 months. PA was measured by patient-reported outcomes measurement information system (PROMIS) Parent Proxy Items as the primary outcomes. There was a trend toward an increase of PA in both groups, **with no significant different between groups**. There were no noticeable changes in muscle mass and eating behaviour, as well as reduction of strength before and after intervention in both groups. There was a trend toward a reduction of fatigue in the iron-therapy group [58].
- In a non-randomised controlled trial, 106 and 71 children were on HD and HDF for 4 hour per session 3 times per week for a year. PA was assessed through self-report as one of the secondary end-points. It was categorised as in a wheelchair, walks < 30 mins per day, walks > 30 mins per day, and play sports**. At 12 months, 44% children on HDF playing sports compared with only 13% of children on HD (P<0.01)**, however, the baseline PA levels were not reported[59].
- In a single-arm trial, children with CKD across stages received a combination of pedometers and behavioural consultation to increase daily step counts for 12 weeks. Mean daily steps, measured using pedometers, remain the similar level before and after intervention (+48, 95% CI −48 to +145 steps/day per week). Transplant recipients and patients with CKD increased their activity by 100 steps/day (95% CI −14 to 208) and 73 steps/day (95% CI −115 to 262) each week, respectively, and patients on dialysis decreased by 133 steps/day (95% CI −325 to 58; p-value for interaction 0.03[60].
- In a single-arm trial, ten adolescents with CKD1-2 received group meeting per month for 6 months to discuss self-activities and increase PA. With suboptimal attendance and adherence of wearing accelerometers, median steps per hour increased from 499 (IQR, 111, 712) to 699 (IQR, 302, 1107) at 6 months in 5 adolescents who completed the baseline and 6 months measurement[61].

**Feasibility (exercise intervention)**

Dialysis (including pre-dialysis)

- Five (20%) of 20 children on dialysis completed an average of 22 sessions of a supervised graded community-based exercise (total sessions of 24). Fifteen patients did not complete the program and took part in an average of 7 sessions. Seven of them were due to a combination of lack of time and motivational problems. Six of them were due to medical problems because of kidney disease. One received transplantation during the study and another because of family holidays[62].
- Ten (50%) of 20 children on haemodialysis completed a twice a week intradialytic cardio and resistance training[63].
- Eight (80%) of 10 children on haemodialysis completed an intradialytic cycloergometer training. (Other 2 children underwent kidney transplantation during the study period). The median number of exercise sessions/patient was 28.5 (range 23-32). (total session of the protocol ranged 24 to 36). All 8 participants claimed that the training was enjoyable and the time on HD passed quickly and that they would like to continue the program[64].
- Four (100%) children with 2 in pre-dialysis and 2 in haemodialysis who were not eligible for arteriovenous fistula (AVF) received isometric exercise program for 5 to 23 weeks[65].

**Cardiorespiratory Fitness (exercise intervention)**

VO_2_max

**Single arm trial**

Transplant

- VO_2_max remained unchanged in 13 transplant recipients after home-based exergaming intervention (Wii fit console of Nintendo) three times a week for 6 weeks[37]. (Number was not provided)

Dialysis

- Mean VO_2_max increased by 12.6 ± 10.3 ml/kg · min in 5 children on dialysis after a supervised graded community-based exercise program twice a week for 6 weeks[62]. (Inference test was not provided)

Six-minute walking distance (6MWD)

**RCT**

Transplant

- The change of 6MWD in 16 children with transplants after a 6-week loaded inspiratory muscle training between 15 children with transplants training with threshold sham was consistent with chance[56](numbers was not provided).

Non-KRT CKD

- The change of 6MWD between 8 children with CKD 1-5 after a 12-session supervised Nintendo Wii-based exergaming and 8 children with CKD after a 12-session home active video was consistent with chance (P=0.92). Within groups, 6MWD increased from 502.56 ±116.70 meters to 585.81 ±99.85 meters, P = 0.012 and 522.12±110.92m to 609.31±100.29 m, P =0.012, respectively[54].
- The change of 6MWD in 16 children with CKD3-4 after a 6-month supervised progressive resistance exercise was higher compared with 16 children with CKD with no daily PA intervention, 522.5 ±32.96 VS 430 ±25.03 meters (P=0.0001)[53].

**Single arm trial**

Dialysis

- The mean 6MWD tended to be lower after intervention with mean change 0.9±6.8 meter. (only descriptive, inference test not provided) in 5 boys on dialysis after a supervised graded community-based exercise twice a week for 12 weeks[62].
- Mean 6MWD increased from 589±90 yards to 627 ± 86 yards in 10 children on HD after 3 month of an intradialytic cardio and resistance training[63].
- Median (range) 6MWD increased from 552.5 (440-640) to 585.0(480-650) meters in 8 children on HD after a 3 month of an intradialytic cycloergometer training. The increase in median of 6MWD was 4.9% (P <0.01)[64].

CKD of all stages (discrete data by transplant and dialysis)

- The mean 6MWD step per day increased 16 (95%CI 4, 28) in 44 children with CKD1-5 including KRT. Noted, thirty-three children with CKD 1-5 including KRT after wearing pedometers for 3 months and other 11 children didn’t provide full 3 months data. All available were used in analyses. Besides, different treatment modalities responsed differently. Transplant recipients and children on dialysis increased their 6MW distance by 31m (95%CI 19,42) P<0.0001 and -28m (95% CI -60, 5) P=0.008. The difference between transplant and dialysis in increase in 6MWT was significant (-35m 95%CI -58, -12, P=0.003); 6MW distance improved among transplant recipients who increased their daily steps more than 1000 steps compared with those who did not (37m 95%CI 26,71 VS 11m 95%CI -6.5, 30.5), P= 0.006)[60].

**Muscle strength (exercise intervention)**

Grip strength

**Single arm trial**

Transplant

- Among 20 children with transplants, there was a tendency for a greater handgrip strength in moderately active subjects. (no details provided)[37].

Dialysis

- The change of muscle strength tended to be higher after intervention with mean change 2.4±20 kg in 5 children on dialysis after supervised graded community-based exercise program twice a week for 12 weeks (only descriptive, inference test was not conducted)[62].
- The change of grip strength was consistent with change from 23.2 ±10.9 to 26.6 ± 12.4 kg in 10 children on HD after an intradialytic cardio and resistance training twice a week for 3 months, P = 0.07[63].

Lower/upper extremity strength

**RCT**

Non-KRT CKD

- The increase in mean strength of left knee flexion in 8 children with CKD after a 12-session supervised Nintendo Wii-based exergaming was higher than 8 children with CKD after a 12-session home active video (2.46±2.18 VS -0.04 ±1.92, P=0.037)[54]. Muscle strength in right and left shoulder abductor and flexor, right hip abductor, and right knee flexor improved in each group[54].

Maximal inspiratory pressure

**RCT**

Transplant

- The maximal inspiratory pressure increased by 35% and the maximal expiratory pressure increased by 26% in 16 children with transplants after a 6-week loaded inspiratory muscle training, compared with 5 and 4% in the control group of 15 children with transplants training with threshold sham (P=0.01, P=0.046)[56].

**Single arm trial**

Dialysis

- The change of lower extremity testing increased from 70± 32 to 94 ± 42 newton-meters/second in 10 children on HD after an intradialytic cardio and resistance training twice a week for 3 months, P = 0.003[63].
- The median (range) of lower extremity strength increased by 29.3 (6-37.5)% from 10 (5-15) kg to 15 (5.3-20)kg, P =0.02 in 10 children on HD after a 3-month intradialytic bike training program. The median (range) number of the stands increased by 19% (11.1-44.8) from 39 (18-47) and 41.5 (20-60), P= 0.01[64].

**Quality of life (exercise intervention)**

Physical functioning score

**Single arm trial**

CKD of all stages

- Self-report physical functioning did not change significantly (-7, 95%CI -15 to +1 points) from baseline 72.3±17.3 in 44 children with CKD including KRT wearing pedometers for 12 weeks; CKD category was not significantly associated with change in physical functioning (P=0.18)[60].

Overall Quality of life score

**Single arm trial**

Transplant

- The similar quality of life scores were found in 13 transplant recipients after home-based exergaming intervention (Wii fit console of Nintendo) three times a week for 6 weeks (detailed number and P value were not provided). The complying patients showed an average increase of 7% in health-related quality of life. (P value was not provided)[37].

Dialysis

- HRQOL tended to be unchanged with mean change 0.0±4.5 in 5 children on dialysis after a supervised graded community-based exercise program twice a week for 6 weeks (only descriptive, inference test was not offered)[62].

**Historic control**

Transplant

- PedsQL score increased from 69.10 ± 17.98 in 22 children with transplants from previous cohort to 80.56 ± 12.61 in 20 children with transplants after implementing post-transplantation physiotherapy and health education at clinics P = 0.003[52].

**RCT**

Transplant

- Similar increase of PedQL child self-report scores in 8 children with CKD after a 12-session supervised Nintendo Wii-based exergaming and in 8 children with CKD after a 12-session home active video was consistent with chance (14.84 ± 14.34 VS 11.60 ± 8.97, P=0.72). Within groups, Physical health summary score reported by children improved from 58.93 ±21.09 to 73.82 ±18.44 in first intervention group(P=0.018) and from 67.4±36.61 to 79.01±30.65 in the second group, P=0.028[54].

Haemodialysis

- PedsQL scores were similar in 23 children on HD after a supervised intradialytic bicycle ergometer training for 12 weeks compared with control group 22 children on HD [55] (training group from 74.9±15.7 to 77.8±12.3, control group from 81.3±11.8 to 80.1 ±10.9 in control group).
- Thirty children from the study group had a higher proportion of reporting good QoL compared with control group (66.7% VS 3.3%, P=0.001) after 40 minute flexibility and resistance intradialytic exercise 3 times a week for 2 months. (QoL by PedsQLTM scale. Poor QoL defined as score less than 50%, 50%-75% fair and 75%-100% good.)[57]

Non-KRT CKD

- Mean PedsQL score was 67.24±3.43 in the intervention group (16 children with CKD after full body resistance training for 3 months), higher than the score of 53.81±2.22 in the control group (P= 0.0001)[53].

**Steps (exercise intervention)**

**Single arm trial**

Transplant

- The steps increased by 121 per hour (approximately 31%, P = 0.043) in 13 transplant recipients after home-based exergaming intervention (Wii fit console of Nintendo) three times a week for 6 weeks [37]

CKD of all stages (discrete data by transplant and dialysis)

- The steps remained unchanged (+48, 95% CI −48 to +145 steps/day per week) in 44 children with CKD1-5 including KRT. Noted, thirty-three children with CKD 1-5 including KRT after wearing pedometers for 3 months and other 11 children didn’t provide full 3 months data. Transplant recipients and patients with CKD increased their activity by 100 steps/day (95% CI −14 to 208) and 73 steps/day (95% CI −115 to 262) each week, respectively, and patients on dialysis decreased by 133 steps/day (95% CI −325 to 58; p-value for interaction 0.03)[60].

**RCT**

Non-KRT CKD

- Compared with 8 children with CKD after a 12-session home active video (Intervention II), steps per day increased in 8 children with CKD after a 12-session supervised Nintendo Wii-based exergaming(Intervention I) (P=0.021). Within groups, the steps per day increased from 3792.87±1362.74 to 4486.12±1367.08 steps in intervention II (P=0.017) and increased from 4788.12 ± 1919.61 to 6648.25±2389.47 steps, P=0.021[54].

**Other health-related outcomes (exercise intervention)**

Fatigue

**Single arm trial**

Dialysis

- The three children on dialysis after a supervised graded community-based exercise program, showed a decreased perceived fatigue (Checklist Individual Strength-20 (CIS-20) [62]. (only descriptive)

**RCT**

Non-KRT CKD

- Similar reduction of fatigue measured by fatigue scale (VFS) were found in 8 children with CKD after a 12-session supervised Nintendo Wii-based exergaming (Intervention I) and 8 children with CKD after a 12-session home active video (Intervention II) (-3.12 ± 2.94 VS -1.62 ± 1.59, P=0.335). Within groups, fatigue measured by visual fatigue scale (VFS) decreased from 4.5±3.4 to 1.37 ±1.76 (P=0.028) in intervention I and decreased from 3±3.11 to 1.37±1.84 in intervention II (P=0.042)[54].
- Lung function

**Single arm trial**

Dialysis

- Lung function in 8 children on HD after a 3 month intradialytic cycloergometer training remained unchanged. The median (range) forced vital capacity, FVC(% pred) before and after intervention were 87.2 (77.9-100.1) VS 89.8 (84.6-130.2), decreased 1.2 (-4.8, 5.0), P = 0.46; The median (range) forced expiratory volume, FEV(% pred) were 85.4 (68.7–126.7) VS 95.2 (80.5–120.6) increased 1.9 (-4.8, 17.2), P=0.17; The median (range) peak expiratory flow PEF(%pred) were 75.5 (61.2–105.1) VS 78.7 (67.7–91.6) , increased 10.8 (-23.4, +19.6), P=0.92[64].

**RCT**

Transplant

- Similar pulmonary function measured by spirometry were founded in 16 children with transplants after a 6-week loaded inspiratory muscle training and 15 children with transplants training with threshold sham. Forced expiratory volume in the first second, FEV1 was 2.15 ± 1.04 VS 1.94± 0.86, P =0.55, FEV1 predicated was 2.09± 1.05 VS 2.16 ± 0.99, P =0.86, forced vital capacity, FVC was 2.45 ± 1.22 VS 2.24 ± 1.10, P =0.63, FVC predicated was 2.24 ± 1.10 VS 2.31 ± 1.02, P=0.86[56].

Motor coordination

**Single arm trial**

Transplant

- The motor coordination remained unchanged in13 transplant recipients after home-based exergaming intervention (Wii fit console of Nintendo) three times a week for 6 weeks[37]. (number was not provided.)

Mental health: Strength and difficulties SDQ scores (The Strengths and Difficulties Questionnaire)

**Single arm trial**

Transplant

- Strength and difficulties SDQ scores decreased from 11.58±5.69 to 7.80 ± 5.16 in 20 children with transplants after implementing post-transplantation physiotherapy and health education at clinics, (P=0.012)[52].

**Disease-related outcomes (exercise intervention)**

Single pool Kt/V

Dialysis

**RCT**

- Similar single pool Kt/V was found in 23 children on HD after a supervised intradialytic bicycle ergometer training for 12 weeks and 22 children on HD as a control group. Single pool Kt/V at baseline in the intervention group was 1.7 (0.33) in intervention group and 1.8(0.55) in control group. Single pool Kt/V 12 weeks after in the intervention group was 1.7Single pool Kt/V at both timepoints and 12 weeks after were not different between two groups (P=0.49, P= 0.91)[55].

Biomarkers

**RCT**

Transplant

- Haemoglobin improved in 16 children with transplants after a 6-week loaded inspiratory muscle training (the intervention group, increased from 12.17±0.35 to 13.29 ± 0.42 g/dl), compared with 15 children with transplants training with threshold sham (the control group, increased from 11.96±0.36 to 12.04 ± 0.44 g/dl, P=0.047). Creatinine, urea, potassium, and phosphorus levels did not show any significant changes at any time during the evaluation[56].

Radiocephalic ﬁstula creation

**Case series**

Pre-dialysis

- Four cases of successful radiocephalic ﬁstula creation after isometric exercise program for 5 to 23 weeks in 2 children in pre-dialysis stage and 2 children on haemodialysis who were not eligible for arteriovenous fistula (AVF)[65].

**Adverse events (exercise intervention)**

**RCT**

Dialysis

- Similar number and severity of AEs and serious adverse events were found in 23 children on HD after a supervised intradialytic bicycle ergometer training for 12 weeks and 22 children on HD as a control group[55].
